# Identification, In Silico Characterization, and Differential Expression Profiles of Carotenoid, Xanthophyll, Apocarotenoid Biosynthetic Pathways Genes, and Analysis of Carotenoid and Xanthophyll Accumulation in *Heracleum moellendorffii* Hance

**DOI:** 10.3390/ijms23094845

**Published:** 2022-04-27

**Authors:** Ramaraj Sathasivam, Nam Su Kim, Minsol Choi, Haejin Kwon, Bao Van Nguyen, Jae Kwang Kim, Dae Hui Jeong, Eung Jun Park, Hong Woo Park, Sang Un Park

**Affiliations:** 1 Department of Crop Science, Chungnam National University, 99 Daehak-ro, Yuseong-gu, Daejeon 34134, Korea; ramarajbiotech@gmail.com (R.S.); minsolll@cnu.ac.kr (M.C.); kwonhaejin42@o.cnu.ac.kr (H.K.); 2 Korea Research Institute of Bioscience and Biotechnology, 30 Yeongudanji-ro, Ochang-eup, Cheongju-si 28116, Korea; kns917555@kribb.re.kr; 3 Department of Smart Agriculture Systems, Chungnam National University, 99 Daehak-ro, Yuseong-gu, Daejeon 34134, Korea; nguyenvanbao@tuaf.edu.vn; 4 Division of Life Sciences, College of Life Sciences and Bioengineering, Incheon National University, 119 Academy-ro, Yeonsu-gu, Incheon 22012, Korea; kjkpj@inu.ac.kr; 5 Forest Medicinal Resources Research Center, National Institute of Forest Science, Yeongju 36040, Korea; najdhda@korea.kr (D.H.J.); pahkej@korea.kr (E.J.P.)

**Keywords:** *Heracleum moellendorffii* Hance, carotenoid pathway genes, xanthophyll pathway genes, apocarotenoid pathway genes, gene expression, carotenoid, xanthophyll

## Abstract

*Heracleum moellendorffii* Hance is a non-woody forest plant widely used in China, Korea, and Japan because of its various therapeutic properties. However, the genetic details of the carotenoid pathway (CP), xanthophyll pathway (XP), and apocarotenoid pathway (AP) genes have not been studied. Thus, the CP, XP, and AP genes of *H. moellendorffii* were detected and analyzed. A total of fifteen genes were identified, of which eight, four, and three belonged to CP, XP, and AP, respectively. All identified genes possessed full open reading frames. Phylogenetic characterization of the identified gene sequences showed the highest similarity with other higher plants. Multiple alignments and 3D dimensional structures showed several diverse conserved motifs, such as the carotene-binding motif, dinucleotide-binding motif, and aspartate or glutamate residues. The results of real-time PCR showed that the CP, XP, and AP genes were highly expressed in leaves, followed by the stems and roots. In total, eight different individual carotenoids were identified using HPLC analysis. The highest individual and total carotenoid content were achieved in the leaves, followed by the stems and roots. This study will provide more information on the gene structure of the CP, XP, and AP genes, which may help to increase the accumulation of carotenoids in *H. moellendorffii* through genetic engineering. These results could be helpful for further molecular and functional studies of CP, XP, and AP genes.

## 1. Introduction

*Heracleum moellendorffii* Hance is a perennial shade herb that belongs to the Apiaceae family and is widely distributed in China, Korea, and Japan [1]. It is mainly grown in forested areas and is commonly used as a wild vegetable because of its high nutritional value and trace elements. *H. moellendorffii* has long been used to treat rheumatoid arthritis and colds [1,2], and the roots have been used as traditional medicine for healing various inflammatory diseases, including back pain, arthritis, and fever [3,4]. Few studies have reported that the active ingredients in *H. moellendorffii* leaves had anti-melanogenesis and antioxidant activity [2,3,4,5]. Recent studies have reported that the root of *H. moellendorffii* exerts immunostimulatory and anti-inflammatory effects [4,6,7]. The crude extract of *H. moellendorffii* has been used to control certain agricultural diseases caused by pests (*Sitophilus zeamais* Motsch. (maize weevil) and *Tribolium castaneum* Herbst (red flour beetle)) [1,8]. Several studies have reported that the chemical constituents of *H. moellendorffii* are coumarins, flavonoids, sesquiterpenoids, monoterpenoids, and polyacetylenic compounds [8]. In addition, the chemical constituents of the essential oil present in the roots of *H. moellendorffii* have been studied [8].

Carotenoids are natural pigments present mainly in vegetables and fruits. They also play a vital role in plant cells by scavenging reactive oxygen species (ROS) and protecting cells against oxidative stress [9]. They have numerous health benefits, including antidiabetic, antibacterial, anti-aging, anticancer, anti-inflammatory, immunomodulatory, and neuroprotective effects [10,11,12,13,14,15]. Moreover, carotenoids can boost immune cell function [16]. Recently, several researchers have identified that medicinal plants contain several natural carotenoids [9,17,18,19,20]. Owing to their various pharmacological activities, it is essential to identify, screen, and analyze different types of carotenoids [21]. Among the different types of carotenoids, β-carotene has been widely studied because of its numerous health benefits. However, other carotenoids gained attention in recent years because they play a vital role in the human diet [22]. The details of CP, XP, and AP and the enzymes required for conversion in each step have been extensively studied in plants, and several reviews have been published by Stanley and Yuan [23] and Sathasivam et al. [9].

The CP, XP, and AP genes have been identified and characterized in plants such as Arabidopsis, Ixeris dentate, Brassica rapa subsp. pekinensis, Carica papaya, Chelidonium majus, Citrus limon Burm.f., Citrus sinensis Osbeck, Citrus unshiu Marc., Fragaria × ananassa Duch., Lycium chinenses, Nasturtium officinale, and Scutellaria baicalensis [24,25,26,27,28,29,30,31,32]. However, few reports have been published regarding the molecular aspects of H. moellendorffii [33,34,35]. In addition, no studies have been carried out regarding the identification, in silico characterization, and gene expression profiling of carotenoid pathway (CP), xanthophyll pathway (XP), and apocarotenoid pathway (AP) genes in H. moellendorffii. The main aim of this study was to identify and characterize the CP, XP, and XP genes from the H. moellendorffii transcriptome data constructed in our laboratory.

This is the first report to identify and characterize CP (*HmGGPS, HmPSY*, *HmPDS*, *HmZ-ISO*, *HmZDS*, *HmCrtISO*, *HmLCYB*, and *HmLCYE*), XP (*HmCHXB*, *HmCHXE*, *HmZEP*, and *HmVDE*), and AP genes (*HmCCD* and *HmNCED*) in *H. moellendorffii*. To confirm the spatial distribution of the CP, XP, and AP gene transcripts, we analyzed gene expression in different organs of *H. moellendorffii*. Moreover, we analyzed and identified eight individual carotenoid compounds distributed in various parts of *H. moellendorffii*. This study provides immense knowledge on the CP, XP, and AP genes and the accumulation of carotenoids in different organs of *H. moellendorffii*, which are beneficial to human health. In addition, our results improve our knowledge of the CP, XP, and AP genes and enable us to investigate strategies that could enhance the anti-carcinogenic properties of *H. moellendorffii*.

## 2. Results and Discussions

### 2.1. Mining and Sequence Analysis of CP, XP, and AP Genes

**Figure 1:**
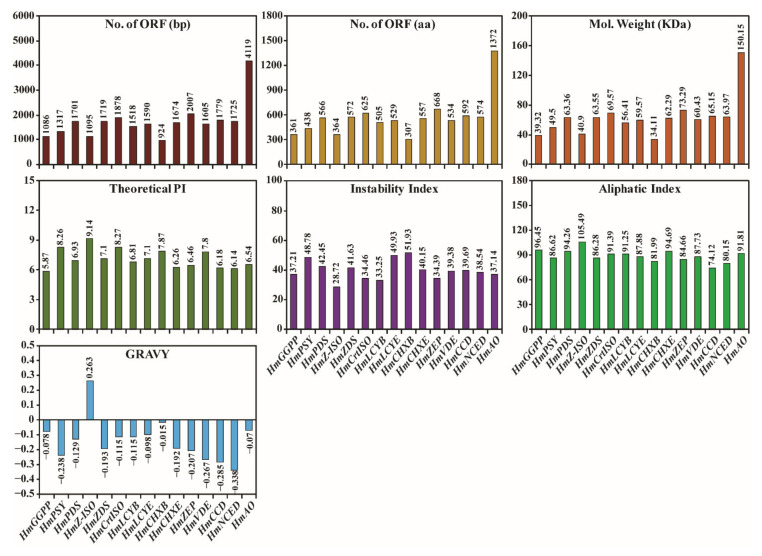
Physiochemical characterization of 15 CP, XP, and AP genes in *H. moellendorffii*.

The CP, XP, and AP genes of *H. moellendorffii* were mined from transcriptomic data acquired in our laboratory. The retrieved genes were searched for using BLASTN. The results showed that all retrieved gene sequences had high similarity with other higher plant sequences. To identify the full open reading frame (ORF) in the retrieved sequences, the sequences were analyzed using the ORF finder program in the NCBI database. From the transcriptomic data, nucleotide sequences of different lengths were obtained for each gene, and genes that possessed the highest number of nucleotides and full ORFs were used for structural and functional analyses. A total of 15 full ORFs genes were mined, of which 8 CP (*HmGGPS*, *HmPSY*, *HmPDS*, *HmZ-ISO*, *HmZDS*, *HmCrtISO*, *HmLCYB*, and *HmLCYE*), 4 XP (*HmCHXB*, *HmCHXE*, *HmVDE*, and *HmZEP*), and 3 AP (*HmCCD*, *HmNCED*, and *HmAO*) genes were identified (Figure 1). The above sequences were submitted to GenBank with the following accession numbers: 8 CP (OM732401, OM732402, OM732403, OM732404, OM732405, OM732406, OM732407, and OM732408), 4 XP (OM732409, OM732410, OM732411, and OM732412), and 3 AP (OM732413, OM732414, and OM732415). Moreover, several physicochemical parameters were analyzed using the free online ProtParam software (Figure 1). The estimated MWs and isoelectric points of the retrieved CP, XP, and AP sequences are shown in Figure 1. The MWs of the CP, XP, and AP genes ranged from 34.11 kDa (HmCHXB) to 150.15 kDa (HmAO). The isoelectric points ranged from 5.87 to 9.14. The predicted MW and isoelectric points were more or less similar to other higher plant species, such as *I. dentate* [28], *Brassica napus* [36], *L. chinensis* [27], *C. majus* [19], *S. baicalensis* [31,37], and *N. officinale* [18]. 

The highest aliphatic and instability indices of the identified AA sequences were for *HmZ-ISO* (105.49) and *HmCHXB* (51.93), whereas the lowest were observed in *HmCCD* (74.12) and *HmZ-ISO* (28.72), respectively. The relatively high aliphatic index values indicated that all enzymes were thermostable over a wide range of temperatures [38]. In addition, instability index analysis showed that *HmZDS* and *HmZEP* enzymes were more stable than *HmPDS*, *HmCHXB*, and *HmCHXE*. GRAVY indices of the AA sequence were used to identify the hydrophilicity and hydrophobicity of the protein. The results of the GRAVY index analysis showed that most of the CP, XP, and AP pathway genes possessed negative values, indicating that most of these enzymes were hydrophilic. In contrast, *HmZ-ISO* was hydrophobic with a positive GRAVY value (0.263). Negative GRAVY index values indicated that these enzymes interacted better with water (Figure 1). A similar result was obtained with microalgae, indicating that these enzymes are hydrophobic [39]. Signal IP analyses revealed that *HmZDS* had the highest original shearing site score (C score), followed by *HmLCYB*, *HmCHXE*, *HmPSY*, *HmZEP*, *HmCCD*, *HmCrtISO*, *HmZ-ISO*, *HmAO*, *HmNCED*, *HmCHXB*, *HmVDE*, *HmPDS*, and *HmLCYE*. *HmCHXE* had the highest synthesized shearing site score (Y score), followed by *HmPSY*, *HmZDS*, *HmZEP*, *HmLCYB*, *HmCrtISO*, *HmVDE*, *HmLCYE*, *HmZ-ISO*, *HmPDS*, *HmCCD*, *HmNCED*, *HmCHXB*, and *HmAO* also had the highest signal peptide score (S score), followed by *HmCHXE*, *HmPSY*, *HmZEP*, *HmCrtISO*, *HmVDE*, *HmZDS*, *HmLCYE*, *HmZ-ISO*, *HmPDS*, *HmCCD*, *HmCHXB*, *HmNCED*, *HmAO*, and *HmLCYB* (Table S1). None of these genes possesses any signal peptide transmembrane region (Table S1) or cleavage site (data not shown). In several other higher plants such as *Brassica napus* [40], *Musa acuminate* [41], *C. majus* [19], *Triticum aestivum* [42], *N. officinale* [18], and the marine green algae *Tetraselmis suecica* [15], similar results regarding the signal peptide transmembrane regions in the CP genes were reported. In addition, transmembrane helices were predicted for all enzymes using online free TMHMM software. The results showed that the enzymes *HmZ-ISO*, *HmCHXB*, and *HmLCYE* consisted of six, three, and one transmembrane helices, respectively, whereas none of the other enzymes contained any transmembrane helices (Table S1). This result was in agreement with the experimental study of *D. salina*, which reported that *ZDS* is not a potential membrane protein [43]. Subsequently, NetNGlyc, PSIPRED, and radar tools were used to predict the N-glycosylation sites, disordered regions, and internal repeats of the CP, XP, and AP proteins (Table S2). In addition, secondary structure analysis of CP, XP, and AP genes showed that most of the genes were rich in alpha-helices. In contrast, *HmCrtISO*, *HmZEP*, *HmVDE*, *HmCCD*, *HmNCED*, and *HmAO* were rich in random coils, indicating that these proteins might be essential for flexibility and conformational changes during catalysis (Table S2) [44]. Narang et al. [39] also reported that most CP genes in microalgae contained a higher percentage of alpha-helices than extended strands and random coils. These results support the sequence analysis results that the CP, XP, and AP genes possess highly conserved AA sequences in higher plants and green algae.

### 2.2. CDD and Motif Analysis

**Figure 2:**
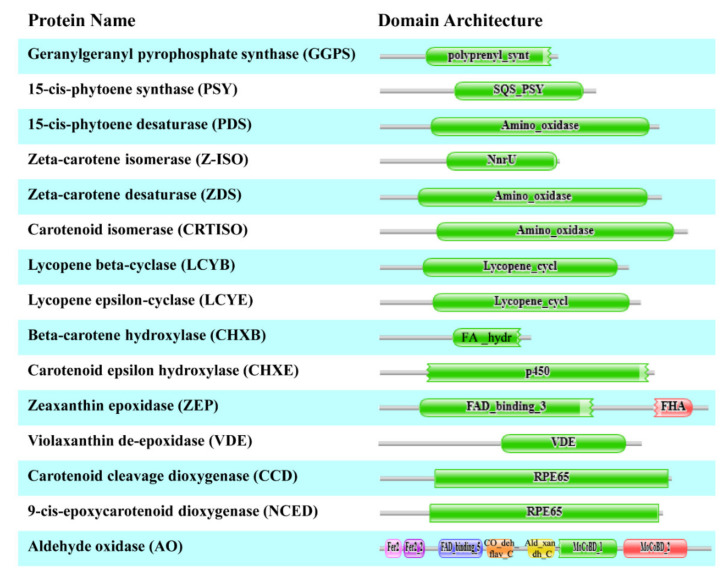
The conserved domain of 15 CP, XP, and AP genes in *H. moellendorffii*.

CDD search revealed that the CP, XP, and AP sequences showed high sequence homology with other higher plant species, including AA positions 92–359 for *HmGGPS*, 97–427 for *HmPSY*, 2–566 for *HmPDS*, 136–364 for *HmZ-ISO*, 2–562 for *HmZDS*, 108–602 for *HmCrtISO*, 60–505 for *HmLCYB*, 5–529 for *HmLCYE*, 32–305 for *HmCHXB*, 62–553 for *HmCHXE*, 1–662 for *HmZEP*, 251–503 for *HmVDE*, 5–589 for *HmCCD*, 11–574 for *HmNCED*, and 21–1365 for *HmAO*. The CDD of all 15 enzymes is shown in Figure 2. Similarly, the CCD homology search of the CP, XP, and AP gene sequences of other higher plants and marine green algae revealed similar conserved domains [18,19,41,45,46]. These results indicate that the *H. moellendorffii* CP, XP, and AP genes are highly conserved sequences.

Structural analyses of the AA sequences are necessary to identify the residues that play an important role in the catalytic function of the enzymes and are the main focus for the development of genetically engineered strains that can accumulate higher carotenoid content. Therefore, we identified the conserved motifs of the CP, XP, and AP genes using the MEME software, and the resulting sequence motif is shown in Figures S1 and S2. Motif analysis of *HmGGPS* and *HmPSY* enzymeS showed that they consist of conserved Asp-rich motifs (DDILD) and DELVD motifs, respectively (Figure S1). In *CHXE*, the fourth variable position of the pentapeptide consensus sequence (ATIXT) is a histidine residue in all plants and algae [39]. A similar result was obtained for the *HmCHXE* enzyme (Figure S1). For the *ZEP* enzyme, the conserved motif YFVXSD was found in a distinct organism, and the fourth position was replaced by aspartic acid, serine, valine, or threonine. However, in *HmZEP*, it is occupied by serine (Figure S1). The predicted dinucleotide FAD/NAD-binding domain (DX4GXG) was found in *LCYB*, whereas *HmLCYB* possesses a DLAVVGGG motif in their AA sequence. A recent study reported that they identified a few novel conserved motifs, such as RXXRHPQ, TPXINXGMV, and NNYGVW in *Z-ISO*, *CrtISO*, and *LCYE* sequences, in algae [39]. Similar conserved genes have been identified in *H. moellendorffii* (Figure S1). These conserved domains were present in various microalgae and plant AA sequences, indicating that these enzymes play vital roles in their biological function, substrate-binding activity, catalytic activity, and structural maintenance [39,47]. Hence, in the algae and plant kingdoms, the AA sequences of CP, XP, and AP are highly conserved [15,18,19,41,48]. 

### 2.3. Phylogenetic Tree and Percent Identity Matrix Analysis

Phylogenetic tree analysis between *H. moellendorffii* and other CP, XP, and AP genes was performed using the neighbor-joining method. The phylogenetic tree showed that it formed a cluster with other higher plants, whereas bacteria, chlorophytes, heterokonts, and dinoflagellates formed a separate cluster (Figure S2). Similar results have been obtained for several other plants [18,19,37,49,50,51]. The identity matrix results of the AA sequence showed that it shared the highest percentage of sequence identity with higher plants, especially *Daucus carota*. In addition, other species, such as bacteria, chlorophytes, dinoflagellates, and heterokonts showed a lower percentage of sequence identity with *H. moellendorffii* (Table S3). Previously, several higher plant studies, such as *C. majus*, *S. baicalensis*, *I. dentate*, and *N. officinale*, showed higher similarity with other higher plant AA sequences of CP, XP, and AP [18,19,28,29,37]. These results showed that the CP, XP, and AP genes in higher plants are highly conserved and may share the highest percentage of sequence identity with other higher plants.

**Figure 3:**
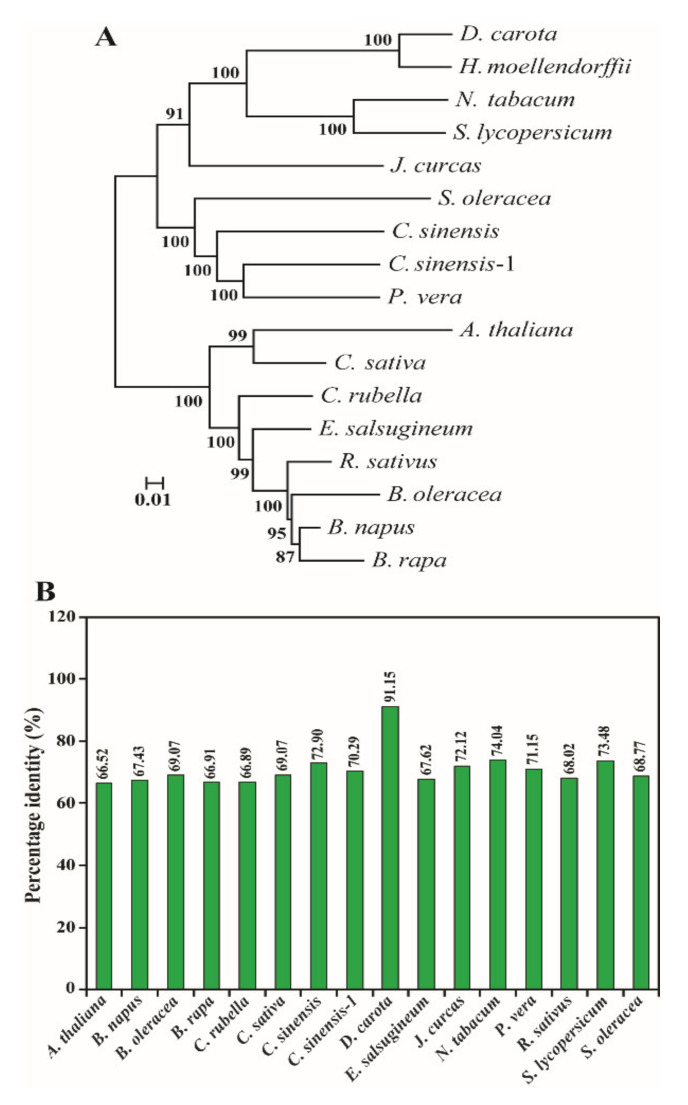
(**A**) Concatenated phylogenetic tree constructed based on the AA sequences of 15 CP, XP, and AP genes of *H. moellendorffii*. The tree was drawn by using the NJ method with the Poisson-correction distance. The number at each node represents bootstrap values. (**B**) Concatenated percentage identity (%) analysis of CP, XP, and AP amino acid sequences of *H. moellendorffii* and other higher plants. The sequences’ accession numbers are provided in Figure S2 and Table S3. *Arabidopsis thaliana*—*A. thaliana*, *Brassica napus*—*B. napus*, *Brassica oleracea*—*B. oleracea*, *Brassica rapa*—*B. rapa*, *Camelina sativa*—*C. sativa*, *Capsella rubella*—*C. rubella*, *Citrus sinensis*—*C. sinensis-1*, *Eutrema salsugineum*—*E. salsugineum*, *Jatropha curcas*—*J. curcas*, *Nicotiana tabacum*—*N. tabacum*, *Pistacia vera*—*P. vera*, *Raphanus sativus*—*R. sativus*, *Solanum lycopersicum*—*S. lycopersicum*, *Spinacia oleracea*—*S. oleracea*. Accession numbers of the amino acid sequences are shown in Table S4.

In addition, we constructed a concatenated phylogenetic tree based on the 15 AA sequences of the CP, XP, and AP genes. The phylogenetic results showed that these 16 higher plant species share a high bootstrap value with other higher plants, indicating that the CP, XP, and AP genes are highly conserved (Figure 3). In addition, these 15 CP, XP, and AP genes in *H. moellendorffii* share a similar putative conserved sequence; therefore, the function of these genes was also similar to those of other higher plants, especially *D. carota* (Figure 3). Similarly, the pairwise identity matrix of all AA sequences showed the highest percentage sequence identity with *D. carota* (Figure 3). This result was in agreement with a previous study, in which concatenated phylogenetic tree analysis and percentage identity matrix analysis of 11 *N. officinale* CP genes formed a cluster and the highest percentage identity matrix with *A. thaliana* [18]. These results indicate that all CP, XP, and AP genes share highly conserved sequences in higher plants.

### 2.4. Multiple Alignment and Protein Tertiary Structure Analysis of CP, XP, and AP Genes 

**Figure 4:**
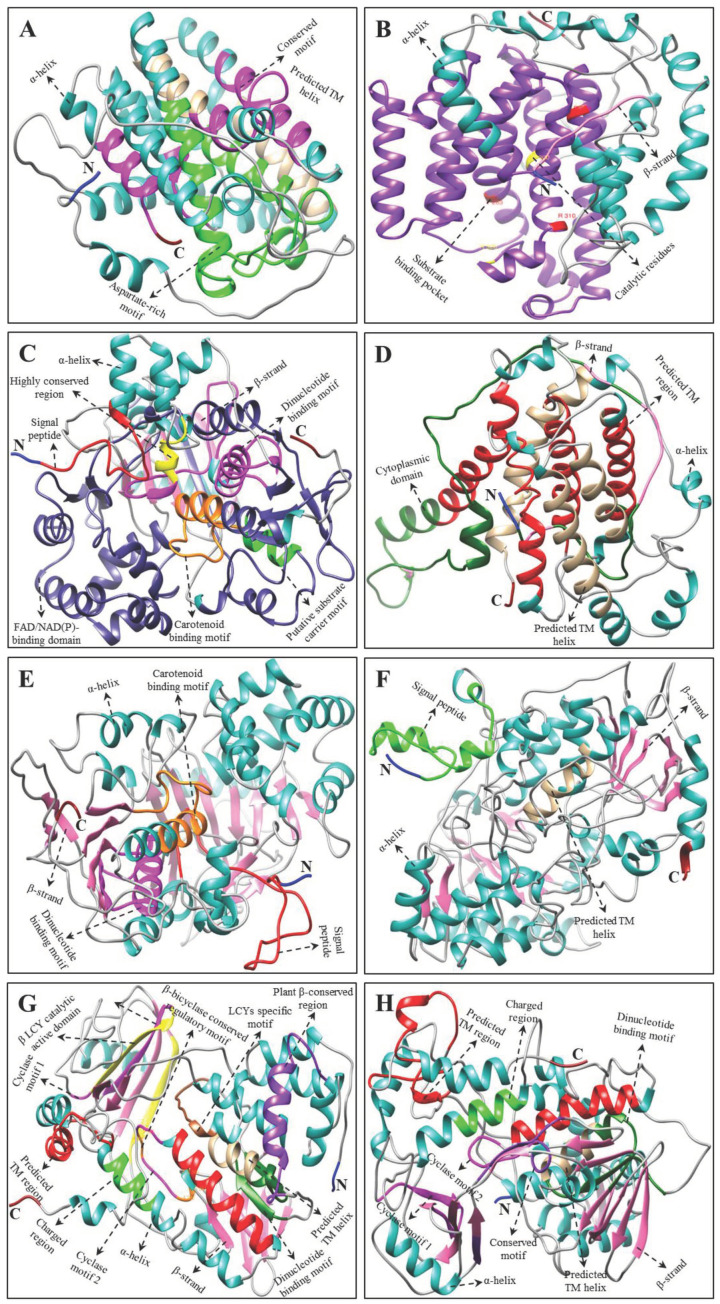
Tertiary structure of CP genes of *H. moellendorffii*. (**A**) *HmGGPS*, (B) *HmPSY*, (**C**) *HmPDS*, (**D**) *HmZ-ISO*, (**E**) *HmZDS*, (**F**) *HmCrtISO*, (**G**) *HmLCYB*, and (**H**) *HmLCYE* structures were created by using Chimera software (version 1.14) [62]. The amino (NH_2_), carboxyl (COOH) termini, α-helices, and β-strands are shown in blue, red, green, and pink, respectively. Multiple alignments of each CP gene are shown in Figure S3.

**Figure 5:**
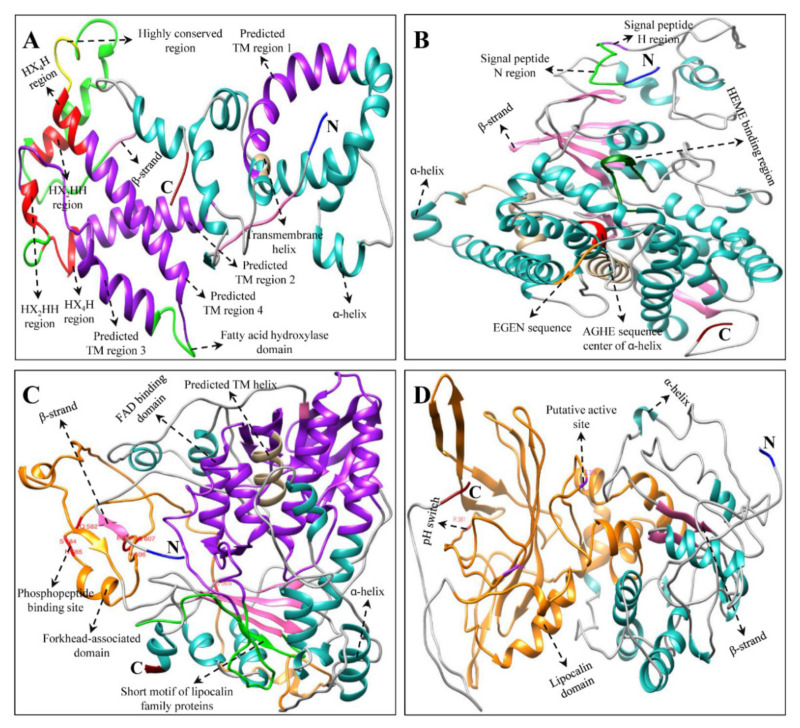
Tertiary structure of XP genes of *H. moellendorffii*. (**A**) *HmCHXB*, (**B**) *HmCHXE*, (**C**) *HmZEP*, and (**D**) *HmVDE* structures were created by using Chimera software (version 1.14) [62]. The amino (NH_2_), carboxyl (COOH) termini, α-helices, and β-strands are shown in blue, red, green, and pink, respectively. Multiple alignments of each XP gene are shown in Figure S3.

**Figure 6:**
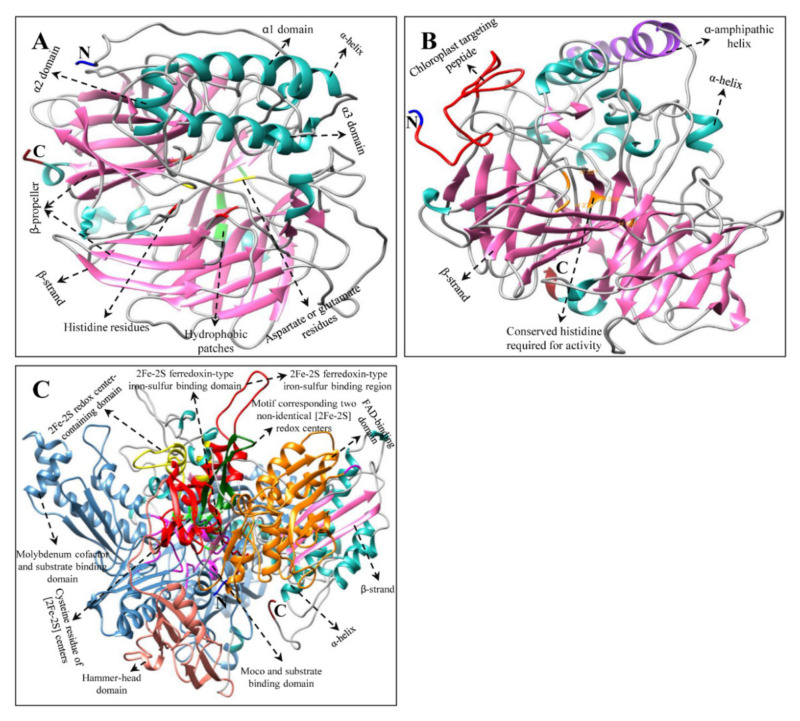
Tertiary structure of AP genes of H. moellendorffii. (**A**) HmCCD, (**B**) HmNCED, and (**C**) HmAO structures were created by using Chimera software (version 1.14) [62]. The amino (NH_2_), carboxyl (COOH) termini, α-helices, and β-strands are shown in blue, red, green, and pink, respectively. Multiple alignments of each AP gene are shown in Figure S3.

Multiple alignments and protein tertiary structure (3D) prediction of *H. moellendorffii* CP, XP, and AP proteins revealed conserved domains similar to those in other higher plants [18,19,28,32,51,52,53] and microalgae [45,54,55,56] (Figure 4, Figure 5, Figure 6 and Figure S3). Although there were few changes in AA, the protein’s function is primarily based on its 3D structure and stability [57]. The 3D structures of AA sequences exhibited similar substrate-binding pockets and configurations of α and β secondary structural elements as those of *C. reinhardtii*, *A. thaliana*, *C. majus*, *D. salina*, and *N. officinale* (data are not shown). However, slight structural differences were observed in the variable loop regions of the CP, XP, and AP protein models, which could be due to their comparatively low sequence similarities [58]. These results support the multiple alignment and percentage identity results of this study (Figure S3 and Table S3).

The predicted 3D structure of *H. moellendorffii* CP, XP, and AP genes possesses a hydrophobic substrate-binding pocket at the center, formed by the folding of α-helices and β-sheet strands. The substrate-binding pocket present in the structure was almost hidden within the core of the α-helices. Other domains, such as the aspartate-rich domain (ARD), dinucleotide-binding domain (DBD), and carotene-binding domain (CBD) were located near the cavity, which might play an important role in enzymatic activity [45]. Briefly, the key CP enzyme HmPSY possesses a conserved ARD and trans-isoprenyl diphosphate synthase motif in their sequence (Figure 4 and Figure S3). This result is in agreement with previous studies showing that these conserved domains are also present in higher plants (*C. majus*, *I. dentate*, *N. officinale*, and *S. baicalensis*) [18,19,28,31]. The second step in CP is the chemical reaction catalyzed by *HmPDS*, which possesses both CBD and DBD in its sequence. The third enzyme in the CP is *CmZDS* which shares structural features similar to those of *HmPDS*, consisting of a DBD and CBD at the N- and C-terminal regions, respectively. Similar structural features were found in the PDS and ZDS amino acid sequences of other higher plants (*Carica papaya*, *I. dentate*, *C. majus*, *S. baicalensis*, and *N. officinale*) and microalgae (*D. salina*) [18,19,28,31,56,59]. Both *HmLCYB* and *HmLCYE* contain a DBD in their AA sequences (Figure 4 and Figure S3). This conserved domain binds flavin adenine dinucleotides (FAD) and is found in all lycopene cyclases. Moreover, plant-type cyclases specifically consist of a plant β-conserved domain in its AA sequence, whereas it is absent in bacteria, and this β-conserved domain might play a significant role in the interaction between the components associated with membrane-bound enzymes and cyclases [60]. In addition, cyclase motif 1, cyclase motif 2, and charged regions are also present in lycopene cyclases, which are involved in the catalytic role of substrate-binding interactions [24]. Similar conserved motifs were identified in some higher plants (*C. majus*, *Arabidopsis, Capsicum annuum*, and *N. officinale*) and green microalgae (*Haematococcus pluvialis*) [18,19,24,60,61].

The most common genes in CP and XP were *HmCHXB* and *HmCHXE*, respectively. *HmCHXB* possesses four histidine residues in its structure, which may be helpful for the binding of Fe^2+^ ions during hydroxylation [28,63]. The *VDE* gene in *C. sativus* and *C. majus* consists of three conserved domains: lipocalin, Glu-rich, and Cys-rich motifs [19,51]. The central lipocalin domain serves as an attachment site for the hydrophobic V substrate [64]. In addition, a high number of glutamate residues and a transit peptide that targets the protein to chloroplasts were found in the C- and N- terminal regions, respectively [64,65]. *HmVDE* consisted of similar conserved protein domains in their AA sequences in this study (Figure 5 and Figure S3). *HmZEP* possessed various phosphopeptide-binding sites, two short motifs of lipocalin family proteins, one forkhead-associated binding motif, and one FAD-binding motif in its AA sequence. Similar motifs have been identified in the AA sequences of some higher plants (*C. majus*, *I. dentate*, *S. baicalensis*, and *N. officinale*) [18,19,28,31].

Among XP genes, *HmCCD* and *HmNCED* consisted of four highly conserved histidine residues in their AA sequences (Figure 6 and Figure S3), similar to the CCD and NCED structures of *C. majus*, *N. officinale*, and citrus plants [18,19,66]. Previous studies indicated that these highly conserved histidine moieties aid in coordinating the Fe^2+^ cofactor required for the activity or the aspartate or glutamate moieties that help fix the histidine positions [67,68,69]. In *Pisum sativum* and *C. majus*, the *AO* gene consists of an FAD-binding motif, a molybdenum cofactor (Moco) binding motif, and a consensus sequence for two iron-sulfur centers [19,70]. In this study, *HmAO* also consisted of similar conserved domains (Figure 6 and Figure S3). Multiple alignments and 3D structural analysis indicated that most of the *H. moellendorffii* CP, XP, and AP genes have highly conserved sequences. Therefore, these genes are closely related to those from other higher plants and algae. However, further studies are needed to identify the functions of the *H. moellendorffii* CP, XP, and AP genes.

### 2.5. In Silico Subcellular Localization Prediction of CP, XP, and AP Genes

The subcellular localization prediction of *H. moellendorffii* CP, XP, and AP amino acid sequences were analyzed using free online programs such as CELLO2GO, DeepLOC-1.0, Plant-PLoc, WoLF PSORT, and TargetP 1.1. All CP, XP, and AP genes were directed to the chloroplast, whereas some of these genes were also targeted to different organelles, such as the cytoplasm, cell membrane, endoplasmic reticulum, nucleus, mitochondrion, thylakoid membrane, and plasma membrane (Table 1). Several previous studies in higher plants (*A. thaliana*, *C. majus*, transgenic *Ipomoea batatas*, and *N. officinale*) and microalgae have reported that most of the CP, XP, and AP genes are targeted to the chloroplast [18,19,46,71,72], confirming that methylerythritol 4-phosphate/1-deoxy-D-xylulose 5-phosphate (MEP/DOXP) and CP occur in the chloroplast/plastid of higher plants and algae [39,73]. Therefore, this result confirmed that all CP, XP, and AP proteins in *H. moellendorffii* share highly conserved sequences with those in higher plants and algae; therefore, the prediction of subcellular localization analysis of all these AA sequences showed similar results.

### 2.6. Differential Expression of CP, XP, and AP Genes in Different Parts of H. moellendorffii

**Figure 7:**
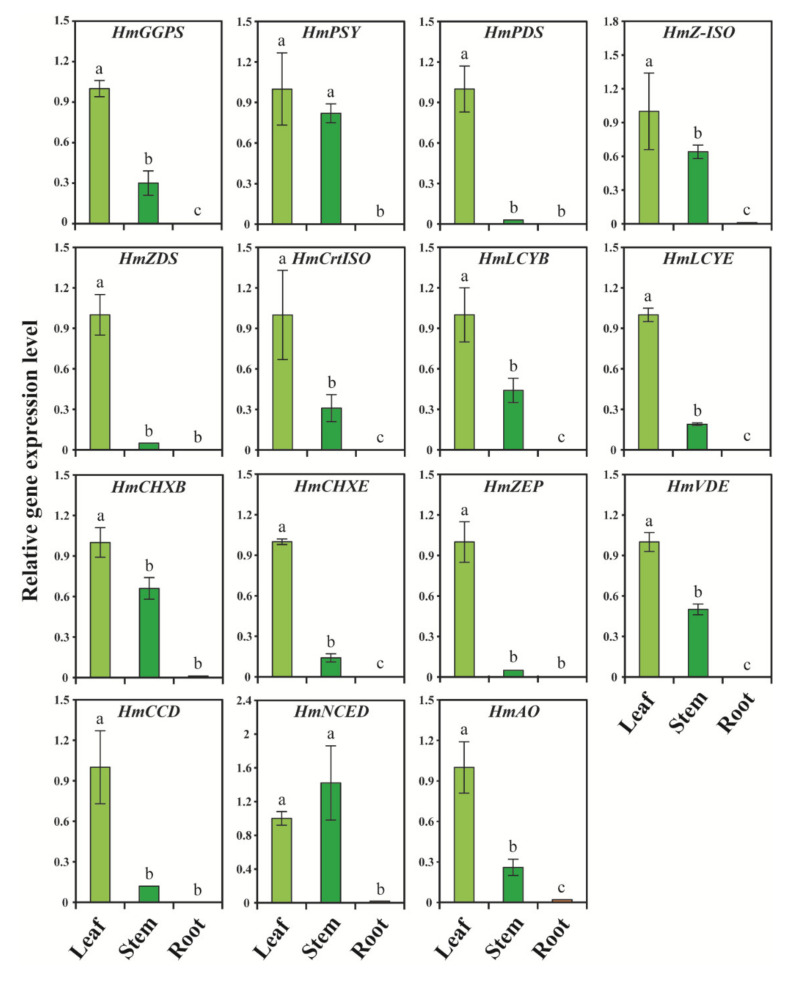
Relative gene expression level of 15 CP, XP, and AP genes of *H. moellendorffii*. Expression levels of CP, XP, and AP genes were analyzed in different parts such as leaf, stem, and root of *H. moellendorffii* using qRT-PCR. Transcriptional levels in leaves were set as a control (the value of 1) to calculate the relative gene expression in other tissues. The relative gene expression levels of CP, XP, and AP genes were normalized to *α-tubulin* as a reference gene. Results are given as the means of three replicates ± standard deviation. Different alphabetical letters a–c denote significant differences (*p* < 0.05).

The results of qRT–PCR showed that the CP, XP, and AP genes were highly expressed in the leaves of *H. moellendorffii*, whereas none of these genes were significantly expressed in the roots. The highest gene expression was achieved in *HmCCD* and *HmNCED*, whereas the lowest expression level was achieved with *HmPDS* (Figure 7). All the CP genes (*HmPSY*, *HmPDS*, *HmZ-ISO*, *HmCrtISO*, *HmLCYB*, and *HmLCYE*) were significantly expressed in leaves. The expression level of *HmPSY* was elevated in leaves, which was 980.39- and 1.21-times higher than that in the roots and stems, respectively. In addition, most XP genes, such as *HmCHXB*, *HmCHXE*, *HmZEP*, and *HmVDE*, were highly upregulated in leaves compared to stems and roots. In addition, the *HmVDE* expression level was elevated in leaves, which was 333.33- and 2.0-times higher than that in roots and stems, respectively. The expression level of *HmCCD* was significantly higher in the leaves than in the stems. In contrast, *HmNCED* had the highest expression in the stem, which was 71.0- and 1.42-times higher than that in roots and leaves, respectively (Figure 7). *HmAO* was significantly expressed in leaves, which was 3.85- and 50-times higher than that in the stem and root, respectively. Previous studies reported that the CP, XP, and AP genes were significantly expressed in the leaves and flowers of plants, such as *C. majus* [19], *Brassica rapa* [74], and *N. officinale* [18], when compared with different parts of the plants. In *D. carota*, the expression level of CP, XP, and AP genes was significantly expressed in the leaves compared to that of the roots [75]. A similar result was obtained in this study, in that most of the CP, XP, and AP genes were highly expressed in leaves. This indicates that most gene sequences are highly conserved, and all these genes have roles similar to their orthologs in other species. In *Arabidopsis,* it has been reported that genes involved in CP, such as *AtPSY*, *AtPDS*, *AtZDS*, and *AtZEP* play important roles [76]. PSY is the rate-limiting enzyme for CP, resulting in the highest accumulation of individual and total carotenoids in plants [9]. The results showed that *HmPSY* was involved in controlling carotenoid flux in the leaves of *H. moellendorffii*. In addition, enzymes such as PSY, PDS, and ZDS genes are facilitated by light, which results in carotenoid accumulation in carrot leaves [75]. A similar result was obtained in the present study due to the exposure of leaves and stems to light; the expression levels of *HmPSY*, *HmPDS*, and *HmZDS* were higher, which led to the highest accumulation of carotenoids in leaves and stems. In addition, the roots showed the lowest expression level and carotenoid accumulation when compared to leaves and stems due to the lack of exposure to light. Moreover, β-carotene degradation genes such as *HmZEP*, *HmVDE*, *HmAO*, and *HmCCD4* were highly expressed in leaves compared to roots. A previous study reported that the leaf-specific enzyme CCD4 is an important enzyme involved in apocarotenoid glycoside formation [77]. In this study, the expression of *HmCCD4* in *H. moellendorffii* leaves might be due to the association with apocarotenoid glycoside formation in the leaves; however, the exact functions need to be studied in the future. This study showed that, except for *HmNCED*, all other genes were highly expressed in leaves. This expression analysis of CP, XP, and AP genes will provide immense knowledge in the future to perform molecular studies on *H. moellendorffii* to enhance carotenoid accumulation through genetic engineering approaches.

### 2.7. Carotenoid and Xanthophyll Content in Different Organs of H. moellendorffii

**Figure 8:**
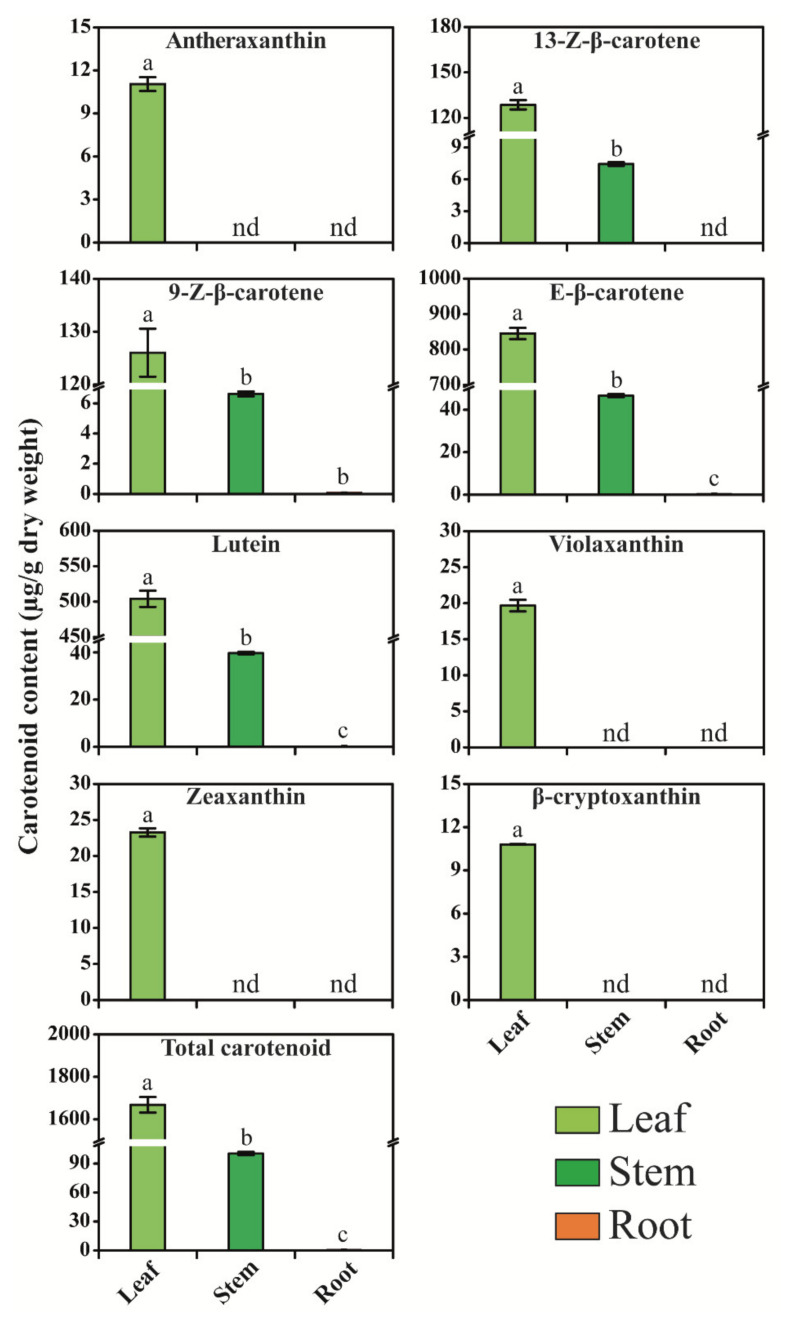
Carotenoid and xanthophyll content in the different parts of *H. moellendorffii*. For extraction of carotenoids, samples were collected from 3-month-old plants. Results are given as the means of three replicates ± standard deviation. Different alphabetical letters a–c denote significant differences (*p* < 0.05). “nd” for not detected.

Eight different carotenoids were identified in different organs of *H. moellendorffii* using high-performance liquid chromatography (HPLC) (Figure 8). The total carotenoid level varied between 0.73 and 1668.20 μg/g of dry weight (DW). Among the different organs, the highest total carotenoid level was found in leaves (1668.20 μg/g DW), 2285.21- and 16.61-times higher than that in the roots and stems, respectively. All eight individual carotenoids were significantly higher in leaves (Figure 8). Specifically, E-β-carotene, lutein, 9Z-β-carotene, and 13Z-β-carotene levels were considerably higher in leaves than in other organs.

**Figure 9:**
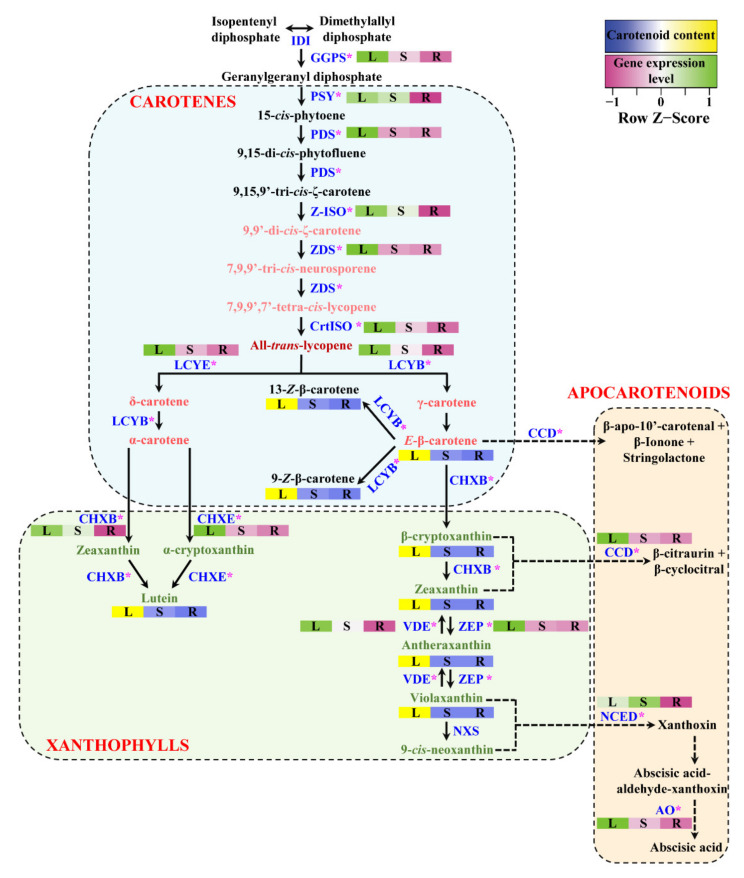
Summary of CP, XP, and AP genes related to carotenoid, xanthophyll, and apocarotenoid biosynthesis in *H. moellendorffii*. Each different-colored square box denotes the relative gene expression level (pink to green) and carotenoid and xanthophyll accumulation level (blue to yellow) in different organs of *H. moellendorffii*. The scale bar represents the transformed average value of CP, XP, and AP gene expression levels and metabolites. L—Leaf; S—Stem; R—Root. Asterisks represent the gene used for gene expression.

Among the carotenoids, E-β-carotene, lutein, and 9Z-β-carotene were detected in all the plant organs. Specifically, the E-β-carotene level was higher in leaves, which was 2112.0- and 18.11-times higher than that in roots and stems, respectively. The lutein content was also higher in leaves than in stems and roots. In addition, 9Z-β-carotene accumulation was the highest in leaves, and it was 1400.11- and 19.03-times higher than that in roots and stems, respectively. Antheraxanthin, violaxanthin, zeaxanthin, and β-cryptoxanthin were detected only in leaves. The 13-Z-β-carotene was considerably high in leaves (128.70 μg/g DW), followed by that in stems (7.45 μg/g DW), and it was not detected in stem roots. Violaxanthin, antheraxanthin, zeaxanthin, and β-cryptoxanthin levels were considerably higher in the leaves than in the stems and roots. Among the specific carotenoids, β-cryptoxanthin, antheraxanthin, and violaxanthin showed the lowest accumulation in different organs of *H. moellendorffii* (Figure 8). These findings are in agreement with those of previous studies of *Allium sativum* [78], *C. majus* [19,79], *N. officinale* [18], *B. rapa* [30], *D. carota* [18], and *M. charantia* [78,80,81]. In this study, the CP, AP, and XP genes were highly expressed in the leaves, which led to the highest accumulation of carotenoids in the leaves (Figure 9). A previous study reported that in red and yellow carrots, the accumulation of main carotenoids and total carotenoids might be due to the participation of transcriptional levels of genes that lead to CP [82]. In addition, CHXB overexpression regulated α-carotene and total carotenoid levels in orange carrots [83]. The carotenoid genomic database of *H. moellendorffii* may be a useful tool for researchers to create a model to enhance the accumulation of carotenoids in different plant parts.

## 3. Materials and Methods

### 3.1. Plant Growth

*Heracleum moellendorffii* seeds were obtained from Aram Seed Co., Ltd., Seoul, Korea, and grown in the greenhouse of Chungnam National University, Daejeon, Korea. The seeds were sown in a plastic pot containing perlite and grown in a greenhouse for three months. Plants were watered every second day. Various plant parts such as the leaves, stems, and roots were harvested after three months (April 2021–July 2021), frozen in liquid nitrogen, and immediately stored at −80 °C. Each sample was harvested in triplicate.

### 3.2. Mining and Sequence Analysis of CP, XP, and AP Genes

CP, XP, and AP gene sequences were retrieved from *H. moellendorffii* transcriptome data acquired in our laboratory. The identified sequences were analyzed using Basic Local Alignment Search Tool (BLAST). Consequently, the gene sequences were also examined using Conserved Domain Database (CCD) [84] and PFAM [85] on the National Center for Biotechnology Information (NCBI) GenBank database to identify the signature motifs present in the gene sequences. The theoretical pI (isoelectric point)/molecular weight (MW), aliphatic index, instability index, and grand average of hydropathicity (GRAVY) values were estimated using the ExPASy ProtParam platform [86]. The in silico subcellular locations of the CP, XP, and AP proteins were predicted using free online software, such as TargetP 1.1 [87], CELLO2GO [88], WoLF PSORT [89], DeepLOC-1.0 [90], and Plant-PLoc [91]. Signal peptide, secondary structure analyses, and the disordered region of proteins were determined using the SignalP 4.0 server [92], SOPMA program [93], and PSIPRED tool [94], respectively. The total number of transmembrane helices present in the CP, XP, and AP genes was predicted using the TMHMM-2.0 server [95]. Moreover, the internal repeats and N-glycosylation sites in the CP, XP, and AP genes were identified using the RADAR tool [96] and NetNglyc 1.0 server [97], respectively.

### 3.3. Multiple Alignment and Tertiary Structural Analysis of CP, XP, and AP Genes

The BioEdit 7.2.5 program [98] was used to perform the multiple alignment of the identified sequences. Tertiary structural analysis of the CP, XP, and AP protein sequences was performed using the Chimera 1.14 software [62]. The MEME tool [99] was used to detect the conserved motifs.

### 3.4. Phylogenetic Tree Construction and Percent Identity Matrix

For phylogenetic analysis, all the identified sequences were created using MEGA 7.0 [100]. A phylogenetic tree was constructed using the neighbor-joining (NJ) algorithm with 1000 bootstrap replicates [101,102] The percent identities of the CP, XP, and AP amino acid sequences were estimated using the free online software Clustal Omega [103], and pairwise sequence alignment [104] was used to calculate the identities of the sequences.

### 3.5. RNA Isolation and cDNA Synthesis

The leaves, stems, and roots of *H. moellendorffii* were used for total RNA extraction. Each sample was crushed into a fine powder with liquid nitrogen using a mortar and pestle. Each sample (100 mg) was aliquoted into new microcentrifuge tubes. The Plant Total RNA Mini Kit (Geneaid, Taiwan) was used for extraction according to the manufacturer’s instructions. RNA quality and concentration were estimated using a NanoVue Plus spectrophotometer (GE Healthcare Life Sciences, MA, USA). cDNA was then synthesized using a ReverTra Ace-α-kit (Toyobo Co. Ltd., Osaka, Japan), according to the manufacturer’s instructions. The cDNA was diluted 20-fold with sterile RNase-free water for analysis.

### 3.6. Genes Expression Analysis

The primers, listed in Table S5, were designed using Gene Runner 5.0 software (http://www.generunner.net accessed on 22 October 2021). The house-keeping gene *α-tubulin* was used as an internal control to calculate the relative gene expression levels. The quantitative reverse transcription–polymerase chain reaction (qRT–PCR) was performed according to the protocol described by Sathasivam et al. [18,19]. All reactions were performed in triplicate.

### 3.7. Extractions of Carotenoid and HPLC Analysis

Extraction and analysis of carotenoids were performed according to the procedure described by Sathasivam et al. [18,19], with slight modifications. Finely ground samples (300 mg) were collected, and 3 mL of 0.1% ascorbic acid (*w*/*v*) in ethanol was added. The samples were immediately placed on ice for 5 min to terminate the reaction. After termination, 0.05 mL of β-apo-8′-carotenal (internal standard) and 1.5 mL of ice-cold water were added to the mixture. Subsequently, 1.5 mL hexane was added, and the sample was centrifuged at 4 °C for 5 min at 12,000 rpm. After centrifugation, the mixture was dried under nitrogen and liquefied in 250 µL of CH_2_Cl_2_/MeOH (50:50 *v*/*v*). These extracts were filter-sterilized in glass screw-cap vials. The carotenoids were analyzed by using an Agilent Technologies 1100 Series HPLC system (Palo Alto, CA, USA) that consisted of a C30 YMC column (250 × 4.6 mm, 3 m; Waters Corporation, Milford, MA, USA), a degasser, and a PDA detector. The mobile phase and gradient program were as follows: solvent A, methanol/water (92:8 *v*/*v*) with 10 mM ammonium acetate; solvent B, methyl tert-butylether; 0 min, 90% A; 20 min, 83% A; 29 min, 75% A; 35 min, 30% A; 40 min, 30% A; 42 min, 25% A; 45 min, 90% A; 55 min, 90% A (Total 50 min). The separation of carotenoid compounds was detected at 450 nm at 40 °C with the flow rate of 1.0 mL/min, and the injection volume was 10 µL. The carotenoid content of each part was quantified with reference to a corresponding calibration curve.

### 3.8. Statistical Analysis

The results are expressed as the mean ± standard deviation of three replicates. Data were evaluated by one-way analysis of variance (ANOVA) with Duncan’s multiple range tests (DMRT) to compare means. *p*-values < 0.05, were measured as statistically significant using SPSS version 26 (SPSS, Chicago, IL, USA).

## 4. Conclusions

In conclusion, we have studied the genes involved in CP, XP, and AP because of their importance in plant metabolism and function, such as physiology, ecology, development, and evolution. Recently, researchers have focused on carotenoid accumulation at various regulatory levels. Therefore, mining of CP, XP, and AP genes from the available transcriptomic data, in silico characterization of the mined genes using free online bioinformatics software, and understanding the expression levels of CP, XP, and AP genes will help in finding the underlying connection between the transcriptomic and metabolomic profiles. In addition, mining of CP, XP, and AP genes and analyzing their 3D structure will be useful for researchers in gene manipulation, potential gene engineering, and transformation of multiple CP, XP, and AP genes into the desired host to enhance carotenoid biosynthesis and enrich the desired or novel carotenoid products in stable crops. These structural analyses, gene expression profiles of CP, XP, and AP genes, and carotenoid accumulation in different parts of the plant will increase our knowledge regarding the accumulation of carotenoids at the molecular level in *H. moellendorffii*. This information might be a useful resource for altering genes through genetic engineering to enhance the carotenoid content of *H. moellendorffii*. However, further studies are needed to identify new *H. moellendorffii* CP, XP, and AP genes using a genome-wide approach which will aid in identifying several homologous genes, gene families, and alleles.

## Figures and Tables

**Table 1:** The subcellular-localization predictions of *H. moellendorffii* CP, XP, and AP Genes.

Gene Names	CELLO2GO	DeepLOC-1.0	Plant-PLoc	TargetP	WoLF PSORT	Consensus Prediction
*HmGGPS*	CP	PLS	CP/PLS	Other	CP	CP/PLS/Other
*HmPSY*	CP	PLS	CP	Other	CYT/NUC	CP/CYT/NUC/PLS/Other
*HmPDS*	CP	PLS/CM	CP	CP	CP	CP/CM/PLS
*HmZ-ISO*	PM	PLS/CM	CM	CP	PM	CP/CM/PLS/PM
*HmZDS*	MC	PLS/CM	CP	CP	CP	CP/CM/PLS/MC
*HmCrtISO*	PM	PLS/CM	CP	CP	CP	CP/CM/PLS/PM
*HmLCYB*	CP	PLS/CM	CP	Other	CP	CP/CM/PLS/Other
*HmLCYE*	PM	PLS/CM	CP	Other	PM	CP/CM/PLS/Other
*HmCHXB*	CP	PLS/CM	VAC	CP	PM	CP/CM/PLS/PM/VAC
*HmCHXE*	CP	PLS	ER	CP	CP	CP/ER/PLS
*HmZEP*	CP	PLS/CM	CP	CP	CP	CP/CM/PLS
*HmVDE*	NUC	PLS/CM	CP/NUC	CP	CP	CP/CM/NUC/PLS
*HmCCD*	CP	PLS	CYT	CP	CP	CP/CYT/PLS
*HmNCED*	CYT	PLS	CYT	CP	MC	CP/CYT/MC/PLS
*HmAO*	PM	CYT	CP	Other	PM	CP/CYT/PM/Other

Note: CP—Chloroplast; CYT—Cytoplasm; CME—Cell Membrane; ER—Endoplasmic reticulum; MC—Mitochondria; NUC—Nucleus; PM—Plasma membrane; PLS—Plastid; VAC—Vacuole.

## Data Availability

Data reported are available in the Supplementary Materials.

## Supplementary Materials

The following supporting information can be downloaded at: https://www.mdpi.com/article/10.3390/ijms23094845/s1.

## References

[1] Kang L., Yu Y., Zhou S.-D., He X.-J. (2019). Sequence and phylogenetic analysis of complete plastid genome of a medicinal plant *Heracleum moellendorffii*. Mitochondrial DNA Part B.

[2] Bang J.-E., Choi H.-Y., Kim S.-I. (2009). Anti-oxidative activity and chemical composition of various *Heracleum moellendorffii* Hance extracts. Korean J. Food Preserv..

[3] Alam M.B., Seo B.-J., Zhao P., Lee S.-H. (2016). Anti-melanogenic activities of heracleum moellendorffii via ERK1/2-mediated MITF downregulation. Int. J. Mol. Sci..

[4] Geum N.G., Son H.J., Yeo J.H., Yu J.H., Choi M.Y., Lee J.W., Baek J.K., Jeong J.B. (2021). Anti-obesity activity of *Heracleum moellendorffii* root extracts in 3T3-L1 adipocytes. Food Sci. Nutr..

[5] Park H.-J., Nugroho A., Jung B.-R., Won Y.-H., Jung Y.-J., Kim W.-B., Choi J.-S. (2010). Isolation and quantitative analysis of flavonoids with peroxynitritescavenging effect from the young leaves of *Heracleum moellendorffii*. Korean J. Plant Res..

[6] Kim H.N., Kim J.D., Yeo J.H., Son H.-J., Park S.B., Park G.H., Eo H.J., Jeong J.B. (2019). *Heracleum moellendorffii* roots inhibit the production of pro-inflammatory mediators through the inhibition of NF-κB and MAPK signaling, and activation of ROS/Nrf2/HO-1 signaling in LPS-stimulated RAW264. 7 cells. BMC Complement. Altern. Med..

[7] Son H.J., Eo H.J., Park G.H., Jeong J.B. (2021). *Heracleum moellendorffii* root extracts exert immunostimulatory activity through TLR2/4-dependent MAPK activation in mouse macrophages, RAW264. 7 cells. Food Sci. Nutr..

[8] Chu S.S., Cao J., Liu Q.Z., Du S.S., Deng Z.W., Liu Z.L. (2012). Chemical composition and insecticidal activity of Heracleum moellendorffii Hance essential oil. Chemija.

[9] Sathasivam R., Radhakrishnan R., Kim J.K., Park S.U. (2020). An update on biosynthesis and regulation of carotenoids in plants. S. Afr. J. Bot..

[10] Sathasivam R., Kermanee P., Roytrakul S., Juntawong N. (2012). Isolation and molecular identification of β-carotene producing strains of *Dunaliella salina* and *Dunaliella bardawil* from salt soil samples by using species-specific primers and internal transcribed spacer (ITS) primers. Afr. J. Biotechnol..

[11] Sathasivam R., Pongpadung P., Praiboon J., Chirapart A., Trakulnaleamsai S., Roytrakul S., Juntawong N. (2018). Optimizing NaCl and KNO_3_ concentrations for high β-carotene production in photobioreactor by *Dunaliella salina* KU11 isolated from saline soil sample. Chiang Mai J. Sci.

[12] Sathasivam R., Praiboon J., Chirapart A., Trakulnaleamsai S., Kermanee P., Roytrakul S., Juntawong N. (2014). Screening, phenotypic and genotypic identification of β-carotene producing strains of *Dunaliella salina* from Thailand. Indian J. Geo-Mar. Sci..

[13] Sathasivam R., Radhakrishnan R., Hashem A., Abd_Allah E.F. (2019). Microalgae metabolites: A rich source for food and medicine. Saudi J. Biol. Sci..

[14] Sathasivam R., Ki J.-S. (2018). A review of the biological activities of microalgal carotenoids and their potential use in healthcare and cosmetic industries. Mar. Drugs.

[15] Sathasivam R., Ki J.-S. (2019). Differential transcriptional responses of carotenoid biosynthesis genes in the marine green alga *Tetraselmis suecica* exposed to redox and non-redox active metals. Mol. Biol. Rep..

[16] Hughes D.A. (1999). Effects of carotenoids on human immune function. Proc. Nutr. Soc..

[17] Mihaylova D., Vrancheva R., Petkova N., Ognyanov M., Desseva I., Ivanov I., Popova M., Popova A. (2018). Carotenoids, tocopherols, organic acids, charbohydrate and mineral content in different medicinal plant extracts. Z. Naturforsch. C.

[18] Sathasivam R., Bong S.J., Park C.H., Kim J.H., Kim J.K., Park S.U. (2022). Identification, characterization, and expression analysis of carotenoid biosynthesis genes and carotenoid accumulation in watercress (*Nasturtium officinale* R. Br.). ACS Omega.

[19] Sathasivam R., Yeo H.J., Park C.H., Choi M., Kwon H., Sim J.E., Park S.U., Kim J.K. (2021). Molecular characterization, expression analysis of carotenoid, xanthophyll, apocarotenoid pathway genes, and carotenoid and xanthophyll accumulation in *Chelidonium majus* L. Plants.

[20] Hulkko L.S., Chaturvedi T., Thomsen M.H. (2022). Extraction and quantification of chlorophylls, carotenoids, phenolic compounds, and vitamins from halophyte biomasses. Appl. Sci..

[21] Horváth G., Molnár P., Farkas A., Szabó L.G., Turcsi E., Deli J. (2010). Separation and identification of carotenoids in flowers of *Chelidonium majus* L. and inflorescences of *Solidago canadensis* L. Chromatographia.

[22] Molnár P., Kawase M., Motohashi N., Motohashi N. (2005). Isolation, crystallization and handling of carotenoids and (E/Z)-isomerization of carotenoids. Functional Polyphenols and Carotenes with Antioxidative Action.

[23] Stanley L., Yuan Y.-W. (2019). Transcriptional regulation of carotenoid biosynthesis in plants: So many regulators, so little consensus. Front. Plant Sci..

[24] Cunningham F.X., Pogson B., Sun Z.R., McDonald K.A., DellaPenna D., Gantt E. (1996). Functional analysis of the beta and epsilon lycopene cyclase enzymes of *Arabidopsis* reveals a mechanism for control of cyclic carotenoid formation. Plant Cell.

[25] Devitt L.C., Fanning K., Dietzgen R.G., Holton T.A. (2010). Isolation and functional characterization of a lycopene beta-cyclase gene that controls fruit colour of papaya (*Carica papaya* L.). J. Exp. Bot..

[26] Kato M., Ikoma Y., Matsumoto H., Sugiura M., Hyodo H., Yano M. (2004). Accumulation of carotenoids and expression of carotenoid biosynthetic genes during maturation in citrus fruit. Plant Physiol..

[27] Li C., Ji J., Wang G., Li Z.D., Wang Y.R., Fan Y.J. (2020). Over-expression of *LcPDS*, *LcZDS*, and *LcCRTISO*, genes from wolfberry for carotenoid biosynthesis, enhanced carotenoid accumulation, and salt tolerance in tobacco. Front. Plant Sci..

[28] Reddy C.S., Lee S.H., Yoon J.S., Kim J.K., Lee S.W., Hur M., Koo S.C., Kim M.R., Lee W.M., Jang J.K. (2017). Molecular cloning and characterization of carotenoid pathway genes and carotenoid content in *Ixeris dentata* var. *albiflora*. Molecules.

[29] Tan B.C., Joseph L.M., Deng W.T., Liu L.J., Li Q.B., Cline K., McCarty D.R. (2003). Molecular characterization of the *Arabidopsis* 9-cis epoxycarotenoid dioxygenase gene family. Plant J..

[30] Tuan P.A., Kim J.K., Lee J., Park W.T., Kwon D.Y., Kim Y.B., Kim H.H., Kim H.R., Park S.U. (2012). Analysis of carotenoid accumulation and expression of carotenoid biosynthesis genes in different organs of Chinese cabbage (*Brassica rapa* Subsp *pekinensis*). EXCLI J..

[31] Tuan P.A., Kim Y.B., Kim J.K., Arasu M.V., Al-Dhabi N.A., Park S.U. (2015). Molecular characterization of carotenoid biosynthetic genes and carotenoid accumulation in *Scutellaria baicalensis* Georgi. EXCLI J..

[32] Zhu H.S., Chen M.D., Wen Q.F., Li Y.P. (2015). Isolation and characterization of the carotenoid biosynthetic genes LCYB, LCYE and CHXB from strawberry and their relation to carotenoid accumulation. Sci. Hortic.-Amst..

[33] Jeon J., Bong S.J., Park J.S., Park Y.K., Arasu M.V., Al-Dhabi N.A., Park S.U. (2017). De novo transcriptome analysis and glucosinolate profiling in watercress (*Nasturtium officinale* R. Br.). BMC Genom..

[34] Voutsina N., Payne A.C., Hancock R.D., Clarkson G.J., Rothwell S.D., Chapman M.A., Taylor G. (2016). Characterization of the watercress (*Nasturtium officinale* R. Br.; Brassicaceae) transcriptome using RNASeq and identification of candidate genes for important phytonutrient traits linked to human health. BMC Genom..

[35] Bong S.J., Jeon J., Park Y.J., Kim J.K., Park S.U. (2020). Identification and analysis of phenylpropanoid biosynthetic genes and phenylpropanoid accumulation in watercress (*Nasturtium officinale* R. Br.). 3 Biotech.

[36] Lopez-Emparan A., Quezada-Martinez D., Zuniga-Bustos M., Cifuentes V., Iniguez-Luy F., Federico M.L. (2014). Functional analysis of the *Brassica napus* L. phytoene synthase (PSY) gene family. PLoS ONE.

[37] Tuan P.A., Kim J.K., Lee S., Chae S.C., Park S.U. (2013). Molecular characterization of carotenoid cleavage dioxygenases and the effect of gibberellin, abscisic acid, and sodium chloride on the expression of genes involved in the carotenoid biosynthetic aathway and carotenoid accumulation in the callus of *Scutellaria baicalensis* Georgi. J. Agr. Food Chem..

[38] Idicula-Thomas S., Balaji P.V. (2005). Understanding the relationship between the primary structure of proteins and its propensity to be soluble on overexpression in Escherichia coli. Protein Sci..

[39] Narang P.K., Dey J., Mahapatra S.R., Roy R., Kushwaha G.S., Misra N., Suar M., Raina V. (2022). Genome-based identification and comparative analysis of enzymes for carotenoid biosynthesis in microalgae. World J. Microbiol. Biotechnol..

[40] Zhou X.-T., Jia L.-D., Duan M.-Z., Chen X., Qiao C.-L., Ma J.-Q., Zhang C., Jing F.-Y., Zhang S.-S., Yang B. (2020). Genome-wide identification and expression profiling of the carotenoid cleavage dioxygenase (CCD) gene family in *Brassica napus* L. PLoS ONE.

[41] Kaur N., Pandey A., Kumar P., Pandey P., Kesarwani A.K., Mantri S.S., Awasthi P., Tiwari S. (2017). Regulation of banana phytoene synthase (MaPSY) expression, characterization and their modulation under various abiotic stress conditions. Front. Plant Sci..

[42] Flowerika, Alok A., Kumar J., Thakur N., Pandey A., Pandey A.K., Upadhyay S.K., Tiwari S. (2016). Characterization and expression analysis of phytoene synthase from bread wheat (*Triticum aestivum* L.). PLoS ONE.

[43] Ye Z.-W., Liu G.-N., Jiang J.-G. (2011). Structural and phylogenetic analysis of a novel ζ-carotene desaturase from *Dunaliella bardawil*, a unicellular alga that accumulates large amounts of β-carotene. Limnol. Oceanogr..

[44] Smith L.J., Fiebig K.M., Schwalbe H., Dobson C.M. (1996). The concept of a random coil: Residual structure in peptides and denatured proteins. Fold. Des..

[45] Zhe Q.L., Zheng J.L., Liu J.H. (2020). Transcription activation of beta-carotene biosynthetic genes at the initial stage of stresses as an indicator of the increased beta-carotene accumulation in isolated *Dunaliella* salina strain GY-H13. Aquat. Toxicol..

[46] Han Y., Zheng Q.S., Wei Y.P., Chen J., Liu R., Wan H.J. (2015). In silico identification and analysis of phytoene synthase genes in plants. Genet. Mol. Res..

[47] Milton R.D., Minteer S.D. (2017). Direct enzymatic bioelectrocatalysis: Differentiating between myth and reality. J. R. Soc. Interface.

[48] Engprasert S., Taura F., Kawamukai M., Shoyama Y. (2004). Molecular cloning and functional expression of geranylgeranyl pyrophosphate synthase from *Coleus forskohliiBriq*. BMC Plant Biol..

[49] Chen Y., Li F.Q., Wurtzel E.T. (2010). Isolation and characterization of the Z-ISO gene encoding a missing component of carotenoid biosynthesis in plants. Plant Physiol..

[50] Li Z.D., Wu G.X., Ji J., Wang G., Tian X.W., Gao H.L. (2015). Cloning and expression of a zeta-carotene desaturase gene from *Lycium chinense*. J. Genet..

[51] Li X., Zhao W., Sun X., Huang H., Kong L., Niu D., Sui X., Zhang Z. (2013). Molecular cloning and characterization of violaxanthin de-epoxidase (CsVDE) in cucumber. PLoS ONE.

[52] Ahrazem O., Rubio-Moraga A., Berman J., Capell T., Christou P., Zhu C.F., Gomez-Gomez L. (2016). The carotenoid cleavage dioxygenase CCD2 catalysing the synthesis of crocetin in spring crocuses and saffron is a plastidial enzyme. New Phytol..

[53] Gao Z., Liu Q., Zheng B., Chen Y. (2013). Molecular characterization and primary functional analysis of PeVDE, a violaxanthin de-epoxidase gene from bamboo (*Phyllostachys edulis*). Plant Cell Rep..

[54] Cui H.L., Wang Y.C., Qin S. (2011). Molecular evolution of lycopene cyclases involved in the formation of carotenoids in eukaryotic algae. Plant Mol. Biol. Rep..

[55] Zhu Y.H., Jiang J.G., Chen Q. (2008). Characterization of cDNA of lycopene beta-cyclase responsible for a high level of beta-carotene accumulation in *Dunaliella salina*. Biochem. Cell Biol..

[56] Zhu Y.H., Jiang J.G., Yan Y., Chen X.W. (2005). Isolation and characterization of phytoene desaturase cDNA involved in the beta-carotene biosynthetic pathway in *Dunaliella salina*. J. Agr. Food Chem..

[57] DePristo M.A., Weinreich D.M., Hartl D.L. (2005). Missense meanderings in sequence space: A biophysical view of protein evolution. Nat. Rev. Genet..

[58] Garg R., Jhanwar S., Tyagi A.K., Jain M. (2010). Genome-wide survey and expression analysis suggest diverse roles of glutaredoxin gene family members during development and response to various stimuli in rice. DNA Res..

[59] Yan P., Gao X.Z., Shen W.T., Zhou P. (2011). Cloning and expression analysis of phytoene desaturase and zeta-carotene desaturase genes in *Carica papaya*. Mol. Biol. Rep..

[60] Hugueney P., Badillo A., Chen H.C., Klein A., Hirschberg J., Camara B., Kuntz M. (1995). Metabolism of cyclic carotenoids—A model for the alteration of this biosynthetic pathway in *Capsicum annuum* chromoplasts. Plant J..

[61] Lao Y.M., Jin H., Zhou J., Zhang H.J., Cai Z.H. (2017). Functional characterization of a missing branch component in *Haematococcus pluvialis* for control of algal carotenoid biosynthesis. Front. Plant Sci..

[62] Pettersen E.F., Goddard T.D., Huang C.C., Couch G.S., Greenblatt D.M., Meng E.C., Ferrin T.E. (2004). UCSF chimera—A visualization system for exploratory research and analysis. J. Comput. Chem..

[63] Bouvier F., Keller Y., D’Harlingue A., Camara B. (1998). Xanthophyll biosynthesis: Molecular and functional characterization of carotenoid hydroxylases from pepper fruits (*Capsicum annuum* L.). BBA-Lipid Lipid Met..

[64] Charron J.-B.F., Ouellet F., Pelletier M., Danyluk J., Chauve C., Sarhan F. (2005). Identification, expression, and evolutionary analyses of plant lipocalins. Plant Physiol..

[65] Arnoux P., Morosinotto T., Saga G., Bassi R., Pignol D. (2009). A structural basis for the pH-dependent xanthophyll cycle in *Arabidopsis thaliana*. Plant Cell.

[66] Rodrigo M.J., Alquezar B., Alos E., Medina V., Carmona L., Bruno M., Al-Babili S., Zacarias L. (2013). A novel carotenoid cleavage activity involved in the biosynthesis of citrus fruit-specific apocarotenoid pigments. J. Exp. Bot..

[67] Huang F.C., Molnar P., Schwab W. (2009). Cloning and functional characterization of carotenoid cleavage dioxygenase 4 genes. J. Exp. Bot..

[68] Messing S.A.J., Gabelli S.B., Echeverria I., Vogel J.T., Guan J.C., Tan B.C., Klee H.J., McCarty D.R., Amzel L.M. (2010). Structural insights into maize viviparous14, a key enzyme in the biosynthesis of the phytohormone abscisic acid. Plant Cell.

[69] Schwartz S.H., Tan B.C., Gage D.A., Zeevaart J.A.D., McCarty D.R. (1997). Specific oxidative cleavage of carotenoids by VP14 of maize. Science.

[70] Zdunek-Zastocka E. (2008). Molecular cloning, characterization and expression analysis of three aldehyde oxidase genes from *Pisum sativum* L. Plant Physiol. Biochem..

[71] Joyard J., Ferro M., Masselon C., Seigneurin-Berny D., Salvi D., Garin J., Rolland N. (2009). Chloroplast proteomics and the compartmentation of plastidial isoprenoid biosynthetic pathways. Mol. Plant.

[72] Kang C., Zhai H., Xue L.Y., Zhao N., He S.Z., Liu Q.C. (2018). A lycopene beta-cyclase gene, IbLCYB2, enhances carotenoid contents and abiotic stress tolerance in transgenic sweetpotato. Plant Sci..

[73] Sun X.-M., Ren L.-J., Zhao Q.-Y., Ji X.-J., Huang H. (2018). Microalgae for the production of lipid and carotenoids: A review with focus on stress regulation and adaptation. Biotechnol. Biofuels.

[74] Li P.R., Zhang S.J., Zhang S.F., Li F., Zhang H., Cheng F., Wu J., Wang X.W., Sun R.F. (2015). Carotenoid biosynthetic genes in *Brassica rapa*: Comparative genomic analysis, phylogenetic analysis, and expression profiling. BMC Genom..

[75] Ma J., Li J., Xu Z., Wang F., Xiong A. (2018). Transcriptome profiling of genes involving in carotenoid biosynthesis and accumulation between leaf and root of carrot (*Daucus carota* L.). Acta Biochim. Biophys. Sin..

[76] Ruiz-Sola M.A., Rodriguez-Concepcion M. (2012). Carotenoid biosynthesis in Arabidopsis: A colorful pathway. Arab. Book.

[77] Lätari K., Wüst F., Hübner M., Schaub P., Beisel K.G., Matsubara S., Beyer P., Welsch R. (2015). Tissue-specific apocarotenoid glycosylation contributes to carotenoid homeostasis in *Arabidopsis* leaves. Plant Physiol..

[78] Tuan P.A., Kim J.K., Kim H.H., Lee S.Y., Park N.I., Park S.U. (2011). Carotenoid accumulation and characterization of cDNAs encoding phytoene synthase and phytoene desaturase in Garlic (*Allium sativum*). J. Agr. Food Chem..

[79] Khodabande Z., Jafarian V., Sariri R. (2017). Antioxidant activity of *Chelidonium majus* extract at phenological stages. Appl. Biol. Chem..

[80] Cuong D.M., Arasu M.V., Jeon J., Park Y.J., Kwon S.J., Al-Dhabi N.A., Park S.U. (2017). Medically important carotenoids from *Momordica charantia* and their gene expressions in different organs. Saudi J. Biol. Sci..

[81] Tuan P.A., Kim J.K., Park N.I., Lee S.Y., Park S.U. (2011). Carotenoid content and expression of phytoene synthase and phytoene desaturase genes in bitter melon (*Momordica charantia*). Food Chem..

[82] Clotault J., Peltier D., Berruyer R., Thomas M., Briard M., Geoffriau E. (2008). Expression of carotenoid biosynthesis genes during carrot root development. J. Exp. Bot..

[83] Arango J., Jourdan M., Geoffriau E., Beyer P., Welsch R. (2014). Carotene hydroxylase activity determines the levels of both α-carotene and total carotenoids in orange carrots. Plant Cell.

[84] Lu S., Wang J., Chitsaz F., Derbyshire M.K., Geer R.C., Gonzales N.R., Gwadz M., Hurwitz D.I., Marchler G.H., Song J.S. (2020). CDD/SPARCLE: The conserved domain database in 2020. Nucleic Acids Res..

[85] Mistry J., Chuguransky S., Williams L., Qureshi M., Salazar G.A., Sonnhammer E.L., Tosatto S.C., Paladin L., Raj S., Richardson L.J. (2021). Pfam: The protein families database in 2021. Nucleic Acids Res..

[86] Gasteiger E., Gattiker A., Hoogland C., Ivanyi I., Appel R.D., Bairoch A. (2003). ExPASy: The proteomics server for in-depth protein knowledge and analysis. Nucleic Acids Res..

[87] Emanuelsson O., Nielsen H., Brunak S., Von Heijne G. (2000). Predicting subcellular localization of proteins based on their N-terminal amino acid sequence. J. Mol. Biol..

[88] Yu C.S., Chen Y.C., Lu C.H., Hwang J.K. (2006). Prediction of protein subcellular localization. Proteins.

[89] Horton P., Park K.-J., Obayashi T., Fujita N., Harada H., Adams-Collier C., Nakai K. (2007). WoLF PSORT: Protein localization predictor. Nucleic Acids Res..

[90] Almagro Armenteros J.J., Sønderby C.K., Sønderby S.K., Nielsen H., Winther O. (2017). DeepLoc: Prediction of protein subcellular localization using deep learning. Bioinform.

[91] Chou K.-C., Shen H.-B. (2008). Cell-PLoc: A package of Web servers for predicting subcellular localization of proteins in various organisms. Nat. Protoc..

[92] Petersen T.N., Brunak S., Von Heijne G., Nielsen H. (2011). SignalP 4.0: Discriminating signal peptides from transmembrane regions. Nat. Methods.

[93] Geourjon C., Deleage G. (1995). SOPMA: Significant improvements in protein secondary structure prediction by consensus prediction from multiple alignments. Bioinform.

[94] McGuffin L.J., Bryson K., Jones D.T. (2000). The PSIPRED protein structure prediction server. Bioinform.

[95] Krogh A., Larsson B., Von Heijne G., Sonnhammer E.L. (2001). Predicting transmembrane protein topology with a hidden Markov model: Application to complete genomes. J. Mol. Biol..

[96] Heger A., Holm L. (2000). Rapid automatic detection and alignment of repeats in protein sequences. Proteins.

[97] Gupta R., Jung E., Brunak S. (2004). Prediction of N-Glycosylation Sites in Human Proteins. NetNGlyc 1.0. NetNGlyc Website. http://www.cbs.dtu.dk/services/NetNGlyc/.

[98] Hall T.A. (1999). BioEdit: A user-friendly biological sequence alignment editor and analysis program for Windows 95/98/NT. Nucleic Acids Symp. Ser..

[99] Bailey T.L., Boden M., Buske F.A., Frith M., Grant C.E., Clementi L., Ren J.Y., Li W.W., Noble W.S. (2009). MEME SUITE: Tools for motif discovery and searching. Nucleic Acids Res..

[100] Kumar S., Stecher G., Tamura K. (2016). MEGA7: Molecular evolutionary genetics analysis version 7.0 for bigger datasets. Mol. Biol. Evol..

[101] Saitou N., Nei M. (1987). The neighbor-joining method—A new method for reconstructing phylogenetic trees. Mol. Biol. Evol..

[102] Felsenstein J. (1985). Confidence limits on phylogenies: An approach using the bootstrap. Evolution.

[103] Sievers F., Wilm A., Dineen D., Gibson T.J., Karplus K., Li W., Lopez R., McWilliam H., Remmert M., Söding J. (2011). Fast, scalable generation of high-quality protein multiple sequence alignments using Clustal Omega. Mol. Syst. Biol..

[104] Madeira F., Park Y.M., Lee J., Buso N., Gur T., Madhusoodanan N., Basutkar P., Tivey A.R.N., Potter S.C., Finn R.D. (2019). The EMBL-EBI search and sequence analysis tools APIs in 2019. Nucleic Acids Res..

