# Supplementary material for: Identification, In Silico Characterization, and Differential Expression Profiles of Carotenoid, Xanthophyll, Apocarotenoid Biosynthetic Pathways Genes, and Analysis of Carotenoid and Xanthophyll Accumulation in Heracleum moellendorffii Hance
Source: Int J Mol Sci. 2022 Apr 27;23(9):4845. doi: 10.3390/ijms23094845 (PMC9099461; doi:10.3390/ijms23094845)
Supplement: Supplementary file 1 [file ijms-23-04845-s001.zip › ijms-1690256-supplementary.pdf]

Supplementary Materials

```
-136 CCATTTAAGTGTCCATTTCTCAACCCTCCATTAAACACACATATGTAAACACTCTTCAAT
     TGAACACTCTTCAATTAAACACTCTTCAAACATTATCCCAGGAATCGCAAAAACTCCGAC
   1 ATGAAGTGTTTGAACATGTTGAATCCATGGCTACTTCAAACTAACTCAATCTTCAATCAC
      M  K  C  L  N  M  L  N  P  W  L  L  Q  T  N  S  I  F  N  H
  61 TCAAACACATCAAACACTTTCATTACAACTTACAACAATCAATCAAAACAACTCAAACCC
      S  N  T  S  N  T  F  I  T  T  Y  N  N  Q  S  K  Q  L  K  P
 121 ACTTTCTCAAATCCCAAATCAATCCGACCCATTTCATCTGTTCTCACTAATCAACAAACC
      T  F  S  N  P  K  S  I  R  P  I  S  S  V  L  T  N  Q  Q  T
 181 AAACAAACCCCAACTCAAACATTCGATTTCAATTCTTACATGATTGAAAAAGCTGTTTTA
      K  Q  T  P  T  Q  T  F  D  F  N  S  Y  M  I  E  K  A  V  L
 241 GTTAACACTGCACTAAACGACGCCGTTTTAGTACAAGAACCTCCCATGATTCACGAAGCC
      V  N  T  A  L  N  D  A  V  L  V  Q  E  P  P  M  I  H  E  A
 301 ATGCGGTACTCTCTTCTCGCCGGAGGTAAACGTGTCCGTCCAATGCTTTGTATCGCCGCC
      M  R  Y  S  L  L  A  G  G  K  R  V  R  P  M  L  C  I  A  A
 361 TGTGAGCTTGTTGGTGGGCACCAATCATCTGCCATACCTGCTGCTTGTGCTGTCGAAATG
      C  E  L  V  G  G  H  Q  S  S  A  I  P  A  A  C  A  V  E  M
 421 ATACACACCATGTCATTAATCCATGATGACTTGCCTTGTATGGATAACGATGATCTTCGT
      I  H  T  M  S  L  I  H  D  D  L  P  C  M  D  N  D  D  L  R
 481 CGTGGAAAGCCAACCAATCACAAGGTCTATGGTGAGGACGTGGCAGTCCTGGCCGGAGAT
      R  G  K  P  T  N  H  K  V  Y  G  E  D  V  A  V  L  A  G  D
 541 TCATTGCTGGCATTTGCATTTTATCATATTGCAACATCGACAAAAGGCGTAACGCCTAAT
      S  L  L  A  F  A  F  Y  H  I  A  T  S  T  K  G  V  T  P  N
 601 AGAATTATACAAGCAGTTGGTGAACTTGCCAAGTCTATTGGGACACAAGGGTTAGTAGCT
      R  I  I  Q  A  V  G  E  L  A  K  S  I  G  T  Q  G  L  V  A
 661 GGGCAAGTGGTGGATATTTTATCAACCGGGGATTCGTCCGTTGGATTGGAACAACTTGAA
      G  Q  V  V  D  I  L  S  T  G  D  S  S  V  G  L  E  Q  L  E
 721 TTTATACACATTCATAAGACCGCTGTATTGCTAGAAGCATCTGTGGTTTTAGGAGCTATT
      F  I  H  I  H  K  T  A  V  L  L  E  A  S  V  V  L  G  A  I
 781 TTAGGGGGTGGAGATGATGAAGAAGTAGAGAAACTAAGAAAATTTGCGAGGTGTGTGGGA
      L  G  G  G  D  D  E  E  V  E  K  L  R  K  F  A  R  C  V  G
 841 CTGTTGTTTCAAGTAGTTGATGACATTCTAGATGTTACGAAATCGTCTCAAGAATTGGGT
      L  L  F  Q  V  V [D  D  I  L  D] V  T  K  S  S  Q  E  L  G
 901 AAAACTGCAGGGAAGGATTTGATGGTTGATAAGTCTACGTATCCGAAGCTACTAGGGTTG
      K  T  A  G  K  D  L  M  V  D  K  S  T  Y  P  K  L  L  G  L
 961 GAGAAATCGAGAGAGTTTGCTGAGAAGTTGAATGAAGAAGCTAAAGATCAGCTGGCGGGG
      E  K  S  R  E  F  A  E  K  L  N  E  E  A  K  D  Q  L  A  G
1021 TTTGATGAGGGTAAGGCAGCTCCTTTAGTTGCTTTGGCTAATTACATTGCTTATAGGAAT
      F  D  E  G  K  A  A  P  L  V  A  L  A  N  Y  I  A  Y  R  N
1081 AATTAGTTGGAAATGTTACTACATGTTGGATTGCAAGTTTAGTTATTAGTTTGGTGGTGC
      N  *
     AAGAAATGCACATTCTTCTTCTTGATTCAGCTTTTGTATTTAATTTATGTTTACTGATTC
     AATTTTTGTTCATACTTGTATAAAATTCCTGGTTCTGTAAATTTATGCAAGAGAACTCAT +495
```

Figure S1A. The nucleotide sequence and deduced amino acid sequences of HmGGPS. An asterisk (*) represents a stop codon.

```
-121 AATCTTGAAGCTTCTTGGGTGTGAATGGTTGAGGCTTCTCCACTAAGGGCTGACAAGGAA
     GAAAGAAACTAAGAGGAGTTTATATTAAATTGAAGTTTTTGGCTAGTACGAACTCCAAC
   1 ATGTCAGTTGCTATGTGGTGGATTGGTACTCCGAATCTTGAGGTATCCAATTGCTTCGGG
      M  S  V  A  M  W  W  I  G  T  P  N  L  E  V  S  N  C  F  G
  61 TACTTGGAGACTGTCCGAGAGGGAACCCGAGTATTGGATTCGTCGAGGTTGGTTTTCCGG
      Y  L  E  T  V  R  E  G  T  R  V  L  D  S  S  R  L  V  F  R
 121 GACAGAATTATGACGTGTGGTGGTAGACTCAAGAAGGGTAAGCTGCGGAAGTGGAGTTGT
      D  R  I  M  T  C  G  G  R  L  K  K  G  K  L  R  K  W  S  C
 181 AGTTCTTCTAATGCTGAATTTAGCTACTCGTGTTTGGGCAGTCCTGAATTAGAGAGCGGA
      S  S  S  N  A  E  F  S  Y  S  C  L  G  S  P  E  L  E  S  G
 241 AGTATATTTCCTGTGCATTCAAGTATGGTAGTGAGTGCGGATGGAGATATGGCAGTGTCA
      S  I  F  P  V  H  S  S  M  V  V  S  A  D  G  D  M  A  V  S
 301 TCAGAGCAAAAAGTTTATGATGTGGTTCTTAAACAAGCGGCGTTGGTTAAGAAACAGTTC
      S  E  Q  K  V  Y  D  V  V  L  K  Q  A  A  L  V  K  K  Q  F
 361 AGATCTGACGAGGAATTAGAGGTTAAGCCAGAAATTATTCTTCCAGGGACTCTGAGCTTG
      R  S  D  E  E  L  E  V  K  P  E  I  I  L  P  G  T  L  S  L
 421 TTAAGCGAAGCTTATGATCGATGCGGTGAAGTATGTGCCGAATATGCCAAAACATTTTAC
      L  S  E  A  Y  D  R  C  G  E  V  C  A  E  Y  A  K  T  F  Y
 481 TTGGGAACACTACTGATGACCCCAGAGAGGCGGAGAGCTATCTGGGCAATATATGTATGG
      L  G  T  L  L  M  T  P  E  R  R  R  A  I  W  A  I  Y  V  W
 541 TGTAGAAGAACGGATGAACTGGTAGATGGACCTAATGCGTCACATATAACTCCTTCAGCT
      C  R  R  T [D  E  L  V  D] G  P  N  A  S  H  I  T  P  S  A
 601 TTGGATAGGTGGGAGTCGAGATTAGAAGATCTTTACAAGGGGCGTCCATTTGATATGCTC
      L  D  R  W  E  S  R  L  E  D  L  Y  K  G  R  P  F  D  M  L
 661 GATGCTGCTTTATCAGATACAGTTATTAAGTTTCCTGTTGACATTCAGCCATTCAAAGAC
      D  A  A  L  S  D  T  V  I  K  F  P  V  D  I  Q  P  F  K  D
 721 ATGATTGAAGGAATGAGGATGGACCTTAAGAAGTCAAGATACAAGAACTTTGATGAGCTA
      M  I  E  G  M  R  M  D  L  K  K  S  R  Y  K  N  F  D  E  L
 781 TATCTTTATTGCTATTATGTTGCTGGTACTGTTGGATTGATGAGTGTTCCAATTATGGGC
      Y  L  Y  C  Y  Y  V  A  G  T  V  G  L  M  S  V  P  I  M  G
 841 ATTGCACCTGATTCACAGGCAACAACTGAGAGTGTTTATAATGCTGCCTTGGCGTTAGGG
      I  A  P  D  S  Q  A  T  T  E  S  V  Y  N  A  A  L  A  L  G
 901 CTCGCTAATCAATTGACTAACATACTCAGAGATGTTGGAGAAGATGCTAGAAGAGGAAGG
      L  A  N  Q  L  T  N  I  L  R  D  V  G  E  D  A  R  R  G  R
 961 GTTTATCTACCACAGGATGAACTGGTGCAAGCAGGGCTTTCTGATGAAGATATATTTGCA
      V  Y  L  P  Q  D  E  L  V  Q  A  G  L  S  D  E  D  I  F  A
1021 GGGAAGGTTACAGATAAATGGAGGAATTTCATGAAGAAGCAAATTAAGAGGGCGAGGATG
      G  K  V  T  D  K  W  R  N  F  M  K  K  Q  I  K  R  A  R  M
1081 TTTTTCAATGAAGCAGAAAAAGGTGTAAGAGAGCTGAGCCCAGCTAGTAGATGGCCGGTA
      F  F  N  E  A  E  K  G  V  R  E  L  S  P  A  S  R  W  P  V
1141 TGGGCATCACTGCTGTTGTATCGTCAAATACTAGATGAAATTGAAGCCAACGACTACAAT
      W  A  S  L  L  L  Y  R  Q  I  L  D  E  I  E  A  N  D  Y  N
1201 AATTTTACGAAGAGAGCTTACGTGAGCAAACCCAAGAAAATACTTTCTTTGCCAGTTGCA
      N  F  T  K  R  A  Y  V  S  K  P  K  K  I  L  S  L  P  V  A
1261 TATGCAAAGGCTCTTGCCCCCACAGCAAGAACAGGTTCTACATTGTTGAAGACATGAATG
      Y  A  K  A  L  A  P  T  A  R  T  G  S  T  L  L  K  T  *
     GTATCATGTAATATAAATTCAAATCAAATAGAAAGATTCATATAATGTAGAAAAAGAAGC
     AAGGCAGATATTTTGTACATTAAATTAAATGGTCCATGTAAAAAATATTAGTTTATAGTG
     AAATAATTATGTA +168
```

Figure S1B. The nucleotide sequence and deduced amino acid sequences of HmPSY. An asterisk (*) represents a stop codon.

```
-98   TTCTTCCTCCATTAGTATATTAATTAATTAATATCTAACAACTAGCAGGCCTGCTCACAA
      ATAAAACCCACTCAATTTTCTTGTTGAGCTGCAACAATACTCATACAGGGAAGCATCAAT
   1  ATGTCTCAATTTGGACATGTATCTTCTGCTGTCATGACACATAACAACAATTTCAATCTA
       M  S  Q  F  G  H  V  S  S  A  V  M  T  H  N  N  N  F  N  L
  61  CTCAACAACAAATACAAATTTCAGTCCCCCCTTTCATTTTCTGCTACTATCTCCTCCACC
       L  N  N  K  Y  K  F  Q  S  P  L  S  F  S  A  T  I  S  S  T
 121  ACACTTAAAAAACATTCCGCTTCTAATTTTACGCCGCGCACACGCCTTCTCAAGGTGTCT
       T  L  K  K  H  S  A  S  N  F  T  P  R  T  R  L  L  K  V  S
 181  TGTGTGGACTATCCCAGGCCAGATATCGACAACACTCTTCCTTTTTTAGAAGCTGCCTAC
       C  V  D  Y  P  R  P  D  I  D  N  T  L  P  F  L  E  A  A  Y
 241  TTATCTTCATTCTTTTCGACTGCTTCTCGTCCGTCTAAGCCGTTAAATGTTGTCATTGCT
       L  S  S  F  F  S  T  A  S  R  P  S  K  P  L  N  V  V  I  A
 301  GGTGCAGGTTTGGCTGGATTATCTACTGCAAAATATTTGGCAGATGCTGGTCACAAGCCC
       G  A  G  L  A  G  L  S  T  A  K  Y  L  A  D  A  G  H  K  P
 361  ATCTTGTTGGAAGCAAGAGATGTTCTTGGTGGAAAGGTGGCTGCTTGGAAAGATGATGAT
       I  L  L  E  A  R  D  V  L  G  G  K  V  A  A  W  K  D  D  D
 421  GGAGACTGGTACGAGACTGGATTACACATTTTCTTTGGGGCTTACCCAAATGTTCAGAAC
       G  D  W  Y  E  T  G  L  H  I  F  F  G  A  Y  P  N  V  Q  N
 481  CTGTTTGGAGAACTAGGAATTGACAACCGATTGCAATGGAAGGAGCATTCTATGATATTC
       L  F  G  E  L  G  I  D  N  R  L  Q  W  K  E  H  S  M  I  F
 541  GCTATGCCTAACAAGCCTGGGGAATTTAGTAGATTTGATTTTCCGGAAATTCTACCTGCA
       A  M  P  N  K  P  G  E  F  S  R  F  D  F  P  E  I  L  P  A
 601  CCACTAAATGGAATATGGGCTATCTTGAAGAATAATGAAATGCTAACATGGCCTGAGAAG
       P  L  N  G  I  W  A  I  L  K  N  N  E  M  L  T  W  P  E  K
 661  GTCAAGTTTGCATTGGGCCTCTTGCCAGCGATAATTGGTGGACAAGCCTACGTGGAGGCG
       V  K  F  A  L  G  L  L  P  A  I  I  G  G  Q  A  Y  V  E  A
 721  CAAGATGGTCTAAGTGTCCAAGATTGGATGAGAAAGCAAGGGATACCTGATCGGGTTACA
       Q  D  G  L  S  V  Q  D  W  M  R  K  Q  G  I  P  D  R  V  T
 781  ACTGAGGTTTTTGTTGCCATGTCAAAGTCGCTAAACTTTATCAATCCAGACGAACTTTCG
       T  E  V  F  V  A  M  S  K  S  L  N  F  I  N  P  D  E  L  S
 841  ATGCAATGTGTATTGATTGCTTTGAACCGATTTCTTCAGGAGAAGCATGGTTCAAAGATG
       M  Q  C  V  L  I  A  L  N  R  F  L  Q  E  K  H  G  S  K  M
 901  GCTTTCTTGGATGGAAGTCCTCCAGAAAGACTTTGCATGCCAATAGTTGATCACATACAG
       A  F  L  D  G  S  P  P  E  R  L  C  M  P  I  V  D  H  I  Q
 961  TCACTGGGTGGTGAAGTTCATCTCAATTCACGAGTACAGAAGATCTCTTTAAATAAAGAT
       S  L  G  G  E  V  H  L  N  S  R  V  Q  K  I  S  L  N  K  D
1021  CATACTGTTAAGAGTCTATTACTAACCAATGGGAAGGTTATTGAAGCAGATGCATATGTA
       H  T  V  K  S  L  L  L  T  N  G  K  V  I  E  A  D  A  Y  V
1081  ATTGCTGCTCCAGTTGATATCCTAAAGCTACTTGTGCCTGAAGAGTGGAGAGAGATACCA
       I  A  A  P  V  D  I  L  K  L  L  V  P  E  E  W  R  E  I  P
1141  TACTTCAAGAAGTTGGATAAACTAGTTGGAGTTCCAGTAATCAATGTTCACATATGGTTC
       Y  F  K  K  L  D  K  L  V  G  V  P  V  I  N  V  H  I  W  F
1201  GACAGGAAACTGAAGAACACATACGATCATCTACTTTTCAGCAGAAGCTCACTTCTTAGC
       D  R  K  L  K  N  T  Y  D  H  L  L  F  S  R  S  S  L  L  S
1261  GTATATGCTGATATGTCTGTAACTTGTAAGGAATATTACGACCCAAATAAGTCGATGCTG
       V  Y  A  D  M  S  V  T  C  K  E  Y  Y  D  P  N  K  S  M  L
1321  GAGTTGGTTTTTGCACCTGCAGAAGAATGGATTTCACGCAGTGACTCTGACATTATTGAT
       E  L  V  F  A  P  A  E  E  W  I  S  R  S  D  S  D  I  I  D
1381  GCAACAATGAATGAACTGGCCAGACTATTTCCTGATGAGATTGCTGCTGATCAGAGCAAA
       A  T  M  N  E  L  A  R  L  F  P  D  E  I  A  A  D  Q  S  K
1441  GCAAAAATATTGAAGTACCATGTTGTTAAAACACCAAGGTCTGTTTATAAAACCATACCA
       A  K  I  L  K  Y  H  V  V  K  T  P  R  S  V  Y  K  T  I  P
1501  GACTGTGAACCCTGCCGTCCTTTGCAAAAATCTCCTATAGAAGGATTTTATTTAGCTGGT
       D  C  E  P  C  R  P  L  Q  K  S  P  I  E  G  F  Y  L  A  G
1561  GATTACACAAAACAGAGGTATCTGGCCTCTATGGAGGGTGCTGTCCTCTCGGGAAAGCTT
       D  Y  T  K  Q  R  Y  L  A  S  M  E  G  A  V  L  S  G  K  L
1621  TGTGCTCAAGCTATACTGCAGGACCATGAGTCGCTGCTTTCCCGCAGGAAGAATGTGCTG
       C  A  Q  A  I  L  Q  D  H  E  S  L  L  S  R  R  K  N  V  L
1681  GCTGAGGCAAGTGTTGTCTAATTATAGCAATAAAAGGTGATGCAAGTATTCTGAGCAATC
       A  E  A  S  V  V  *
      AGTAAGAGGTTGAACAAGTCCAAGTAATTTCACCAATTCTCACCAAAATTTAAATTGAAT
      CAGCAAGGGGAAGAAAAAGATCAGCACAGTTTAAAGGAAGAAATATGTTTTTTGTGTGTG +245
```

Figure S1C. The nucleotide sequence and deduced amino acid sequences of HmPDS. An asterisk (*) represents a stop codon.

```
-37  CGAAGATTCTCTTCTGTTTTCTCCACCTTCCATACTCTTGTAATACTCCACTAAAAACAC
     ACATTCACTTCAAATATTACTACAAAATCACACACACTAAAACACACAGTAACCAAGAAA
  1  ATGGCAAACTCACTCCTCCTCACAACTCCCTTCTCTCTACCCTCAAAACCCAAATCTTTT
      M  A  N  S  L  L  L  T  T  P  F  S  L  P  S  K  P  K  S  F
 61  CACTTCAAACTCACCTCATCTTTCACACACATCACTACAAACAACCCCTTTAAATTACCC
      H  F  K  L  T  S  S  F  T  H  I  T  T  N  N  P  F  K  L  P
121  ACCAAAAAAGCCCAAAAATCCAAACTTTTTTTCAAGAAAGTTCAAGCAATTGGAAAAACA
      T  K  K  A  Q  K  S  K  L  F  F  K  K  V  Q  A  I  G  K  T
181  GATGAAAGAACTAGTCTTGGCATTGACCCAGATGAAGAAATCTTGGTTGGTGAAGATTCT
      D  E  R  T  S  L  G  I  D  P  D  E  E  I  L  V  G  E  D  S
241  GCTGTTTTTGAGTTGGGTCAGCAGAAAGTCTCTTCTTGGGTTTATTTTAGTGGGATTTTA
      A  V  F  E  L  G  Q  Q  K  V  S  S  W  V  Y  F  S  G  I  L
301  GGGGTGGTTTTGTTTGTTCTTCAAGTGGGCTGGATTGATAATTCTACTGGGCTTGGTCAA
      G  V  V  L  F  V  L  Q  V  G  W  I  D  N  S  T  G  L  G  Q
361  GATTTTATTCAGGCTGTTTCTACCATTTCAGATAGCCCTGAGGTAGTAATGATTCTCCTC
      D  F  I  Q  A  V  S  T  I  S  D  S  P  E  V  V  M  I  L  L
421  ATTCTCATTTTCGCTGGTGTCCATAGTGGCTTAGCTAGTCTTAGAGACGCCGGAGAGAAA
      I  L  I  F  A  G  V  H  S  G  L  A  S  L  R  D  A  G  E  K
481  CTTATTGGAGAACGTGCATTTCGTGTCTTGTTTGCTGGCACGTCTCTTCCACTGGCTGTT
      L  I  G  E  R  A  F  R  V  L  F  A  G  T  S  L  P  L  A  V
541  AGTACTGTTGTGTATTTCATCAATCACAGATATGATGGCGTTCAGTTATGGCAGCTGCAG
      S  T  V  V  Y  F  I  N  H  R  Y  D  G  V  Q  L  W  Q  L  Q
601  GATGTTCCCTTGCTACATCATTTATTATGGCTCTCTAATTTTGTTTCCTTTTTCTTTCTC
      D  V  P  L  L  H  H  L  L  W  L  S  N  F  V  S  F  F  F  L
661  TATCCATCAACTTTTAATTTGTTAGAGGTAGCAGCAGTAGACAAGCCCAAAATGCATCTT
      Y  P  S  T  F  N  L  L  E  V  A  A  V  D  K  P  K  M  H  L
721  TGGGAAAGCGGGATTATGAGAATTACTAGGCACCCACAGATGGTTGGGCAGGTGATGTGG
      W  E  S  G  I  M [R  I  T  R  H  P  Q] M  V  G  Q  V  M  W
781  TGTCTTGCTCACACAATCTGGATTGGGAACACCGTGGCAGTGGCAGCTTCCGTTGGTCTA
      C  L  A  H  T  I  W  I  G  N  T  V  A  V  A  A  S  V  G  L
841  ATAGGACATCATCTATTTGGCGTTTGGAATGGTGACAGAAGGCTGGCTATTCGATACGGT
      I  G  H  H  L  F  G  V  W  N  G  D  R  R  L  A  I  R  Y  G
901  GAAGATTTCGAGCTTGTAAAAAGCCGAACAAGCGTAATACCATTTGCAGCTATTCTTGAG
      E  D  F  E  L  V  K  S  R  T  S  V  I  P  F  A  A  I  L  E
961  GGTCGTCAGAAGTTACCCAAAGATTACTACAAGGAATTTATTAGGCTGCCCTACTTAGCA
      G  R  Q  K  L  P  K  D  Y  Y  K  E  F  I  R  L  P  Y  L  A
1021 ATCACATTTGTTACTTTAGGAGCCTACTTTGCTCACCCACTAATGCAAGCTGCCAGTTTT
      I  T  F  V  T  L  G  A  Y  F  A  H  P  L  M  Q  A  A  S  F
1081 CGGCTGCATTGGTAGGTCAGAACTCACTTTGGACGCGATAAACAGAGCAAGATGTTTCAA
      R  L  H  W  *
     AGTTTCCATTTCGGAGTCTAATGTTTATGATCGGGATCACTCATAATCGTGGTCAGATGC
     CAAAGATTCGCTGGTTCACCACAATTATGCTTTTGTAAGAGATTAATGGATAGGTATATA +199
```

Figure S1D. The nucleotide sequence and deduced amino acid sequences of HmZ-ISO. An asterisk (*) represents a stop codon.

```
-166  AACCGACAAAATCTTCATCACTTCGCATCTTCTCTAATTCTACTCTCTTTTGATTCCCTT
      CCATCCTAATTTTATATGTATACTAAACTTGCTTTACATTTTGTTTATTGATTTCCTAAT
   1  ATGACATCCTCTATGTATTTTCCGCCCACTTTCCGCCCTAGTTCCGCCGGAATTCTACTC
       M  T  S  S  M  Y  F  P  P  T  F  R  P  S  S  A  G  I  L  L
  61  TCCCGTTGCCGTCCGTTAGCTCAATCCAAGCCTCATAGAATTATGATTGTTCGCTCTGAT
       S  R  C  R  P  L  A  Q  S  K  P  H  R  I  M  I  V  R  S  D
 121  TTAGAGAAAAATGTTTCCGACATGAGCACCAATGCTCCAAAAGGGCTATTTCCACCTGAA
       L  E  K  N  V  S  D  M  S  T  N  A  P  K  G  L  F  P  P  E
 181  CCAGAACACTATCGTGGACCAAAGCTGAAGGTTGCTATTATAGGAGCAGGGCTTGCGGGC
       P  E  H  Y  R  G  P  K  L  K  V  A  I  I  G  A  G  L  A  G
 241  ATGTCTACTGCTGTTGAGCTTTTAGATCAAGGCCATGAGGTGGATATATATGAATCAAGG
       M  S  T  A  V  E  L  L  D  Q  G  H  E  V  D  I  Y  E  S  R
 301  CCTTTCATTGGAGGGAAAGTGGGTTCTTTCGTTGATAAACGCGGAAATCATATAGAAATG
       P  F  I  G  G  K  V  G  S  F  V  D  K  R  G  N  H  I  E  M
 361  GGACTTCATGTATTTTTTGGTTGCTACAATAATCTTTTCCGTCTTCTAAAAAAGGTTGGT
       G  L  H  V  F  F  G  C  Y  N  N  L  F  R  L  L  K  K  V  G
 421  GCAGATAAAAATCTTCTCGTGAAGGATCATACTCACACATTTGTAAACAAAGGGGGTGAA
       A  D  K  N  L  L  V  K  D  H  T  H  T  F  V  N  K  G  G  E
 481  ATTGGTGAACTAGATTTTCGTTTTCCGGTTGGAGCGCCATTACATGGAATAAATGCCTTT
       I  G  E  L  D  F  R  F  P  V  G  A  P  L  H  G  I  N  A  F
 541  TTGACTACGAATCAACTCAAGACTTATGATAAAGCTAGAAATGCTCTTGCCCTGGCCCTC
       L  T  T  N  Q  L  K  T  Y  D  K  A  R  N  A  L  A  L  A  L
 601  AGTCCAGTTGTGCGTGCACTTGTTGACCCAGATGGAGCAATGAGGGACATAAGAAACTTG
       S  P  V  V  R  A  L  V  D  P  D  G  A  M  R  D  I  R  N  L
 661  GATGATATTAGTTTTTCTGAATGGTTCTTATCCAAAGGGGGCACACGCAAGAGTATCCAA
       D  D  I  S  F  S  E  W  F  L  S  K  G  G  T  R  K  S  I  Q
 721  AGAATGTGGGATCCTGTTGCTTATGCTCTCGGGTTTATTGACTGTGATAACATGAGTGCT
       R  M  W  D  P  V  A  Y  A  L  G  F  I  D  C  D  N  M  S  A
 781  CGTTGTATGCTCACTATTTTCTCATTGTTTGCCACTAAAACAGAAGCATCCCTTTTGCGC
       R  C  M  L  T  I  F  S  L  F  A  T  K  T  E  A  S  L  L  R
 841  ATGCTTAAAGGTTCTCCTGATGTTTATTTAAGTGGACCTATTAGAGACTACATTACAGAA
       M  L  K  G  S  P  D  V  Y  L  S  G  P  I  R  D  Y  I  T  E
 901  AAAGGGGGAAGGTTCCATCTCAGGTGGGGATGTCGAGAGGTTCTTTATGAAAAAACTATG
       K  G  G  R  F  H  L  R  W  G  C  R  E  V  L  Y  E  K  T  M
 961  GATGGCCAAACATACATCTCAGGAATTGCCATGTCTAAGGCAACTCAAAAGAAAGTCGTG
       D  G  Q  T  Y  I  S  G  I  A  M  S  K  A  T  Q  K  K  V  V
1021  AAAGCAGATGCTTATGTTGCGGCTTGTGATGTCCCTGGGATCAAAAGATTATTGCCTTCA
       K  A  D  A  Y  V  A  A  C  D  V  P  G  I  K  R  L  L  P  S
1081  CAGTGGAGAGAATGGGAGTTCTTCGACAATATATACAAACTAGTTGGTGTTCCTGTTGTT
       Q  W  R  E  W  E  F  F  D  N  I  Y  K  L  V  G  V  P  V  V
1141  ACTGTTCAACTTAGATACAACGGCTGGGTTACAGAGATGCAGGATCTAGAAAGGTCAAGG
       T  V  Q  L  R  Y  N  G  W  V  T  E  M  Q  D  L  E  R  S  R
1201  CAACTGAGGCAAGCAGCGGGACTGGATAATCTCCTTTATTCCCCAGATGCAGACTTCTCT
       Q  L  R  Q  A  A  G  L  D  N  L  L  Y  S  P  D  A  D  F  S
1261  TGTTTTGCAGACTTAGCACTTGCATCTCCAGAAGATTACTATCTTGAGGGTCAAGGCTCG
       C  F  A  D  L  A  L  A  S  P  E  D  Y  Y  L  E  G  Q  G  S
1321  TTGCTTCAATGTGTGCTCACCCCTGGTGATCCCTACATGCCTTTACCAAATGATCAAATC
       L  L  Q  C  V  L  T  P  G  D  P  Y  M  P  L  P  N  D  Q  I
1381  ATAGAGAGAGTTACCAAGCAGGTCTTGACTTTGTTCCCATCCTCCCAAGGCTTGGAAGTT
       I  E  R  V  T  K  Q  V  L  T  L  F  P  S  S  Q  G  L  E  V
1441  ACATGGTCATCTGTTGTCAAGATTGCGCAGTCATTATATCGTGAGGGACCTGGTAAAGAT
       T  W  S  S  V  V  K  I  A  Q  S  L  Y  R  E  G  P  G  K  D
1501  CCTTTTAGACCTGATCAGAAGACCCCTGTTGGAAACTTTTTTCTTGCTGGCTCCTATACA
       P  F  R  P  D  Q  K  T  P  V  G  N  F  F  L  A  G  S  Y  T
1561  AAACAGGATTATATAGACAGTATGGAAGGTGCAACTCTTTCGGGAAGGCAAGCTTCTGCC
       K  Q  D  Y  I  D  S  M  E  G  A  T  L  S  G  R  Q  A  S  A
1621  TTGATATGTGACGCTGGAGAAGATTTGATGGCCCTGCAAAAAACAATTGCTTCAATCGAC
       L  I  C  D  A  G  E  D  L  M  A  L  Q  K  T  I  A  S  I  D
1681  TCCAACAGACCTACAGAAGCTGAGTTAAGCCTTGTCTGATGCACAATGATATAAATATGA
       S  N  R  P  T  E  A  E  L  S  L  V  *
      ACATAAAGGCAGACCTCCGACCAAATCAGGATATCTTGGATCGACGACTAGAAAGCAATC
      ATGCACATGAATGTACTTTCTTATAGCTGAATTGAACCATGAGAACCTGATGGAACGTCG +1381
```

Figure S1E. The nucleotide sequence and deduced amino acid sequences of HmZDS. An asterisk (*) represents a stop codon.

```
-168  ATAAAACCATACATACACTCACCTTGATCCACAAAACTAAGTTTTCTCTTCATCTTTCTT
      GATTTTAACACCCACAATTTTTATACCCAGAAACTAAAATTTAAGATATCCTATATTACT
   1  ATGTCTACTAGCATCTTTGAAACCCCATTAACAAGAAGTAATCTTTTTTTATGTAACACT
       M  S  T  S  I  F  E  T  P  L  T  R  S  N  L  F  L  C  N  T
  61  AGTTTATTTTCTCAAAGTTTCAAACTTTTTAATAATTCCAGCACTTTTGAGTTAAAAAGC
       S  L  F  S  Q  S  F  K  L  F  N  N  S  S  T  F  E  L  K  S
 121  TTAAGACCCAGATGTCAAAAACATAGAATTTCATGTTTAAAGCCTTTGAATTTGAGTTTA
       L  R  P  R  C  Q  K  H  R  I  S  C  L  K  P  L  N  L  S  L
 181  TTTCATGATGATGATAGGAAATTTGGGTTTTTTAGGCTGAATAGGAGAGAGAGAAAGTCC
       F  H  D  D  D  R  K  F  G  F  F  R  L  N  R  R  E  R  K  S
 241  AATTTTGCATTGAATTCTGTGTTGAATGTAGAGAAAGAAGTGGAGAGTGATGAAAGTGTG
       N  F  A  L  N  S  V  L  N  V  E  K  E  V  E  S  D  E  S  V
 301  GGATTGGGTAGGAGTAGAGAGTATGATGCTATTGTGATTGGGTCAGGGATTGGTGGTTTA
       G  L  G  R  S  R  E  Y  D  A  I  V  I  G  S  G  I  G  G  L
 361  GTTGCAGCTACACAGTTGGCTGTGAAGGGAGCTAAGGTTTTGGTTCTCGAAAAATATTTG
       V  A  A  T  Q  L  A  V  K  G  A  K  V  L  V  L  E  K  Y  L
 421  ATTCCTGGTGGGAGCTCTGGATATTATGAAAGGGATGGTTTTACATTTGATGTTGGATCA
       I  P  G  G  S  S  G  Y  Y  E  R  D  G  F  T  F  D  V  G  S
 481  TCCGTGATGTTCGGCTTTAGTGATAAGGGGAATCTAAACTTGATAACTCAAGCATTGGCA
       S  V  M  F  G  F  S  D  K  G  N  L  N  L  I  T  Q  A  L  A
 541  GCAGTTGGCTGTAAGATGGAGGTGATACCTGACCCCAGTACCGTGCATTTTCATCTACCT
       A  V  G  C  K  M  E  V  I  P  D  P  S  T  V  H  F  H  L  P
 601  AAAAATCTTTCTGTTCGAGTACATAGAGAATATAATGAGTTTTTCACCGAACTCACAAGC
       K  N  L  S  V  R  V  H  R  E  Y  N  E  F  F  T  E  L  T  S
 661  AAATTTCCACATGAAAAAGAAGGAATATATAAATTTTACGGTGAATGCTGGAAGATTTTC
       K  F  P  H  E  K  E  G  I  Y  K  F  Y  G  E  C  W  K  I  F
 721  AATGCCTTGAACTCATTGGAACTGAAGTCACTCGAGGAGCCAATCTACCTGTTCGGACAG
       N  A  L  N  S  L  E  L  K  S  L  E  E  P  I  Y  L  F  G  Q
 781  TTCTTTAAAAAGCCTATGGAATGCCTAACCCTAGCATACTATCTGCCTCAAAATGCCGGG
       F  F  K  K  P  M  E  C  L  T  L  A  Y  Y  L  P  Q  N  A  G
 841  GACATAGCTCGGAAGTACATAAAAGATCAGCAGGTGCTGTCCTTTATAGATGCTGAGTGC
       D  I  A  R  K  Y  I  K  D  Q  Q  V  L  S  F  I  D  A  E  C
 901  TTTATTGTGAGTACTGTCAATGCATTGAAGACACCAATGATCAACGCAAGCATGGTTCTA
       F  I  V  S  T  V  N  A  L  K [T  P  M  I  N  A  S  M  V] L
 961  TGTGACAGACACTATGGGGGAATTAACTATCCGGTTGGTGGGGTTGGCGGGATAGCAAAA
       C  D  R  H  Y  G  G  I  N  Y  P  V  G  G  V  G  G  I  A  K
1021  TCATTAGCGAAAGGATTAGTCGATCAGGGAAGCGAAATACATTACAAAGCAAATGTTAAG
       S  L  A  K  G  L  V  D  Q  G  S  E  I  H  Y  K  A  N  V  K
1081  AGCATTATAGTTGAAAATGGAAAAGCTGTAGGAGTTAGGCTGGTGAATGGAAATGAGTTA
       S  I  I  V  E  N  G  K  A  V  G  V  R  L  V  N  G  N  E  L
1141  TTTGCAAAGAACATAATTTCAAATGCTACCAGATGGGATACTTTTGGGAAGCTTTTAAAA
       F  A  K  N  I  I  S  N  A  T  R  W  D  T  F  G  K  L  L  K
1201  CAGGATGAATTACCTAAAGAAGAAGAAAATTTTCAGAAACTTTACATCAAGGCCCCATCA
       Q  D  E  L  P  K  E  E  E  N  F  Q  K  L  Y  I  K  A  P  S
1261  TTTCTTTCTATTCATTTGGGCGTTAAATCCGATGTTTTGCCACCAGATACGGATTGCCAC
       F  L  S  I  H  L  G  V  K  S  D  V  L  P  P  D  T  D  C  H
1321  CATTTTGTTCTGGAGGATGATTGGTCAAATTTAGAGGTGCCATATGGTAGTATATTCCTG
       H  F  V  L  E  D  D  W  S  N  L  E  V  P  Y  G  S  I  F  L
1381  AGCATTCCGACTGTTCTGGATTCATCATTAGCTCCAGAAGGGAATCATATTCTTCACATA
       S  I  P  T  V  L  D  S  S  L  A  P  E  G  N  H  I  L  H  I
1441  TTTACAACTTCTTCCATAGAGGACTGGCAGGGGATCTCACAAAAAGATTATGAGAAAAAA
       F  T  T  S  S  I  E  D  W  Q  G  I  S  Q  K  D  Y  E  K  K
1501  AAGGAGCTTGTCGCTGACAAGATTATAAGCCGACTGGAAAAGAAACTTTTTCCTGGACTC
       K  E  L  V  A  D  K  I  I  S  R  L  E  K  K  L  F  P  G  L
1561  AAGTCTTCCATTGTTTTTAAGGAGGTTGGGACACCAAAGACACACAGACGATACCTTGCT
       K  S  S  I  V  F  K  E  V  G  T  P  K  T  H  R  R  Y  L  A
1621  CGTGATAGTGGTACCTATGGACCAATGCCACAGGGTACTCCAAAGGGGTTATTGGGAATG
       R  D  S  G  T  Y  G  P  M  P  Q  G  T  P  K  G  L  L  G  M
1681  CCATTTAATACAACTGCAATTGATGGTTTGTACTGTGTTGGGGATAGTTGCTTTCCAGGA
       P  F  N  T  T  A  I  D  G  L  Y  C  V  G  D  S  C  F  P  G
1741  CAAGGTGTTATAGCTGTTGCATTCTCAGGAGTGATGTGTGCTCATCGGGTAGCTGCTGAT
       Q  G  V  I  A  V  A  F  S  G  V  M  C  A  H  R  V  A  A  D
1801  TTAGGGCTTGAGCAGAAGTCTCCCTTACTGGATGCTGCTCTTCTTCGCCTACTGGGTTGG
       L  G  L  E  Q  K  S  P  L  L  D  A  A  L  L  R  L  L  G  W
1861  TTCAGGACACTGGCATAAAGAGTTTAAAAGCAAAATGTGACTTATATGAAGGGCAGGAGT
       F  R  T  L  A  *
      TTGCATCTGATAAGAGGTCATGATAATTTTGATATATTACTCTCTCTAAAGAATTCAATC
      GAGGTATGATGGAACTAGGGAAAGTTATTCTTTATTTTGTGAACTACAGCAAATTTATCT +63
```

Figure S1F. The nucleotide sequence and deduced amino acid sequences of HmCrtISO. An asterisk (*) represents a stop codon.

```
-232  AGTTACTTAATTTGTCAATCAATTATTGGTGAACATTAGTGAAAAGGTTGAGAATTCTTG
      GAACCCAGATTTTGTTTATTGGAAATTTGATTGAATTTCATAGAACCATACATAAAAGTG
   1  ATGGATACTTTACTAAAAACTCATAATAAGCTTGAATTTTTCAATCCAATTCATGGATTT
       M  D  T  L  L  K  T  H  N  K  L  E  F  F  N  P  I  H  G  F 
  61  CCTGATAAAGTTGGAACCTTGAGTTCTTTGAAGTTTCGAAACCAGGAGGTGAGGTTTGGT
       P  D  K  V  G  T  L  S  S  L  K  F  R  N  Q  E  V  R  F  G 
 121  CCTAAAAGATCTCATGTGAGTTGGGGAAAGAATGGTTCTGTAAAGGCTAGTAGTAGTGCC
       P  K  R  S  H  V  S  W  G  K  N  G  S  V  K  A  S  S  S  A 
 181  CTTTTGGAGCTTGTTCAAGAAACCAAGAAGGAAAATCTTGAATTTGACCTTCCTTTGTAT
       L  L  E  L  V  Q  E  T  K  K  E  N  L  E  F  D  L  P  L  Y 
 241  GACCCGTCGAATGGTCTCGTAGTGGATTTGGCTGTAGTTGGTGGCGGTCCTGCAGGGTTA
       D  P  S  N  G  L  V  V [D  L  A  V  V  G  G  G] P  A  G  L 
 301  GCGGTAGCACAACAAGTTTCAGAGGCAGGACTTTCGGTTGTGTCTATAGACCCGTCTCCG
       A  V  A  Q  Q  V  S  E  A  G  L  S  V  V  S  I  D  P  S  P 
 361  AAATTGATATGGCCCAACAATTATGGTGTTTGGGTGGATGAGTTTGAGGCTATGGATTTA
       K  L  I  W  P  N  N  Y  G  V  W  V  D  E  F  E  A  M  D  L 
 421  CTAGATTGCCTTGACACTACCTGGTCAAGTGCGATTGTTTACATTGATGACGAGACAACC
       L  D  C  L  D  T  T  W  S  S  A  I  V  Y  I  D  D  E  T  T 
 481  AAGGAGCTTGGAAGACCGTATGGAAGGGTCAACAGGAAGCAGCTTAAATCAAAGATGATG
       K  E  L  G  R  P  Y  G  R  V  N  R  K  Q  L  K  S  K  M  M 
 541  CAGAAGTGCATATCAAATGGGGTTAAATTTCATCAGGCCAAGGTTGTCAAAGTTGTACAC
       Q  K  C  I  S  N  G  V  K  F  H  Q  A  K  V  V  K  V  V  H 
 601  GAGGAAGCCAAATCTTTATTGATATGTAATGATGGTGTGACCATTCAAGCTACTGTAGTT
       E  E  A  K  S  L  L  I  C  N  D  G  V  T  I  Q  A  T  V  V 
 661  CTTGATGCCACCGGTTTTTCAAGATGTCTTGTTCAATATGACAAGCCATATAATCCAGGA
       L  D  A  T  G  F  S  R  C  L  V  Q  Y  D  K  P  Y  N  P  G 
 721  TACCAAGTAGCTTACGGAATTGTGGCAGAAGTAGAAGAACACCCTTTTGATGTAAATAAG
       Y  Q  V  A  Y  G  I  V  A  E  V  E  E  H  P  F  D  V  N  K 
 781  ATGCTTTTCATGGATTGGAGAGATTCCCATCTCGATGGCAATGCAGAATTAAAAGAACGA
       M  L  F  M  D  W  R  D  S  H  L  D  G  N  A  E  L  K  E  R 
 841  AACAGTAAAATTCCTACGTTTCTTTATGCAATGCCCTTTTCATCAGACAGAATATTTCTT
       N  S  K  I  P  T  F  L  Y  A  M  P  F  S  S  D  R  I  F  L 
 901  GAAGAAACATCCCTTGTAGCTCGTCCTGGTTTAGCCATGGGAGATATTCAGGAGAGAATG
       E  E  T  S  L  V  A  R  P  G  L  A  M  G  D  I  Q  E  R  M 
 961  GTGGCTCGCTTAAGGCATTTGGGTATTAAAGTGAAGAGCATTGAAGAGGATGAGAGATGT
       V  A  R  L  R  H  L  G  I  K  V  K  S  I  E  E  D  E  R  C 
1021  GTAATCCCAATGGGAGGGCCTCTACCTGTACTCCCTCAAAGAGTTGTAGGAATTGGCGGT
       V  I  P  M  G  G  P  L  P  V  L  P  Q  R  V  V  G  I  G  G 
1081  ACAGCTGGTATGGTGCATCCTTCAACTGGATATATGGTAGCAAGGACTCTAGCTGCTGCA
       T  A  G  M  V  H  P  S  T  G  Y  M  V  A  R  T  L  A  A  A 
1141  CCAATTGTTGCCAATGCAATTGTTCAGTACCTTGGTGGTTCTAAGAAAGGCACTTTGGGA
       P  I  V  A  N  A  I  V  Q  Y  L  G  G  S  K  K  G  T  L  G 
1201  AACGAATTGTCTGCAGAAGTTTGGAAAGATCTATGGCCAATAGAGAGAAGACGCCAAAGA
       N  E  L  S  A  E  V  W  K  D  L  W  P  I  E  R  R  R  Q  R 
1261  GAATTCTTTTGCTTTGGTATGGATGTTTTGCTCAAACTTGATTTGCCTGGTACGAGAAGG
       E  F  F  C  F  G  M  D  V  L  L  K  L  D  L  P  G  T  R  R 
1321  TTTTTCAGTGCTTTTTTTGACCTTGAACCTCGCTATTGGCATGGATTCTTGTCTTCTCGG
       F  F  S  A  F  F  D  L  E  P  R  Y  W  H  G  F  L  S  S  R 
1381  CTATTTCTTCCTGAACTTTTCTTTTTCGGGCTTTCTCTTTTCTCAAATGCCTCCAACACT
       L  F  L  P  E  L  F  F  F  G  L  S  L  F  S  N  A  S  N  T 
1441  TCTAGAATAGAAATCATGGCAAAAGGCACAGTTCCTTTAGTAAACATGGTAAACAATCTA
       S  R  I  E  I  M  A  K  G  T  V  P  L  V  N  M  V  N  N  L 
1501  ATCAAAGATAGAGAATAGTCATTTTGTGTTATTGACTCAATTGTATTATATATATAATGA
       I  K  D  R  E  * 
      TGTTCTTCGATTTAGTTCAAAATGCTGGTAAAGTTACACATCTTACTTGTTATCCACTTT
      CCGGTTCAAGCATTCTGAGCTTGTACATTTCCTGAAGGAGCAACATGGATTTACATGTCA +155
```

Figure S1G. The nucleotide sequence and deduced amino acid sequences of HmLCYB. An asterisk (*) represents a stop codon.

```
 -11  CCCCTATCTCTTCCCTCATTCACTTCACATTTCCCCAGAAACATCTACACAATCTCCACC
      GTAATGCCGCCGTAACTAACTCTACATACACACACAATAACACACATTCTCGCCGGAACA
   1  ATGGAGACGTACTGTATAGGAGGCCGAAACTTCACAACAATGGTGGCGTTTTCGACGTGT
       M  E  T  Y  C  I  G  G  R  N  F  T  T  M  V  A  F  S  T  C
  61  CCGACGTGGAGGAGACCGAGAAGGAAGAGACTGAGAAGAAATGTGAAGATGAGTTGTGGT
       P  T  W  R  R  P  R  R  K  R  L  R  R  N  V  K  M  S  C  G
 121  AGAAAGAGTGAATTGAGATGTGTGAAGGAGAGTATAAAGTCTGTTGCGGTTGTTGAAGAG
       R  K  S  E  L  R  C  V  K  E  S  I  K  S  V  A  V  V  E  E
 181  GAGGAGTTTGCCGACGAAGAGGATTTTGTGAAAGGCGGTGGCTCGGAGATGGTGTTCGTT
       E  E  F  A  D  E  E  D  F  V  K  G  G  G  S  E  M  V  F  V
 241  CAAATGCAGAGGAATAAGGCTATGGATAGTCAGTCTAGACTTTCTCATAAATTGCCGCAA
       Q  M  Q  R  N  K  A  M  D  S  Q  S  R  L  S  H  K  L  P  Q
 301  ATACCAATTAGAGATAGTGTATTGGATTTGGTGGTTATTGGCTGTGGTCCTGCCGGTCTT
       I  P  I  R  D  S  V  L  D  L  V  V  I  G  C  G  P  A  G  L
 361  GCTCTTGCTGCAGAATCAGCTAAGCTAGGTCTGAGAGTTGGGCTTATTGGCCAGATCTT
       A  L  A  A  E  S  A  K  L  G  L  R  V  G  L  I  G  P  D  L
 421  CCGTTTACAAATAATTATGGCGTATGGGAGGATGAATTTATAGATCTTGGACTCGAAGGG
       P  F  T  N  N  Y  G  V  W  E  D  E  F  I  D  L  G  L  E  G
 481  TGTATTGAGCATGTTTGGCGGGATACTATTGTATATCTTGACGATGGTGATCCTATTATG
       C  I  E  H  V  W  R  D  T  I  V  Y  L  D  D  G  D  P  I  M
 541  ATCGGCCGTGCTTATGGAAGAGTTAGTCGCCATTTGCTTCATGAAGAATTGCTTAAGAGG
       I  G  R  A  Y  G  R  V  S  R  H  L  L  H  E  E  L  L  K  R
 601  TGTGTCGAGTCAGGTGTCTCGTATCTTAGCTCAAAAGTTGAAAAGATTATCGAAGCTGGA
       C  V  E  S  G  V  S  Y  L  S  S  K  V  E  K  I  I  E  A  G
 661  GATGGCCATAGCCTGGTCGAGTGTGAAAATAATATTGTCATTCCATGCAGGCTTGCTACT
       D  G  H  S  L  V  E  C  E  N  N  I  V  I  P  C  R  L  A  T
 721  GTTGCATCTGGAGCAGCCTCTGGGAAACTTTTGCAGTATGAGGTGGGAGGTCCCAGAGTT
       V  A  S  G  A  A  S  G  K  L  L  Q  Y  E  V  G  G  P  R  V
 781  TCCGTCCAAACAGCTTATGGTGTCGAGGTCGAGGTGGAAAACAATCCATATGATCCCAGC
       S  V  Q  T  A  Y  G  V  E  V  E  V  E  N  N  P  Y  D  P  S
 841  TTGATGGTTTTCATGGATTACAGAGATTACACCAAACAAAAAGTTCCGAGCATGGAGGCA
       L  M  V  F  M  D  Y  R  D  Y  T  K  Q  K  V  P  S  M  E  A
 901  GAATATCCAACGTTTCTTTATGTGATGCCAATGTCCCCGACAAGGATTTTCTTTGAGGAA
       E  Y  P  T  F  L  Y  V  M  P  M  S  P  T  R  I  F  F  E  E
 961  ACATGTTTGGCTTCAAAAGATGCGATGCCATTCGATCTACTGAAGAAAAAACTCATGTCA
       T  C  L  A  S  K  D  A  M  P  F  D  L  L  K  K  K  L  M  S
1021  AGATTACAGACAATGGGAATCCGAGTTACGAAGACATATGAAGAGGAATGGTCTTACATA
       R  L  Q  T  M  G  I  R  V  T  K  T  Y  E  E  E  W  S  Y  I
1081  CCCGTTGGGGGATCTTTACCTAATACTGAGCAAAAAAACCTTGCATTTGGTGCAGCTGCG
       P  V  G  G  S  L  P  N  T  E  Q  K  N  L  A  F  G  A  A  A
1141  AGCATGGTGCATCCCGCCACTGGCTATTCGGTTGTCAGATCATTGTCGGAGGCCCCAGAC
       S  M  V  H  P  A  T  G  Y  S  V  V  R  S  L  S  E  A  P  D
1201  TACGCAGCTGTAATTGCAAACATCTTAAAAAATGGTCAAATCAATGGGATGATTAATCGT
       Y  A  A  V  I  A  N  I  L  K  N  G  Q  I  N  G  M  I  N  R
1261  GGAAGATATACAGAGAACATCTCAATGCAAGCTTGGAAGACCCTGTGGCCTCAAGAAAGG
       G  R  Y  T  E  N  I  S  M  Q  A  W  K  T  L  W  P  Q  E  R
1321  AAACGCCAGAGAGCGTTCTTTTTGTTTGGACTAGCACTAATTCTACAGCTGGATATTGAC
       K  R  Q  R  A  F  F  L  F  G  L  A  L  I  L  Q  L  D  I  D
1381  GGCATAAGGACATTCTTCCAAACTTTTTTCCGCTTACCCACCTGGATGTGGCAAGGATTT
       G  I  R  T  F  F  Q  T  F  F  R  L  P  T  W  M  W  Q  G  F
1441  CTTGGTTCTTCTCTCTCCTCAGGCGACCTTGTACTATTTGCCTTTTATATGTTTATCATA
       L  G  S  S  L  S  S  G  D  L  V  L  F  A  F  Y  M  F  I  I
1501  GCACCACACCACTTAAGAATGTCCCTTGTAAGACATCTCCTTTCTGATCCTACTGGAGCA
       A  P  H  H  L  R  M  S  L  V  R  H  L  L  S  D  P  T  G  A
1561  ACAATGATAAGAACATATCTAACGGTCTAGAAGCCCTATTCTGTATTTCTTAGCCAAAAT
       T  M  I  R  T  Y  L  T  V  *
      GCTCGGTTCAGTAAATTATGTAAATAACTTTTTTTAATATATATATATAATTGATAATTT
      TGATCCACACACATTACATGTTCTTTAGCTTTCAATCCAGAGTCATTTTTAATCTGTGCC +12
```

Figure S1H. The nucleotide sequence and deduced amino acid sequences of HmLCYE. An asterisk (*) represents a stop codon.

```
-187 CCACAAAACACATGTGGTTCACCTCCTTTTAAACCCCTCCAAGAATCACATGCCCCTGCC
     CGAATATCTCTTCTCAAACAACCAAAACACACAAACAGAGACGAATTCCTTGGTTCGATT
   1 ATGGCAGCTAGAATCTCGGTCGGGTCATTTCGTGTTGGAAAAGGCCAAATTCAGGGTCCG
      M  A  A  R  I  S  V  G  S  F  R  V  G  K  G  Q  I  Q  G  P
  61 AAACAGATGACCACTGTCACGCCAATTTGGCAACTATCTCCTGCGATTCGTAGATCATTT
      K  Q  M  T  T  V  T  P  I  W  Q  L  S  P  A  I  R  R  S  F
 121 GTAGGTCTAAGGTTGGGAAGGAAGCAAAAATTGAGCATTTGTTTTGCGGTGGAGGAAAGT
      V  G  L  R  L  G  R  K  Q  K  L  S  I  C  F  A  V  E  E  S
 181 GAGAGTCTCGCAGTGGATGCAGAAGTAGAAGAGAGTTCGCAAGATATTGAAAAACGCATC
      E  S  L  A  V  D  A  E  V  E  E  S  S  Q  D  I  E  K  R  I
 241 ATAGCATCCTTCACTGAGACAGGAGGCAATCGTGGTGAGGAAAGAATGGCCAGGAAGAAA
      I  A  S  F  T  E  T  G  G  N  R  G  E  E  R  M  A  R  K  K
 301 ACAGAGCGGTTCACTTATTTGGTTGCGGCTGTCATGTCTAGTTTTGGTATTACATCCATG
      T  E  R  F  T  Y  L  V  A  A  V  M  S  S  F  G  I  T  S  M
 361 GCTGTCTTCGCTGTCTACTCCAGGTTCGCTTGGCAAATGGAGGGTGGAGAGATTCCGTTG
      A  V  F  A  V  Y  S  R  F  A  W  Q  M  E  G  G  E  I  P  L
 421 TCAGAAATGCTTGGTACATTTTCACTTGCTTTCGGAGCTGCAGTCGGCATGGAGTACTGG
      S  E  M  L  G  T  F  S  L  A  F  G  A  A  V  G  M  E  Y  W
 481 GCGAGATGGGCACACGAATCGCTATGGCACGATTCGTTGTGGCACATGCACGAGTCACAC
      A  R  W  A  H  E  S  L  W  H  D  S  L  W  H  M  H  E  S  H
 541 CACAAACCAAGAGAAGGCGCATTTGAGCTGAACGATGTATTTGCTATAATAAACGCGGTT
      H  K  P  R  E  G  A  F  E  L  N  D  V  F  A  I  I  N  A  V
 601 CCAGCTATAGCACTTCTTGCCTATGGTTTCTTCCACAAAGGCCTCTTTCCTGGCCTTTGT
      P  A  I  A  L  L  A  Y  G  F  F  H  K  G  L  F  P  G  L  C
 661 TTCGGTGCTGGACTCGGAATTACGGTGTTTGGGATGGCCTACATGTTTGTCCACGACGGT
      F  G  A  G  L  G  I  T  V  F  G  M  A  Y  M  F  V  H  D  G
 721 CTTGTTCATAAACGCTTTCCTGTAGGTCCTGTAGCAAATGTCCCCTACTTGAGAAAAGTT
      L  V  H  K  R  F  P  V  G  P  V  A  N  V  P  Y  L  R  K  V
 781 GCCGCTGCTCACCAGCTGCACCACATGGAGAAGTTCAATGGAGTACCGTTTGGGTTGTTC
      A  A  A  H  Q  L  H  H  M  E  K  F  N  G  V  P  F  G  L  F
 841 TTAGGGCCTCAGGAAGTTGAACAAGTGGGAGGGACCGAAGAGCTGGAGAAAGAAATCAAT
      L  G  P  Q  E  V  E  Q  V  G  G  T  E  E  L  E  K  E  I  N
 901 CGGAGAATCAAATCCTCTTCCTAATAATTTAATGTACATTAGTTAAATCAAAGTATAATT
      R  R  I  K  S  S  S  *
     TTCTAGTTCCTTGTTAGTTGTTTTGGACGTTTGGCTTCTGTTGCTCATGACTATACGATT
     ATTGTATTTCTAAAATCCAATTCTAACTCCCACACCCAGTAAATATGTATACATGATAAA +57
```

Figure S1I. The nucleotide sequence and deduced amino acid sequences of HmCHXB. An asterisk (*) represents a stop codon.

```
                                        -3 ACTTTGAGTTACCAAACTCCAAAAC
   1 ATGCCTTCACATTCTATCTCTTCCCTCTCCCTCCTCCCCATTCCTATCCCCCAAGTCCAA
      M  P  S  H  S  I  S  S  L  S  L  L  P  I  P  I  P  Q  V  Q
  61 CCCTTGTCCAAACACCACTCACCATTTTATAGACCACCCCCATTTTATCACTCACTCTCC
      P  L  S  K  H  H  S  P  F  Y  R  P  P  P  F  Y  H  S  L  S
 121 ATCAAATCTTCACTAGACAACAATAATAATAAGCCACCACCCCACAAGTCCAACCAAGGC
      I  K  S  S  L  D  N  N  N  N  K  P  P  P  H  K  S  N  Q  G
 181 TCATGGGTCAGCCCAGACTGGCTCACTTCTTTAACCAAATCTTTAAGTTTAACTAAAAAT
      S  W  V  S  P  D  W  L  T  S  L  T  K  S  L  S  L  T  K  N
 241 GATGATTCTAACATACCCATTGCTAGTGCTAAACTTGATGATGTTTCTGAGCTTCTTGGT
      D  D  S  N  I  P  I  A  S  A  K  L  D  D  V  S  E  L  L  G
 301 GGTGCTCTTTTCTTACCTTTGTTTAAATGGATGAATCTTTATGGGCCTATTTATAGACTT
      G  A  L  F  L  P  L  F  K  W  M  N  L  Y  G  P  I  Y  R  L
 361 GCTGCTGGGCCTAGAGATTTTGTGGTGGTTAGTGATCCAGCTATTGCTAAACATGTTTTG
      A  A  G  P  R  D  F  V  V  V  S  D  P  A  I  A  K  H  V  L
 421 AGGAATTATGGGAGGTATGCTAAAGGTCTTGTTGCTGAGGTTTCTGAGTTTCTTTTTGGG
      R  N  Y  G  R  Y  A  K  G  L  V  A  E  V  S  E  F  L  F  G
 481 TCTGGTTTTGCTATTGCTGAAGGCCCTCTTTGGACGGCTAGGCGCAGGGCTGTAGTTCCG
      S  G  F  A  I  A  E  G  P  L  W  T  A  R  R  R  A  V  V  P
 541 TCGCTTCACAAAAAGTATTTGTCAGTGATGGTTGATCGAGTGTTCTGCAAATGTGCCGAG
      S  L  H  K  K  Y  L  S  V  M  V  D  R  V  F  C  K  C  A  E
 601 CGGCTGGTGGAAAAGCTTGAAACTTCCGCAGAAAATGGCTCTGCTGTAAACATGGAGGCA
      R  L  V  E  K  L  E  T  S  A  E  N  G  S  A  V  N  M  E  A
 661 CAGTTTTCTCAGTTGACCCTCGACGTTATTGGTCTATCAGTGTTCAACTACAACTTTGAT
      Q  F  S  Q  L  T  L  D  V  I  G  L  S  V  F  N  Y  N  F  D
 721 TCACTTAATACTGATAGTCCTGTTATTGAAGCAGTTTACACCGCGTTAAAAGAAGCAGAG
      S  L  N  T  D  S  P  V  I  E  A  V  Y  T  A  L  K  E  A  E
 781 GCTCGATCAACTGACATATTACCATACTGGAAGATTCAAGCATTATGTAAAATTATCCCG
      A  R  S  T  D  I  L  P  Y  W  K  I  Q  A  L  C  K  I  I  P
 841 AGACAAATAAAAGCTGAGCAAGCAGTTACGGTGATCAGAACAACAGTTGAAGAACTAATC
      R  Q  I  K  A  E  Q  A  V  T  V  I  R  T  T  V  E  E  L  I
 901 GAGAAGTGCAAGCAGATTGTGGAAACTGAAGGTGAAAGGATTAGTGAGGAGGAGTATGTG
      E  K  C  K  Q  I  V  E  T  E  G  E  R  I  S  E  E  E  Y  V
 961 AATGAAGCTGATCCAAGCATCTTGCGGTTCCTGCTCGCTAGCAGAGAAGAGGTTTCAAGC
      N  E  A  D  P  S  I  L  R  F  L  L  A  S  R  E  E  V  S  S
1021 ACACAGCTGCGAGATGACCTTTTGTCAATGTTAGTTGCTGGTCACGAGACCACCGGCTCT
      T  Q  L  R  D  D  L  L  S  M  L  V  A  G  H  E  T  T  G  S
1081 GTGTTGACATGGACATCATATCTTTTAAGTAAGAATCCTTCCTCTTTAGCAAAAGCCCAG
      V  L  T  W  T  S  Y  L  L  S  K  N  P  S  S  L  A  K  A  Q
1141 GAAGAAGTTGATAGAGTTTTACAAGGAAGGCCTCCTACATATGATGACATCAAGAATCTT
      E  E  V  D  R  V  L  Q  G  R  P  P  T  Y  D  D  I  K  N  L
1201 AAGTACCTGACACGTTGCATAAATGAGTCAATGCGTCTTTACCCACATCCTCCTGTTCTC
      K  Y  L  T  R  C  I  N  E  S  M  R  L  Y  P  H  P  P  V  L
1261 ATTCGAAGAGCTCAAGTTGCTGATGAACTTCCTGGCAACTACAAAGTTAATCCCGGTCAA
      I  R  R  A  Q  V  A  D  E  L  P  G  N  Y  K  V  N  P  G  Q
1321 GACATAATGATTTCGGTATATAATATCCATCATTCATCAACGGTCTGGGACAGAGCAGAA
      D  I  M  I  S  V  Y  N  I  H  H  S  S  T  V  W  D  R  A  E
1381 GAGTTTATTCCAGAAAGATTTGACTTGGATGGCCCTGTGCCAAATGAATCAAATACTGAT
      E  F  I  P  E  R  F  D  L  D  G  P  V  P  N  E  S  N  T  D
1441 TATAGATTTATCCCATTCAGTGGAGGTCCTCGCAAATGTGTAGGTGATCAGTTTGCTTTA
      Y  R  F  I  P  F  S  G  G  P  R  K  C  V  G  D  Q  F  A  L
1501 CTAGAAGCAATTGTATCACTAGCTATATTCCTGCAGCACTTGAGCTTTGAGTTAATTCCA
      L  E  A  I  V  S  L  A  I  F  L  Q  H  L  S  F  E  L  I  P
1561 GATCAGGTTATCAACATGACAACAGGAGCCACTATTCACACGACGAATGGCTTGTACATG
      D  Q  V  I  N  M  T  T  G [A  T  I  H  T] T  N  G  L  Y  M
1621 AAACTGTCCAAGCGGCAAATCAAATCAGCAGCTGTTTCCTCAGTTTCTAGGTAAACTTTG
      K  L  S  K  R  Q  I  K  S  A  A  V  S  S  V  S  R  *
     CAGTTCTGTACATAAAAGCGGCAATAGTTTGTATAGAATGTGGGATACTGAAGTAAGGAG
     ATCTCTGATTCTTCTGATTTCTTAATCGAGAAAGAACATTTCATTGTGCAGCTGAGAACA +165
```

Figure S1J. The nucleotide sequence and deduced amino acid sequences of HmCHXE. An asterisk (*) represents a stop codon.

```
-230 ACATTCACAAATTCATGGCCTTGGATTGGTAAGCACAACTATACTATACACTACCTGTTT
     CATTACCAGAACCAATCAAGAACAAGAATTTAGAAACCTTAGTGTCTTGCTTGTCTGAAC
   1 ATGGCTTCAGCTGTGTTCTACACTTCAATGAACTTAGCACCTGCACTTTTTTCAAGAACC
      M  A  S  A  V  F  Y  T  S  M  N  L  A  P  A  L  F  S  R  T
  61 CATTTCTTGAATTCAATTCCTAAGGACTTTTCTGATGAATTCTTGAATACCCATCATGTT
      H  F  L  N  S  I  P  K  D  F  S  D  E  F  L  N  T  H  H  V
 121 AATTATTACTTACCAACTAAAAAGATTGGATCTTTAAAAAGGTTGACTAGTAAAGTTAAA
      N  Y  Y  L  P  T  K  K  I  G  S  L  K  R  L  T  S  K  V  K
 181 GCTGCTGTGACTGAGGTGAATAGCAAGGTGAATCAAAGTGGTGGTGAGAATTTGAGTGAG
      A  A  V  T  E  V  N  S  K  V  N  Q  S  G  G  E  N  L  S  E
 241 GGGAAGAAGTTGAAGATTCTGGTGGCTGGAGGAGGCATTGGAGGCTTGGTTTTCGCGTTG
      G  K  K  L  K  I  L  V  A  G  G  G  I  G  G  L  V  F  A  L
 301 GCTGCGAGGAGGAAAGGGTTTGAAGTGGTGGTGTTTGAGAGGGATTTGAGTGCTATTAGA
      A  A  R  R  K  G  F  E  V  V  V  F  E  R  D  L  S  A  I  R
 361 GGTGAAGGACAGTATAGAGGACCTATTCAGATACAAAGTAATGCTTTGGCTGCTTTAGAA
      G  E  G  Q  Y  R  G  P  I  Q  I  Q  S  N  A  L  A  A  L  E
 421 GCTATTGATTGGGATGTTGCTGATGATGTTATGAAAGCTGGTTGTATTACTGGTGATAGA
      A  I  D  W  D  V  A  D  D  V  M  K  A  G  C  I  T  G  D  R
 481 ATTAATGGATTGGTTGATGGTGTTTCTGGCAATTGGTACTGCAAGTTTGATACGTTCACG
      I  N  G  L  V  D  G  V  S  G  N  W  Y  C  K  F  D  T  F  T
 541 CCTGCAGCAGAACGAGGCCTTCCAGTCACTAGAGTTATAAGTCGCATGACGTTGCAAAAA
      P  A  A  E  R  G  L  P  V  T  R  V  I  S  R  M  T  L  Q  K
 601 ATTCTGGCTACCGCTGTTGGGGATGATATTATATTGAATGGTAGCAATGTAGTGGACTTT
      I  L  A  T  A  V  G  D  D  I  I  L  N  G  S  N  V  V  D  F
 661 GAGGATGATGGAAATAAGGTTACTGTTATTCTGGAGGATGGACAGCGCTGTGAAGGGGAT
      E  D  D  G  N  K  V  T  V  I  L  E  D  G  Q  R  C  E  G  D
 721 CTTTTGGTTGGCGCTGATGGAATATGGTCAAAGGTACGGAGAAATTTGTTTGGATACACA
      L  L  V  G  A  D  G  I  W  S  K  V  R  R  N  L  F  G  Y  T
 781 GAACCTACATATTCAGGTTACACTTGTTATACAGGGATTGCAGATTTTGTGCCTGCAGAT
      E  P  T  Y  S  G  Y  T  C  Y  T  G  I  A  D  F  V  P  A  D
 841 ATTGATACTGTCGGGTACCGAGTTTTTCTGGGCCACAAACAATACTTTGTTTCTTCAGAC
      I  D  T  V  G  Y  R  V  F  L  G  H  K  Q [Y  F  V  S  S  D]
 901 GTGGGTGGGGGAAAGATGCAATGGTATGCATTTTACAATGAACCTGCTGGTGGCACAGAT
      V  G  G  G  K  M  Q  W  Y  A  F  Y  N  E  P  A  G  G  T  D
 961 AAGGAAAATGGTAAAAAGGAAAGGCTCCTTCAAATATTTGGAGGTTGGTGCGATAATGTT
      K  E  N  G  K  K  E  R  L  L  Q  I  F  G  G  W  C  D  N  V
1021 ATAGATTTGTTAACTGCTACAGACGAAGAAGCAATTCTTCGGCGTGACATATATGACCGG
      I  D  L  L  T  A  T  D  E  E  A  I  L  R  R  D  I  Y  D  R
1081 GAACCAACTTTTAATTGGGGAAAGGGTCGTATAACCTTGCTTGGGGACTCGGTTCATGCT
      E  P  T  F  N  W  G  K  G  R  I  T  L  L  G  D  S  V  H  A
1141 ATGCAGCCAAATTTAGGTCAAGGGGGGTGCATGGCCATTGAGGACAGCTATCAACTAGCA
      M  Q  P  N  L  G  Q  G  G  C  M  A  I  E  D  S  Y  Q  L  A
1201 TTGGAGCTTGATAAAGCGTGTAAGCGGAGTGAGGAATCAGGAAATCCTATTGATATCGAG
      L  E  L  D  K  A  C  K  R  S  E  E  S  G  N  P  I  D  I  E
1261 TCTTCCCTAAGAAGCTATGAGAGTTCCAGGAAGATCCGTGTCTCAGTTATTCATGGACTT
      S  S  L  R  S  Y  E  S  S  R  K  I  R  V  S  V  I  H  G  L
1321 GCTAGAATGGCTGCAATCATGGCCTCAACGTATAAAGCATATTTGGGTGTAGGACTTGGT
      A  R  M  A  A  I  M  A  S  T  Y  K  A  Y  L  G  V  G  L  G
1381 CCGTTGTCGTTCTTGACCAAGTTTAGAATACCACATCCAGGGAGAGTTGGAGGAAGATTT
      P  L  S  F  L  T  K  F  R  I  P  H  P  G  R  V  G  G  R  F
1441 TTTATTGACATCGGGATGCCATTAATGCTGAGTTGGGTACTAGGTGGCAATGGATCAAAC
      F  I  D  I  G  M  P  L  M  L  S  W  V  L  G  G  N  G  S  N
1501 CTTGAAGGGAGACCATTACAGTGCAGGCTTTCTGACAGAGCAAACAGCGATCTCAAAAGA
      L  E  G  R  P  L  Q  C  R  L  S  D  R  A  N  S  D  L  K  R
1561 TGGTTTGAAGACGATGATGCATTGGAGCGTGCTACTAATGGAGAGTGGGTTCTGTTTCCT
      W  F  E  D  D  D  A  L  E  R  A  T  N  G  E  W  V  L  F  P
1621 GTCGGGAATACATCTGCTTCTTCAGAATCAATATTTCTCAGCAAGGATGAGGGAAAACCT
      V  G  N  T  S  A  S  S  E  S  I  F  L  S  K  D  E  G  K  P
1681 TGTATAGTCGGGAGCGTATTGCACCCAAACATTCCAGGAATATCATTTGCCATACCTTCA
      C  I  V  G  S  V  L  H  P  N  I  P  G  I  S  F  A  I  P  S
1741 CCCCAGGTTTCCAACTTGCATGCTAAAATAACATGCAAGAGGGGTGCCTTTTTTGTTACT
      P  Q  V  S  N  L  H  A  K  I  T  C  K  R  G  A  F  F  V  T
1801 GATTTGCGAAGTGAACATGGTACCTATGTGACCGATAACGAGGGCAGACGGTATCGGCTA
      D  L  R  S  E  H  G  T  Y  V  T  D  N  E  G  R  R  Y  R  L
1861 CCCCCCAACTTTCCTACTCGCTTTCATCCATCAGATGTTATCGGATTTGGTTCTGATGAA
      P  P  N  F  P  T  R  F  H  P  S  D  V  I  G  F  G  S  D  E
1921 AAGGTAGCTTTTCGGGTAAAGATTATGAAGTTTCCACCCAAGGTTGCAGAGAAAACCGAA
      K  V  A  F  R  V  K  I  M  K  F  P  P  K  V  A  E  K  T  E
1981 GGAAGTGAAGCTCTCCAAGCAGTATGAGATTTGTCAACTAGACTTTAGATATTGCAAACA
      G  S  E  A  L  Q  A  V  *
     CCAAATTGTATAGAATTATCCTTGAGGCAGCGGACACACAACACTGCTGTTTTGTACAAT
     GTAGGACCAAAGGTATAGCATGCAAGAAAGAACAGGACCCATAGATTTGCTTGTGTATTG +102
```

Figure S1K. The nucleotide sequence and deduced amino acid sequences of HmZEP. An asterisk (*) represents a stop codon.

```
                                                  -3 CAAACCAAAC
   1  ATGATCTCCATCAAAGTGCAACTCTCGACTCTTCTGGATTCGGGTACACCTGGTTTCTAC
       M  I  S  I  K  V  Q  L  S  T  L  L  D  S  G  T  P  G  F  Y
  61  CCGACCCACCCGTTTCTCCAAATCGGTTCTTCCCACCGGAATACCACTCCTATTACCACA
       P  T  H  P  F  L  Q  I  G  S  S  H  R  N  T  T  P  I  T  T
 121  ATCCGGTCACTTTCCAGCCGGAAAACCACCATCCGTGCAACAAACACCGCCATAACAACG
       I  R  S  L  S  S  R  K  T  T  I  R  A  T  N  T  A  I  T  T
 181  GGTTTGGAGAGTAAAGATATAATTGAGAAAGTTAAAATTGTGGGTATTGTTGGAGAAGAC
       G  L  E  S  K  D  I  I  E  K  V  K  I  V  G  I  V  G  E  D
 241  AGTGTGAGTCCTCTCAACTCTGCTTCTTGGTATGATGTTATGCTTCACACTGCCAAGAGA
       S  V  S  P  L  N  S  A  S  W  Y  D  V  M  L  H  T  A  K  R
 301  CTAAAATGGGTTGATGATGGATTTGAAATGCTGGTTTTTACAGATAACATTGAGAAATCT
       L  K  W  V  D  D  G  F  E  M  L  V  F  T  D  N  I  E  K  S
 361  TCTGACAAAACAGTCAAAAATCTTACCAAGGAGCTACATTATGCAAACATATTGGTGCTT
       S  D  K  T  V  K  N  L  T  K  E  L  H  Y  A  N  I  L  V  L
 421  ATTTCTGTAACAAATGAAGAATCAGTAAAATGGATTCAGCAGAACAGTAAAAACATCCCA
       I  S  V  T  N  E  E  S  V  K  W  I  Q  Q  N  S  K  N  I  P
 481  AACATAGTATGTTTCGAGTCATCTCCAGCACTAAGTAACAAGCTAGGAGGGTCATTTATC
       N  I  V  C  F  E  S  S  P  A  L  S  N  K  L  G  G  S  F  I
 541  CAGAGACAAACCAGGGGGGACATTTTCAGCAAACTATCAGAAATTTCCCAGCCGAAGAAA
       Q  R  Q  T  R  G  D  I  F  S  K  L  S  E  I  S  Q  P  K  K
 601  GTATATGAATCGGAGGAAGTTGTTAAAACTATTTACGAGGCTTGGGACCGTTATAACTCG
       V  Y  E  S  E  E  V  V  K  T  I  Y  E  A  W  D  R  Y  N  S
 661  GATGATATAAGATTTTCTTTGCTAGTTATAATCAATGCTTATATAAGGCCAGTTTCAATC
       D  D  I  R  F  S  L  L  V  I  I  N  A  Y  I  R  P  V  S  I
 721  TTGAAAAACCTTAGAGCACAAGGTTTCTCCACGCTTAGTTGCATGGTGAAAAACTGTGGG
       L  K  N  L  R  A  Q  G  F  S  T  L  S  C  M  V  K  N  C  G
 781  CCTCAGGTCTTGAACTGTCTCTTAGATCCTAACTGTAGGAAGGCTCTTCAGTGCCTGAAT
       P  Q  V  L  N  C  L  L  D  P  N  C  R  K  A  L  Q  C  L  N
 841  CAATGCAGTCCAGTAGATCAGGTATGTAACTATCAATGTATTGCTTCGTATGAGAGTCCG
       Q  C  S  P  V  D  Q  V  C  N  Y  Q  C  I  A  S  Y  E  S  P
 901  GCATTAGAAGAATTTTCTCTATGTGTACTACAAAAGAACAATTGCCTTGAGTTGGAGGCC
       A  L  E  E  F  S  L  C  V  L  Q  K  N  N  C  L  E  L  E  A
 961  AAGATCCCTGAAAAACCTTATGTCCCACCAATGAATAGGTTTCAAGGGAAAGACCTGAGT
       K  I  P  E  K  P  Y  V  P  P  M  N  R  F  Q  G  K  D  L  S
1021  CATGAAATTGCTGAGGATCTTTTTGTGGGTTGGTTAGGGAATTTGGACTGGAGTTGGCGT
       H  E  I  A  E  D  L  F  V  G  W  L  G  N  L  D  W  S  W  R
1081  GTTGTAGCAGGACAGAACCCCGCCTACGATCAGTTTCCTTGTCAGTACCAGCTCTTCTAC
       V  V  A  G  Q  N  P  A  Y  D  Q  F  P  C  Q  Y  Q  L  F  Y
1141  AGAGGTAAGGCAAGGGGGTCATTCTGGTATGAGCCAGTTTTTCAGGTAAAAACCTTAGAT
       R  G  K  A  R  G  S  F  W  Y  E  P  V  F  Q  V  K  T  L  D
1201  GGGGAGTTGGTTTGGAGGCGGCGAAAATATCGAGTTAAAAGAAGCAAAGTGCCCGGGACA
       G  E  L  V  W  R  R  R  K  Y  R  V  K  R  S  K  V  P  G  T
1261  TTTAGTTTTAGTGTTTTGGATAATGGGGTAGTTTCAAATGAGTTTTGGACTATTGTAGAT
       F  S  F  S  V  L  D  N  G  V  V  S  N  E  F  W  T  I  V  D
1321  GTTGCTGCTGATTTTAGCTGGGGTTTGTTTCACTATAGTGGAGCTGCTCGAGTTGCCGGA
       V  A  A  D  F  S  W  G  L  F  H  Y  S  G  A  A  R  V  A  G
1381  CAATCTTACACTGGAGCAGTTCTTGTTAGTCCGAATGGCGAATACCCAGATGAGAGGGAG
       Q  S  Y  T  G  A  V  L  V  S  P  N  G  E  Y  P  D  E  R  E
1441  CGAGATAAATTAGTTTGTGCGCTAGACAGATGCCACATCAAAGAGTGGGAGTTGTTCGAT
       R  D  K  L  V  C  A  L  D  R  C  H  I  K  E  W  E  L  F  D
1501  GTTGACAATTGTTCGTGCAAAGATCCACCACTGGGCATTCCCCAAGGTTCGAGTTTGCAC
       V  D  N  C  S  C  K  D  P  P  L  G  I  P  Q  G  S  S  L  H
1561  AAGATCATCAAAGTTGAGGATCATCTAAAAGGCTCTTTCAAATGATCTTGGGGGGAATTC
       K  I  I  K  V  E  D  H  L  K  G  S  F  K  *
      TAAAGCTGGAGTGTTGTTGGTTGTTCCTGATAAAGTCATACTTACTGTTTGTAAAGCACA
      TGTGTGGTTAAAGTTAACAAATGTGAGAAATGAGGGAATTGTATTATTATGTAAGTAACA +343
```

Figure S1L. The nucleotide sequence and deduced amino acid sequences of HmVDE. An asterisk (*) represents a stop codon.

```
                                         -3 ACTTAATAACAAGTTACAAGAAAA
   1 ATGGATGCTTTCTCATCCTCTTTTCTCTCAACAACTTACATTCTTGGTCCAAAACCACCA
      M  D  A  F  S  S  S  F  L  S  T  T  Y  I  L  G  P  K  P  P
  61 TCGTCGACACAACTATCCCATTTACACCACCGTATCTCCGCCGTGAGAATCGAAGAAAGA
      S  S  T  Q  L  S  H  L  H  H  R  I  S  A  V  R  I  E  E  R
 121 CCACCAACCACCTCTACTAACCCTCCAAATTATAAACAAAACACTAAAACCACCACTCCT
      P  P  T  T  S  T  N  P  P  N  Y  K  Q  N  T  K  T  T  T  P
 181 AGAAAATCATCCCCACCACCGAGACGGTCACCACCGCCGCCGCAACAATCTTTTCCAGCG
      R  K  S  S  P  P  P  R  R  S  P  P  P  P  Q  Q  S  F  P  A
 241 ACCCTTTTTAGTGCATTAGACAACATTGTAAACACCTACATAGACCCACCTAGCAAACCT
      T  L  F  S  A  L  D  N  I  V  N  T  Y  I  D  P  P  S  K  P
 301 TCGGTGGACCCCAAGTTTACACTCTCCGATAACTTCGCACCTGTGGATGAGCTTCCACCG
      S  V  D  P  K  F  T  L  S  D  N  F  A  P  V  D  E  L  P  P
 361 ACAGAATGTGAAGTTATCCACGGCGTCCTACCACCGTGCCTCGACGGCGCGTACATTCGA
      T  E  C  E  V  I  H  G  V  L  P  P  C  L  D  G  A  Y  I  R
 421 AATGGCCCAAACCCACAATTTCTGCCACGTGGACCGTACCACCTTTTTGACGGCGATGGC
      N  G  P  N  P  Q  F  L  P  R  G  P  Y  H  L  F  D  G  D  G
 481 ATGCTCCACGCGCTTAAAATTTCAAACGGACGCGCTACTCTTTGTAGCCGTTATGTAAAA
      M  L  H  A  L  K  I  S  N  G  R  A  T  L  C  S  R  Y  V  K
 541 ACCTACAAGTTTACAGTAGAACAAGCAAACGGATCACAAGTGATCCCGAATGTGTTCTCG
      T  Y  K  F  T  V  E  Q  A  N  G  S  Q  V  I  P  N  V  F  S
 601 GGTTTTAATGGTCTTACTGCCGCGGCGGCCCGTGGGGCGGTGTCGGCTGCAAGAATGATC
      G  F  N  G  L  T  A  A  A  A  R  G  A  V  S  A  A  R  M  I
 661 ACGGGACAATTTAACCCTGCTAATGGAATCGGCAATGCAAACACTAGCTTGGCATTCTTC
      T  G  Q  F  N  P  A  N  G  I  G  N  A  N  T  S  L  A  F  F
 721 GGTAACCGTCTTTTCGCTCTCGGAGAATCTGATCTTCCGTACACCATTAAAATCTCAGAA
      G  N  R  L  F  A  L  G  E  S  D  L  P  Y  T  I  K  I  S  E
 781 GATGGTGAAATTACGACATTAGGCCGTCATGATTTTGCTGGAAAGCTTAACATGAGCATG
      D  G  E  I  T  T  L  G  R  H  D  F  A  G  K  L  N  M  S  M
 841 ACAGCGCACCCTAAAATTGATCCCAAGACTGGTGAGGCTTTTGCATTTCGGTACGGACCA
      T  A  H  P  K  I  D  P  K  T  G  E  A  F  A  F  R  Y  G  P
 901 ATGCCTCCGTACTTGACGTTCTTTCGATTTGATTCGGAAGGAAATAAATCGCAAGATGTT
      M  P  P  Y  L  T  F  F  R  F  D  S  E  G  N  K  S  Q  D  V
 961 CCGATATTCTCCATGCAGACACCATCGTTTTTGCATGATTTTGCAATTTCACAAAAATAC
      P  I  F  S  M  Q  T  P  S  F  L  H  D  F  A  I  S  Q  K  Y
1021 GCAATTTTCACGGAAATTCAATTAGGTATGAATAATCCTGTAGATATGATTTTTAACGGC
      A  I  F  T  E  I  Q  L  G  M  N  N  P  V  D  M  I  F  N  G
1081 GGACCACCTGTGGGTGCGAATTCGGAGAAGGTTCCGAGAATTGGAATAATTCCGAGGTAT
      G  P  P  V  G  A  N  S  E  K  V  P  R  I  G  I  I  P  R  Y
1141 GCTAAAGATGAATCAGAAATGAAGTGGTTTGAAGTGCCGGGATTTAATTTAATGCATGCG
      A  K  D  E  S  E  M  K  W  F  E  V  P  G  F  N  L  M  H  A
1201 ATAAATGCATGGGATGAAGATGGTGGAGATACGGTGGTGATGCTTGCGCCAAATTTAATG
      I  N  A  W  D  E  D  G  G  D  T  V  V  M  L  A  P  N  L  M
1261 TCGGTGGAACATACGATGGAGAGGCTGGATTTGATTCATGCGGTGGTCGAGAAGGTGAAG
      S  V  E  H  T  M  E  R  L  D  L  I  H  A  V  V  E  K  V  K
1321 ATTAATTTAAAAACAGGGATTGTTTCTAGGTTTCCACTTTCTACCCGGAATTTGGATTTT
      I  N  L  K  T  G  I  V  S  R  F  P  L  S  T  R  N  L  D  F
1381 GCAGTTGTTAATCCGTCTTACATCGGCCAAAACAACAGGTATGTTTATGCAGCAATCGGA
      A  V  V  N  P  S  Y  I  G  Q  N  N  R  Y  V  Y  A  A  I  G
1441 GATCCTATGCCAAAGATTAGCGGAGTGGTAAAGCTAGACGTAACAGTTTCGGACAATGAC
      D  P  M  P  K  I  S  G  V  V  K  L  D  V  T  V  S  D  N  D
1501 CGACGAGATTGCATAGTTGCTAGTCGAATGTTCGGGCCAAAATGTTTTGGAGGTGAACCT
      R  R  D  C  I  V  A  S  R  M  F  G  P  K  C  F  G  G  E  P
1561 TTTTTTGTTGCAAAGGATCCCGAAAATCCGGAGGCAGCAGAGGACGACGGCTACGTTGTA
      F  F  V  A  K  D  P  E  N  P  E  A  A  E  D  D  G  Y  V  V
1621 TCATACGTTCATGATGAGAACACTGGAGAATCAAGATTTATAGTGATGGATGCACAGTCT
      S  Y  V  H  D  E  N  T  G  E  S  R  F  I  V  M  D  A  Q  S
1681 CCAGATCTTGAAATTGTGGCTGCCGTGAAGTTGCCCCGGCGAGTACCTTACGGGTTTCAC
      P  D  L  E  I  V  A  A  V  K  L  P  R  R  V  P  Y  G  F  H
1741 GGGCTTTTTGTGAAGCAAAGTCAACTAAACATGATATAACATAAAAGTGTACAAATTAGA
      G  L  F  V  K  Q  S  Q  L  N  M  I  *
     TGAAATTATAATATACAGAATACTGTATTAGGATATTGTAGGGTGTACTACATTAATAGT
     TACTAATGTACAAATTTTAAATAGTTGTATATTTTTATTTTTTAATAGGAATTTATTAAA +31
```

Figure S1M. The nucleotide sequence and deduced amino acid sequences of HmCCD. An asterisk (*) represents a stop codon.

```
           -3 TACATTAACTCATCCCATCTCTCTCCCTCTCCCCCAAAAATTTCTC
      ATTTCTTACCACCACTAACACTACCAAACTCTAACTTCTCAAAATAGATACACAATACTC
   1  ATGGCTGCTTCTGCCGCTGCTACAAGTACAAGGCCTAAACCCAATACTTACACAAAAACC
       M  A  A  S  A  A  A  T  S  T  R  P  K  P  N  T  Y  T  K  T
  61  ATTGTAAATTCTACTATACAATCACCTTCTATACTTCATTTCCCTAAACAATCCCACAAA
       I  V  N  S  T  I  Q  S  P  S  I  L  H  F  P  K  Q  S  H  K
 121  AAATATCAAACTCCTTCAATCCCCACCACCATTAAACACCAAGATTCTCCAACCCCTCAA
       K  Y  Q  T  P  S  I  P  T  T  I  K  H  Q  D  S  P  T  P  Q
 181  TGGAATTTCCTACAAAAAGCAGCTTCAATGGCCTTAGACGCTGTCGAAAATTTAATCATT
       W  N  F  L  Q  K  A  A  S  M  A  L  D  A  V  E  N  L  I  I
 241  TCACAAGAACGCAACCACCCTTTACCAAAAACAGCTGACCCGCAAGTACAAATCTCTGGA
       S  Q  E  R  N  H  P  L  P  K  T  A  D  P  Q  V  Q  I  S  G
 301  AACTTTGCTCCTGTACAAGAACAACCAGTCCATCATCACCTCCCTGTCACTGGAAAAATA
       N  F  A  P  V  Q  E  Q  P  V  H  H  H  L  P  V  T  G  K  I
 361  CCCGAATGCATTTCCGGTGTCTACGTTCGAAACGGGGCTAATCCCCATTTCGAACCAACC
       P  E  C  I  S  G  V  Y  V  R  N  G  A  N  P  H  F  E  P  T
 421  GGGGGGCACCATTTTTTTGACGGGGATGGCATGGTCCATGCTGTCATGTTCAAAGATGGT
       G  G  H  H  F  F  D  G  D  G  M  V  H  A  V  M  F  K  D  G
 481  GCAGCTAGCTATGCTTGCAGGTATACTGAAACTGAAAGACTAATCCAGGAGCGTAATTTG
       A  A  S  Y  A  C  R  Y  T  E  T  E  R  L  I  Q  E  R  N  L
 541  GGTCGAACTGTTTTTCCGAAAGCCATTGGTGAGCTCCATGGTCATTCAGGGATAGCTCGG
       G  R  T  V  F  P  K  A  I  G  E  L  H  G  H  S  G  I  A  R
 601  CTTGCTCTGTTTTATGCTCGTGGCCTTTTTGGACTTGTTGATCATTCTCAGGGTACTGGA
       L  A  L  F  Y  A  R  G  L  F  G  L  V  D  H  S  Q  G  T  G
 661  GTAGCTAATGCTGGCTTGGTTTATTTTAACAACAGGTTATTAGCTATGTCTGAAGATGAC
       V  A  N  A  G  L  V  Y  F  N  N  R  L  L  A  M  S  E  D  D
 721  TTGCCTTATCATGTTCAAATCACTTGTTCAGGTGATCTTAAAACTGTTGAGAGATATAGT
       L  P  Y  H  V  Q  I  T  C  S  G  D  L  K  T  V  E  R  Y  S
 781  TTTGATGGTCAGTTGGATTCTACTATGATTGCTCATCCCAAGATTGATCCCGAGACTAAT
       F  D  G  Q  L  D  S  T  M  I  A  H  P  K  I  D  P  E  T  N
 841  GAGCTTATAGCATTAACTTATAATGTTATTCAGAAGCCGTATCTCAAGTATTTTCGGGTT
       E  L  I  A  L  T  Y  N  V  I  Q  K  P  Y  L  K  Y  F  R  V
 901  TCGCCTAAAGGAGTCAAGTCATTAGATGTTGATATTGAATTAGCTGATCCTACTATGATG
       S  P  K  G  V  K  S  L  D  V  D  I  E  L  A  D  P  T  M  M
 961  CATGATTTCGCAATTACGGAGAATTTTGTGGTGATTCCTGATCAGCAAGTTGTTTTCAAG
       H  D  F  A  I  T  E  N  F  V  V  I  P  D  Q  Q  V  V  F  K
1021  ATTTCGGAAATGATACAAGGTGGATCGCCTGTTGTGTACGATAAGGAGAAAGTGTCACGT
       I  S  E  M  I  Q  G  G  S  P  V  V  Y  D  K  E  K  V  S  R
1081  TTTGGTGTTTTAGACAAGTATGCGAAAGATGGGTCGGGAATTAAGTGGGTTGAGGTTCCG
       F  G  V  L  D  K  Y  A  K  D  G  S  G  I  K  W  V  E  V  P
1141  GATTGTTTTTGTTTTCATTTGTGGAATGCTTGGGAGGATAAAGAGAGTGATGAAATTGTG
       D  C  F  C  F  H  L  W  N  A  W  E  D  K  E  S  D  E  I  V
1201  GTGATAGGCTCTTGTATGACTCCACCTGATTCAATTTTTAATGAATGTGATCAAGGGCTA
       V  I  G  S  C  M  T  P  P  D  S  I  F  N  E  C  D  Q  G  L
1261  GAAAGTGTTTTGTCTGAAATTAGGCTCAACACCAAGACAGGGAAATCCACTCGTAGGGCA
       E  S  V  L  S  E  I  R  L  N  T  K  T  G  K  S  T  R  R  A
1321  ATCATTTCACCCCAAGATCAAGTGAATTTAGAGGCAGGCATGGTGAATAGAAACAAGTTG
       I  I  S  P  Q  D  Q  V  N  L  E  A  G  M  V  N  R  N  K  L
1381  GGAAGAAAGACACAATTTGCGTACTTGGCTATTGCTGAGCCGTGGCCTAAAGTGTGTGGT
       G  R  K  T  Q  F  A  Y  L  A  I  A  E  P  W  P  K  V  C  G
1441  TTTGCCAAAGTAGACCTTTTTAATGGAAAAATCACAAAGTTGTTTTATGGGGATAACAAA
       F  A  K  V  D  L  F  N  G  K  I  T  K  L  F  Y  G  D  N  K
1501  TATGGAGGTGAGCCTCTATTCCTTCCAAGAAATCCCAATTCGGACAAGGAAGATGATGGG
       Y  G  G  E  P  L  F  L  P  R  N  P  N  S  D  K  E  D  D  G
1561  TACATATTAGCTTTTGTTCACGATGAGAAGGCGTGGAAATCGGAGCTTCAAATCGTTAAT
       Y  I  L  A  F  V  H  D  E  K  A  W  K  S  E  L  Q  I  V  N
1621  GCCGTGACTTTAGAGTTGGAGGCTACTGTTAAGCTTCCGTCGAGGGTGCCATATGGTTTT
       A  V  T  L  E  L  E  A  T  V  K  L  P  S  R  V  P  Y  G  F
1681  CATGGCACTTTTATAAGTGAAAAAGATATAGAAAAACAGGCCTAATCTACGAGATTAGTT
       H  G  T  F  I  S  E  K  D  I  E  K  Q  A  *
      ACAGTTAGTTGATTAGTTTGATTGTGATTGGTTGCAGCCGAGATTGACCAGAGGGATTGA
      TCGCTTATTTGTCCCCGGAAATGTACGAGCTGTTTAAAGTATGAACCTTGTTTGTGTATT +462
```

Figure S1N. The nucleotide sequence and deduced amino acid sequences of HmNCED. An asterisk (*) represents a stop codon.

```
 -55  AGATAGATAATACATTATTTTCTGTTTCTACCTGCTGCCTTTCCTACTCTTCAACTCTCA
      TGTGTTTCTTCTGACCTATATTATATAAGACTGTAGATTTTGTAACTTTATAATTCATAC
   1  ATGGAGGAGTTAAAAAGAGTTGATGGTGCTAGTGGTAGTAATAAAAATGATGACGATCTT
       M  E  E  L  K  R  V  D  G  A  S  G  S  N  K  N  D  D  D  L
  61  GTTGTATTTGCAGTAAATGGGAAACGATATGAGGTTTCATGTCACCCTTCTACTAGTCTT
       V  V  F  A  V  N  G  K  R  Y  E  V  S  C  H  P  S  T  S  L
 121  GTCGAATTCTTGCGTTGTCGTACTCCTTTCAAGAGTGTCAAGCTCAGCTGTGGTGAAGGT
       V  E  F  L  R  C  R  T  P  F  K  S  V  K  L  S  C  G  E  G
 181  GGTTGCGGTGCTTGTGTTGTTTTACTGTCGAATTATGACCCTGTGCTTAAGACAGTAAAA
       G  C  G  A  C  V  V  L  L  S  N  Y  D  P  V  L  K  T  V  K
 241  AGTTACACTGTGAGTTCATGTCTTACCCTCGTTCACAGTGTAAATTATTGTTCGATTACA
       S  Y  T  V  S  S  C  L  T  L  V  H  S  V  N  Y  C  S  I  T
 301  ACAACTGAAGGACTTGGAAATAGCAGGGATGGTTTTCACCCAATTCATAAAAGATTTTCT
       T  T  E  G  L  G  N  S  R  D  G  F  H  P  I  H  K  R  F  S
 361  GGTTTTCATGCTTCTCAATGTGGTTTCTGCACGCCTGGAATGTGCATTTCACTGTTCTCA
       G  F  H  A  S  Q  C  G  F  C  T  P  G  M  C  I  S  L  F  S
 421  GCACTTGTCAATGCCGAAGAGAAGCAGAGGCCAGATCCCCCTACAGGATTTTCTAAACTC
       A  L  V  N  A  E  E  K  Q  R  P  D  P  P  T  G  F  S  K  L
 481  ACTGTTTCGGAAGCTGAAAAGGCCATATCAGGGAATCTCTGTAGATGTACTGGATATCGA
       T  V  S  E  A  E  K  A  I  S  G  N  L  C  R  C  T  G  Y  R
 541  CCTATTGCAGACGCTTGTAAAAGTTTTGCTGCTGATGTTGATCTGGAGGATTTGGGAATA
       P  I  A  D  A  C  K  S  F  A  A  D  V  D  L  E  D  L  G  I
 601  AATTCTTTCTGGAGAGAGGGAGAGAGTAATGGAGATAAACTTAGTAAATTACCTTGTTAT
       N  S  F  W  R  E  G  E  S  N  G  D  K  L  S  K  L  P  C  Y
 661  AATCCGAAAGATGATGTCTGTACATATCCCGAATTCTTGAAAAGCGAATTGGAGTCTGCA
       N  P  K  D  D  V  C  T  Y  P  E  F  L  K  S  E  L  E  S  A
 721  ATGTATTTGAGCCCTCACAAAAAATCGTGGTACAGTCCTCTTACTCTTGAGGAGCTCCAG
       M  Y  L  S  P  H  K  K  S  W  Y  S  P  L  T  L  E  E  L  Q
 781  AATTTCACTGACCTGAAATTGTCTACTACTCGGACTAAGTTAGTTGCTGGTAACACAGGA
       N  F  T  D  L  K  L  S  T  T  R  T  K  L  V  A  G  N  T  G
 841  ATTGGTTATTACAAAGAACTCAATCAGTATGAGAATTACATTAACTTGAGGAATGTCTCT
       I  G  Y  Y  K  E  L  N  Q  Y  E  N  Y  I  N  L  R  N  V  S
 901  GAGCTTTCATCAATTTGTAGAAATGAGACAGGGATAGTAATTGGAGCAACTGTCACAATA
       E  L  S  S  I  C  R  N  E  T  G  I  V  I  G  A  T  V  T  I
 961  TCTAAAGCTATCTTAGCTTTGAAGGAAAAAGTAGAAGGTGGCTTATGTTCAGATGGGGAA
       S  K  A  I  L  A  L  K  E  K  V  E  G  G  L  C  S  D  G  E
1021  TTGACATTTACAAAAATTGCTGAGCATTTAGAGAAGATTGCTTCAGAGCCTGTTCGTAAC
       L  T  F  T  K  I  A  E  H  L  E  K  I  A  S  E  P  V  R  N
1081  TTAGCTAGTGTAGGGGGAAATGTGGTTATGGCACAAAAAAATCATTTCCCATCAGACATT
       L  A  S  V  G  G  N  V  V  M  A  Q  K  N  H  F  P  S  D  I
1141  GTTACAGTACTTCTTGCTGTAAGTTCCACAGTTGATTTACTAATAGATACAAAACGTGAA
       V  T  V  L  L  A  V  S  S  T  V  D  L  L  I  D  T  K  R  E
1201  ATTCTTACACTGGAAGAATTCTTGTCAAAGCCACCATTGGATCATAGAGGGGTACTTTTA
       I  L  T  L  E  E  F  L  S  K  P  P  L  D  H  R  G  V  L  L
1261  AGTATCAAAATTCCCTTTTCGAGCTCATTTAGAACTAGCTTTTCTACTGAAACGAGTAAG
       S  I  K  I  P  F  S  S  S  F  R  T  S  F  S  T  E  T  S  K
1321  CTGGTGTTTGAAACTTATCGCGCTGCTCCAAGGCCGCTTGGAAATGCACTACCTTACCTA
       L  V  F  E  T  Y  R  A  A  P  R  P  L  G  N  A  L  P  Y  L
1381  AATGCTGCATTTTTAGCCATTATTTCACCAAATAGAATTGGAGTCGTCATTAATAAGATT
       N  A  A  F  L  A  I  I  S  P  N  R  I  G  V  V  I  N  K  I
1441  CAGTTGGTTTTTGGTGCTTTTGGAGCTAAACATGCCAAGAGAGCAAGAGAGGCTGAAGAA
       Q  L  V  F  G  A  F  G  A  K  H  A  K  R  A  R  E  A  E  E
1501  TACTTAGCTGGTAAAATTTTAAGTTTGAATGTGCTTTACGAGGCTATTAACATAATTAGA
       Y  L  A  G  K  I  L  S  L  N  V  L  Y  E  A  I  N  I  I  R
1561  GCCACTGTCATATCTGAAGATGGAACTTCAGATGCTATGTACCGATCAAGCTTGGTTGTC
       A  T  V  I  S  E  D  G  T  S  D  A  M  Y  R  S  S  L  V  V
1621  AGTTATCTTTTTGAGTTTCTATATCCTTTGTTTAATGGCGTTGTTTTGGTTTCCAATGGT
       S  Y  L  F  E  F  L  Y  P  L  F  N  G  V  V  L  V  S  N  G
1681  TTGTCTACTCAGAACTCTGCTAACAACTTAGCTGAAGACAACCTGTCAAATGATGCTAAG
       L  S  T  Q  N  S  A  N  N  L  A  E  D  N  L  S  N  D  A  K
1741  AAACCAGCACTATTGTCGTCTGGAAAGCAGCTGGTGACAACAAGCAGAGATTACTATCCG
       K  P  A  L  L  S  S  G  K  Q  L  V  T  T  S  R  D  Y  Y  P
1801  GTTGGTCAACCAATTGTTAAATCTGGTGCTTCTATTCAAGCTTCTGGCGAAGCTGTTTAT
       V  G  Q  P  I  V  K  S  G  A  S  I  Q  A  S  G  E  A  V  Y
1861  GTAGATGACATTCCTTCACCGTCAAACTGCCTACATGGTGCATTTATCTATAGCACAAAG
       V  D  D  I  P  S  P  S  N  C  L  H  G  A  F  I  Y  S  T  K
1921  CCTTTGGCAAGGGTTAAGAATGTACGATTTATGAATAAGTCACCACCAGATGGAGTAACT
       P  L  A  R  V  K  N  V  R  F  M  N  K  S  P  P  D  G  V  T
1981  GCTGTTATAACTTATAATGATATTCCAAGTGGAGGGGAGAACATAGGTTCTAAAACCATA
       A  V  I  T  Y  N  D  I  P  S  G  G  E  N  I  G  S  K  T  I
2041  TTTGGTACAGAACCATTGTTTGCTGATGACATTACCAGGTGTGCTGGCCAGTGTCTTGCT
       F  G  T  E  P  L  F  A  D  D  I  T  R  C  A  G  Q  C  L  A
2101  CTTGTGGCTGCAGAGACACAGAAGCTTGCAGATAGGGCTGCATCTCTGTCCATCGTTGAT
       L  V  A  A  E  T  Q  K  L  A  D  R  A  A  S  L  S  I  V  D
2161  TATGATACTGATAATTTAGATCCACCGATATTAACTCTTGAAGAGGCTGTTGAGAAATCG
       Y  D  T  D  N  L  D  P  P  I  L  T  L  E  E  A  V  E  K  S
2221  AGTTTCTTCGAGGTTCCTCCCTTCTTATATCCTGCACAAATTGGTGACTTCTCGAAAGGC
       S  F  F  E  V  P  P  F  L  Y  P  A  Q  I  G  D  F  S  K  G
2281  ATGGCTGAAGCTGACCACAAAATAAACTCTCAGATCAAACTTGGTTCTCAATATTATTTT
       M  A  E  A  D  H  K  I  N  S  Q  I  K  L  G  S  Q  Y  Y  F
2341  TATATGGAAACACAAACTGCCCTTGCTATCCCAGATGAAGACAGTTGCATGGTGGTGTAT
       Y  M  E  T  Q  T  A  L  A  I  P  D  E  D  S  C  M  V  V  Y
2401  AGTTCTATTCAGTGTCCTGAGTCCGTACACTCGGTAATTGCAAGATGTCTTGGTATTCCA
       S  S  I  Q  C  P  E  S  V  H  S  V  I  A  R  C  L  G  I  P
2461  GAACATAATGTTCGTGTTATTACGAGAAGGGTTGGAGGGGGCTTTGGCGGAAAGGCGATA
       E  H  N  V  R  V  I  T  R  R  V  G  G  G  F  G  G  K  A  I
2521  AAGGCCATGCCTGTAGCAACAGCATGTGCACTTGCAGCACACAAACTGCGCCGTCCTGTA
       K  A  M  P  V  A  T  A  C  A  L  A  A  H  K  L  R  R  P  V
2581  CGTATGTATCTTAATCGTAAGACTGACATGATTATGGCGGGAGGAAGACATCCCATGAAG
       R  M  Y  L  N  R  K  T  D  M  I  M  A  G  G  R  H  P  M  K
2641  ATAAGTTATGATGTTGGGTTCAAATCCGATGGAAAGATTACAGCCCTACACCTATATATT
       I  S  Y  D  V  G  F  K  S  D  G  K  I  T  A  L  H  L  Y  I
2701  TTGATCAATGCAGGGATATCTGCAGATATAAGCCCTATAATGCCAAGTAACATGTTGGGC
       L  I  N  A  G  I  S  A  D  I  S  P  I  M  P  S  N  M  L  G
2761  GTGCTTAAGAGATATGATTGGGGAGCTTTAAGTTTTGACATTAAAGTATGCAAGACAAAC
       V  L  K  R  Y  D  W  G  A  L  S  F  D  I  K  V  C  K  T  N
2821  CATTCCAATAAATCTGCAATGCGAGCCCCTGGAGAGGTGCAGCCATCTTTTATTGCGGAA
       H  S  N  K  S  A  M  R  A  P  G  E  V  Q  P  S  F  I  A  E
2881  GCTGTAATGGAACATGTTGCATCTATACTTTCCATGGAAGTAGATTCTGTTAGATACAGG
       A  V  M  E  H  V  A  S  I  L  S  M  E  V  D  S  V  R  Y  R
2941  AACCTTCACACATTTGATAGTCTCAATTTATTCTATAAGCATAGTGCAGGAGAACTCGTG
       N  L  H  T  F  D  S  L  N  L  F  Y  K  H  S  A  G  E  L  V
3001  GAGTATACTCTGCCGTCAATTTGGGACAAAGTACAGATATCATCAAATTTTAGCCAAAGA
       E  Y  T  L  P  S  I  W  D  K  V  Q  I  S  S  N  F  S  Q  R
3061  ACTAAAAAAATACAGCAGTTTAATCAAAAGAATTTATGGAAGAAAAAAGGAATTTCTCGA
       T  K  K  I  Q  Q  F  N  Q  K  N  L  W  K  K  K  G  I  S  R
3121  GTTCCCATTGTGCATGAAGTCACCGTGAGACCTACCCCAGGGAAAGTGAGCGTTCTTTCG
       V  P  I  V  H  E  V  T  V  R  P  T  P  G  K  V  S  V  L  S
3181  GATGGATCCATTGTTGTAGAAGTTGGTGGGATTGAGCTTGGGCAGGGGCTCTGGACGAAG
       D  G  S  I  V  V  E  V  G  G  I  E  L  G  Q  G  L  W  T  K
3241  GTAAAACAAATGACTGCATATGCACTTGGTTCAATTCAATGTGATGGAATTGAAAACCTC
       V  K  Q  M  T  A  Y  A  L  G  S  I  Q  C  D  G  I  E  N  L
3301  GTGGATAAGGTACGGATTGTACAAGCAGATACATTGAGCTTAATTCAAGGCGGGTTCACA
       V  D  K  V  R  I  V  Q  A  D  T  L  S  L  I  Q  G  G  F  T
3361  GCTGGAAGCACGACATCTGAATCAAGCTGTGAAGCAGTAAGAATTTGCTGCAACATTTTG
       A  G  S  T  T  S  E  S  S  C  E  A  V  R  I  C  C  N  I  L
3421  ATTGAACGACTTTCCCCTCTAAAGGAAAAGTTGCAGGCTCAAATGACTTCTTTGACCTGG
       I  E  R  L  S  P  L  K  E  K  L  Q  A  Q  M  T  S  L  T  W
3481  AATACATTAGTTCTTCAGGCATATCGGCAAGCTGTGAACTTGTCAGCAAGTTCTTACTTT
       N  T  L  V  L  Q  A  Y  R  Q  A  V  N  L  S  A  S  S  Y  F
3541  GTGCCTGACTTCAGTTCCATGCAATACTTGAATTATGGTGCTGCAGTAAGTGAGGTGGAG
       V  P  D  F  S  S  M  Q  Y  L  N  Y  G  A  A  V  S  E  V  E
3601  ATAAATACTCTGACCGGAGAAGCTACAATTCTTCGCTCAGATATCATCTATGATTGTGGA
       I  N  T  L  T  G  E  A  T  I  L  R  S  D  I  I  Y  D  C  G
3661  CAGAGCTTGAACCCTGCTGTAGATTTGGGACAGATAGAGGGGGCTTTTGTTCAAGGAATT
       Q  S  L  N  P  A  V  D  L  G  Q  I  E  G  A  F  V  Q  G  I
3721  GGATTCTTCATGTCTGAAGAGTACCTTACTAACTCAGATGGATTGGTGATTGCGGATAGC
       G  F  F  M  S  E  E  Y  L  T  N  S  D  G  L  V  I  A  D  S
3781  ACATGGACGTACAAGATACCAACAATTGATACAATCCCAAAGCAGTTTAATGTCGAGGTA
       T  W  T  Y  K  I  P  T  I  D  T  I  P  K  Q  F  N  V  E  V
3841  CTCAACAGCGAACATCACAAAAACCGTGTTCTTTCATCTAAAGCTTCTGGTGAGCCACCT
       L  N  S  E  H  H  K  N  R  V  L  S  S  K  A  S  G  E  P  P
3901  TTACTTCTGGCAGTTTCAGTACACTGTGCAACAAGGGCAGCCATAAAGGAAGCTCGAAAA
       L  L  L  A  V  S  V  H  C  A  T  R  A  A  I  K  E  A  R  K
3961  CAGCTCTGTTCTTGGAAAAATTCATCCGAAGAGTGCAATTTATCATTCCAGTTAGATGTC
       Q  L  C  S  W  K  N  S  S  E  E  C  N  L  S  F  Q  L  D  V
4021  CCAGCTACCATGCCTGTTGTAAAGCGACTGTGTGGAATGGATTATGTGGAGTCGTACTTG
       P  A  T  M  P  V  V  K  R  L  C  G  M  D  Y  V  E  S  Y  L
4081  AAGCACTTGCTTTCTTGTCAAGAAAGCAAACAGTCTTAATGTTTTTCTTTCTGTATGTCG
       K  H  L  L  S  C  Q  E  S  K  Q  S  *
      GGATGAGAATTGAATTTTCCATCCACCCCAATGAGAATAGTAGTTATACATGCATATTAG
      CTTGTAAGGTGAAAATTTAAAATTAGGCAATAAAACTACATGAGATGCCTCGTGGATGAA +74
```

Figure S1O. The nucleotide sequence and deduced amino acid sequences of HmAO. An asterisk (*) represents a stop codon.

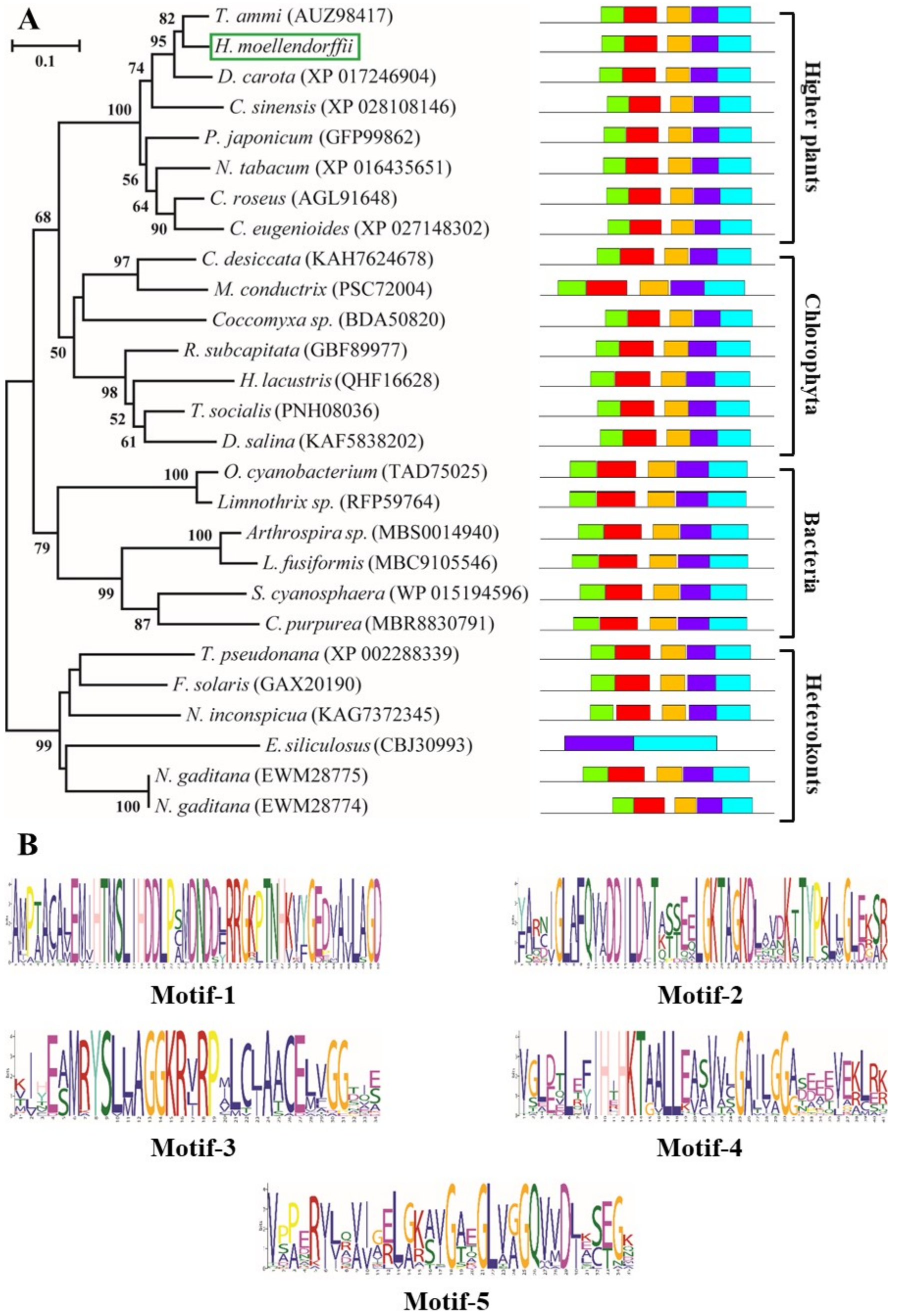

Figure S2A: Phylogeny of deduced HmGGPS amino acid sequences along with other GGPS sequences. The tree was constructed using MEGA 7 Kumar et al. [100] on the basis of the Neighbor-Joining method Saitou and Nei [101]. Bootstrap values on the nodes indicate the number of times each group occurred within 1000 replicates Felsenstein [102]. On the right side, the analysis of conserved protein motifs using MEME software is presented, showing motifs that are ordered in colored boxes corresponding to GGPS sequence in the phylogenetic tree. Other protein sequences obtained from NCBI database as well as their accession numbers are provided in the bracket. (B) Conserved protein motif 1-5 present in the variable region of HmGGPS gene. The height of the letters denotes the degree of conservation.

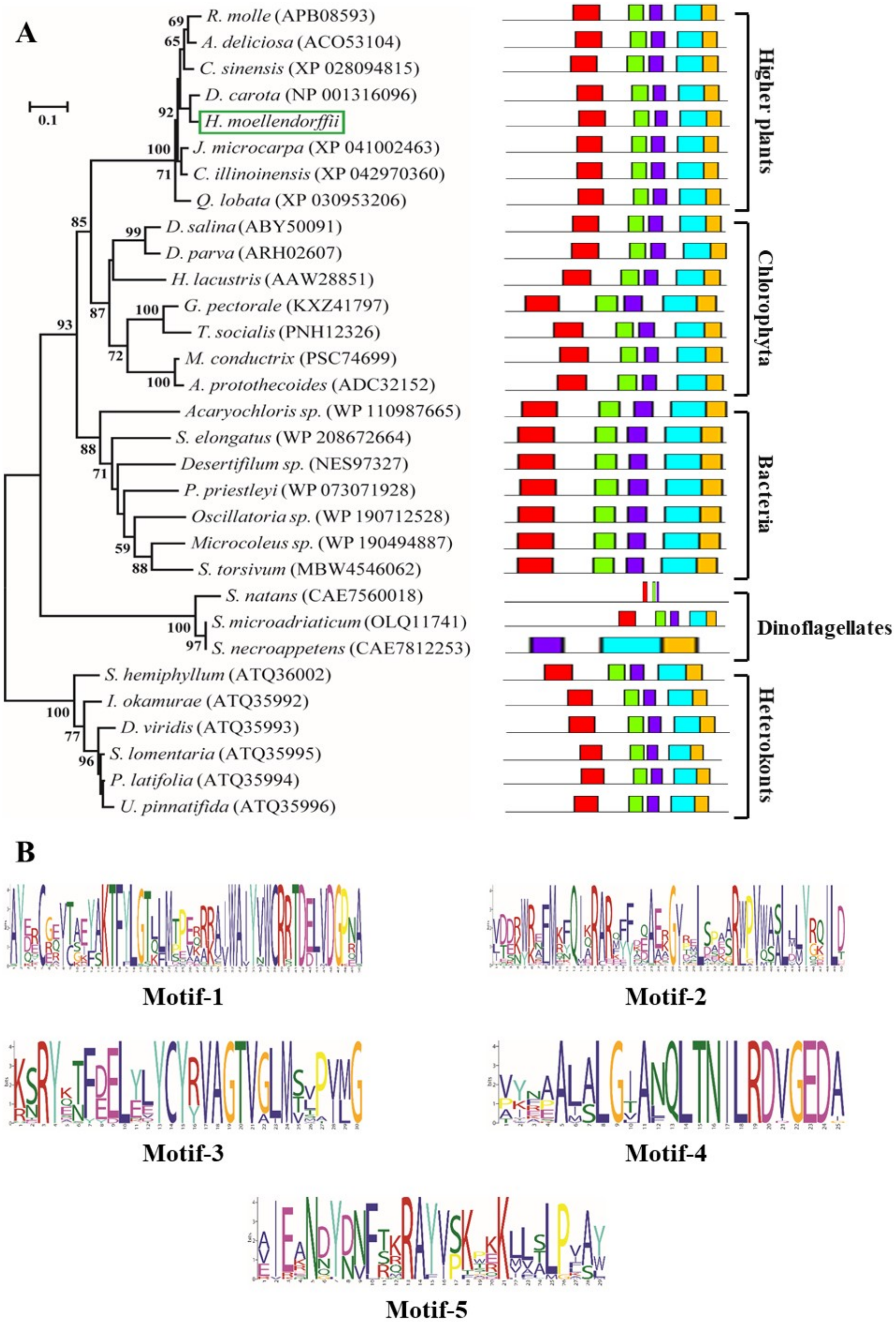

Figure S2B: Phylogeny of deduced HmPSY amino acid sequences along with other PSY sequences. The tree was constructed using MEGA 7 Kumar et al. [100] on the basis of the Neighbor-Joining method Saitou and Nei [101]. Bootstrap values on the nodes indicate the number of times each group occurred within 1000 replicates Felsenstein [102]. On the right side, the analysis of conserved protein motifs using MEME software is presented, showing motifs that are ordered in colored boxes corresponding to PSY sequence in the phylogenetic tree. Other protein sequences obtained from NCBI database as well as their accession numbers are provided in the bracket. (B) Conserved protein motif 1-5 present in the variable region of HmPSY gene. The height of the letters denotes the degree of conservation.

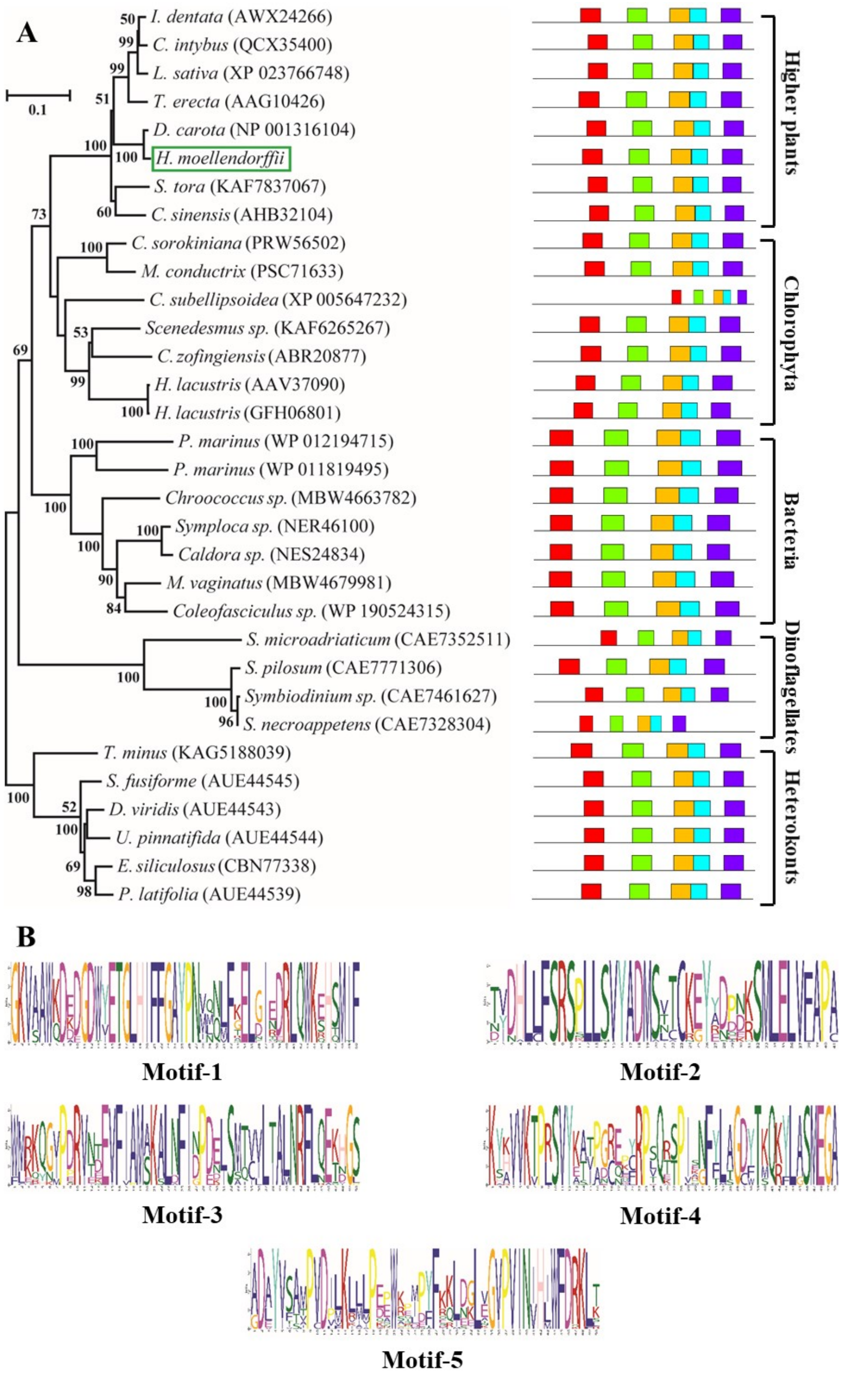

Figure S2C: Phylogeny of deduced HmPDS amino acid sequences along with other PDS sequences. The tree was constructed using MEGA 7 Kumar et al. [100] on the basis of the Neighbor–Joining method Saitou and Nei [101]. Bootstrap values on the nodes indicate the number of times each group occurred within 1000 replicates Felsenstein [102]. On the right side, the analysis of conserved protein motifs using MEME software is presented, showing motifs that are ordered in colored boxes corresponding to PDS sequence in the phylogenetic tree. Other protein sequences obtained from NCBI database as well as their accession numbers are provided in the bracket. (B) Conserved protein motif 1-5 present in the variable region of HmPDS gene. The height of the letters denotes the degree of conservation.

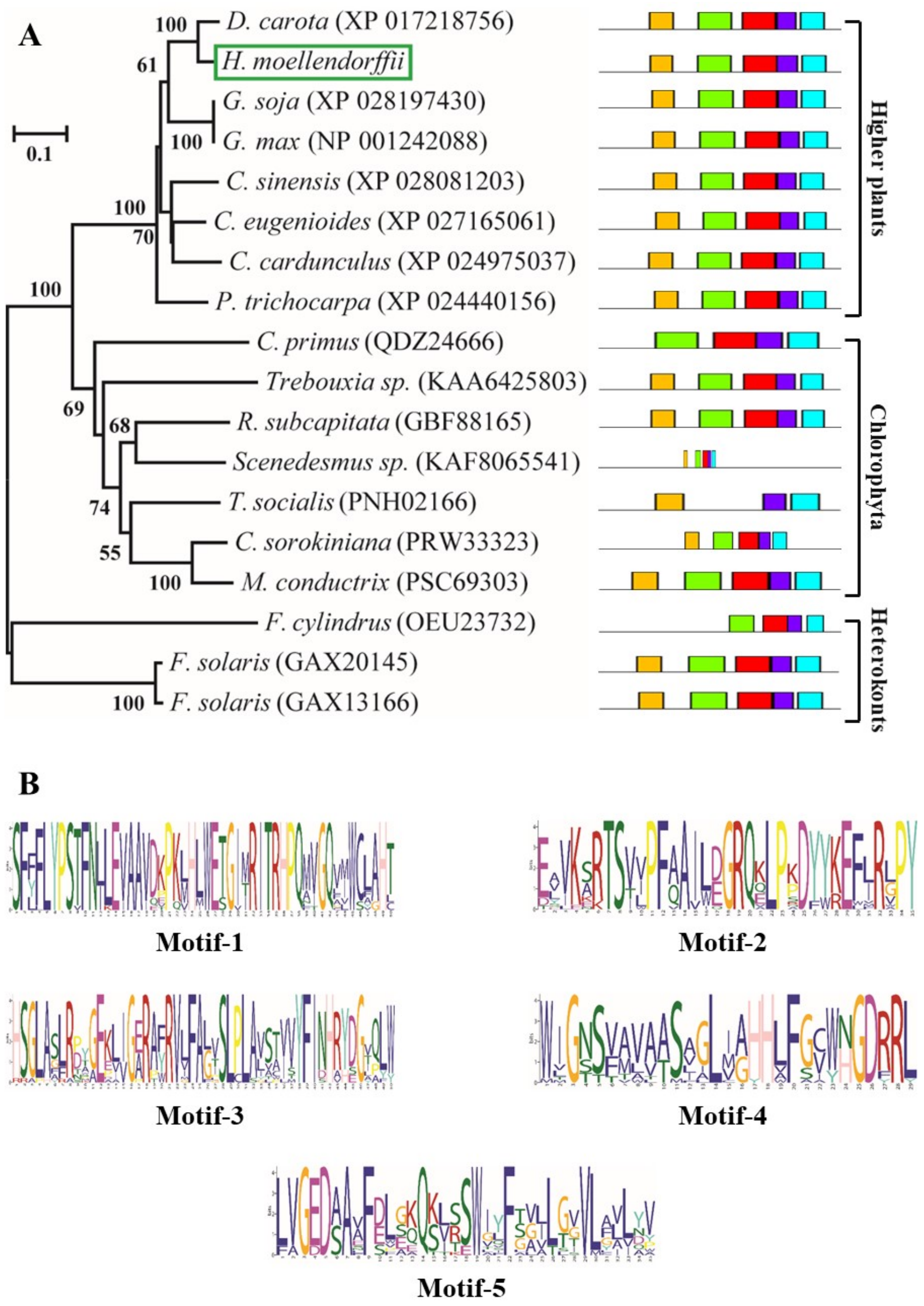

Figure S2D: Phylogeny of deduced HmZ-ISO amino acid sequences along with other Z-ISO sequences. The tree was constructed using MEGA 7 Kumar et al. [100] on the basis of the Neighbor–Joining method Saitou and Nei [101]. Bootstrap values on the nodes indicate the number of times each group occurred within 1000 replicates Felsenstein [102]. On the right side, the analysis of conserved protein motifs using MEME software is presented, showing motifs that are ordered in colored boxes corresponding to Z-ISO sequence in the phylogenetic tree. Other protein sequences obtained from NCBI database as well as their accession numbers are provided in the bracket. (B) Conserved protein motif 1-5 present in the variable region of HmZ-ISO gene. The height of the letters denotes the degree of conservation.

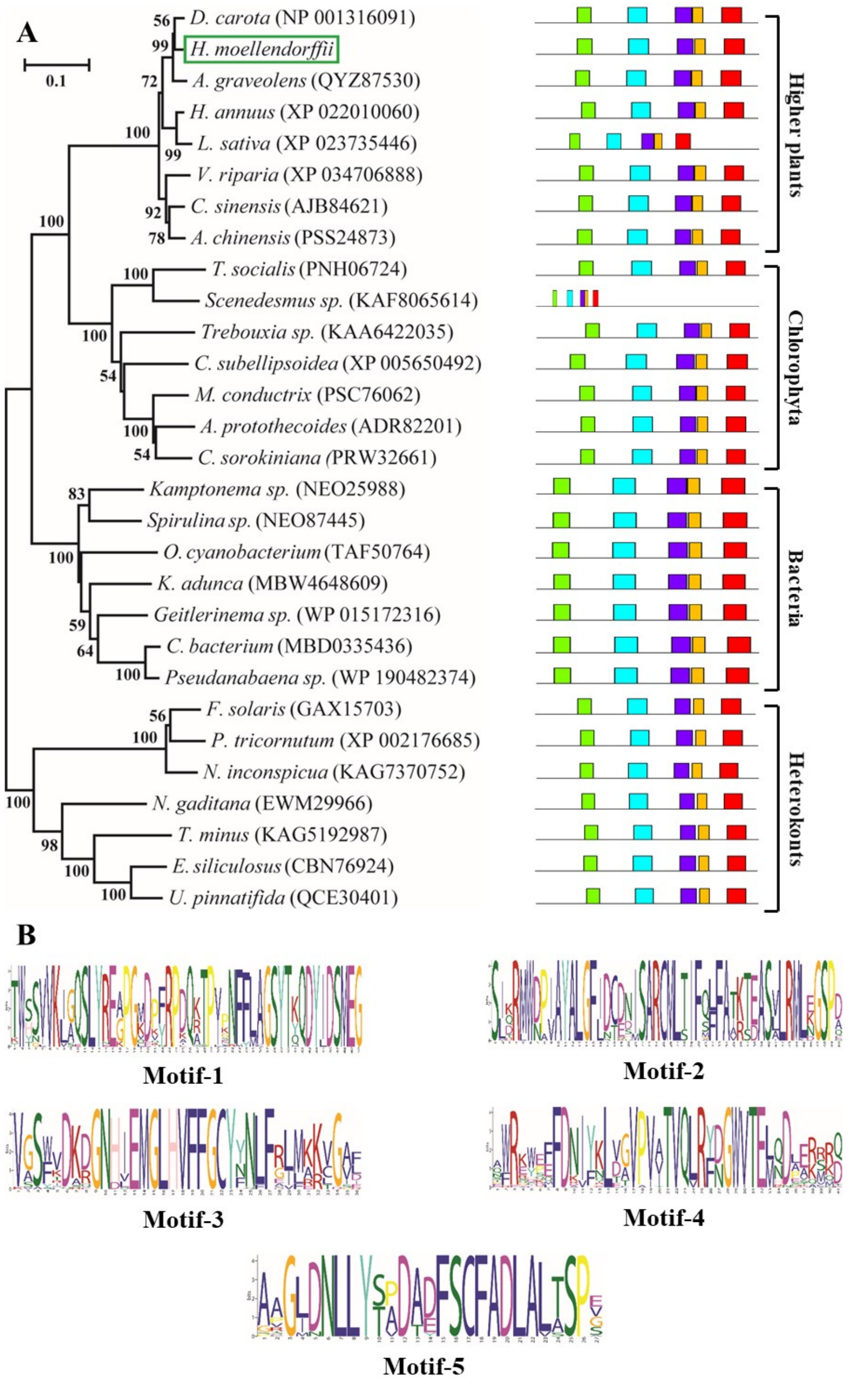

Figure S2E: Phylogeny of deduced HmZDS amino acid sequences along with other ZDS sequences. The tree was constructed using MEGA 7 Kumar et al. [100] on the basis of the Neighbor–Joining method Saitou and Nei [101]. Bootstrap values on the nodes indicate the number of times each group occurred within 1000 replicates Felsenstein [102]. On the right side, the analysis of conserved protein motifs using MEME software is presented, showing motifs that are ordered in colored boxes corresponding to ZDS sequence in the phylogenetic tree. Other protein sequences obtained from NCBI database as well as their accession numbers are provided in the bracket. (B) Conserved protein motif 1-5 present in the variable region of HmZDS gene. The height of the letters denotes the degree of conservation.

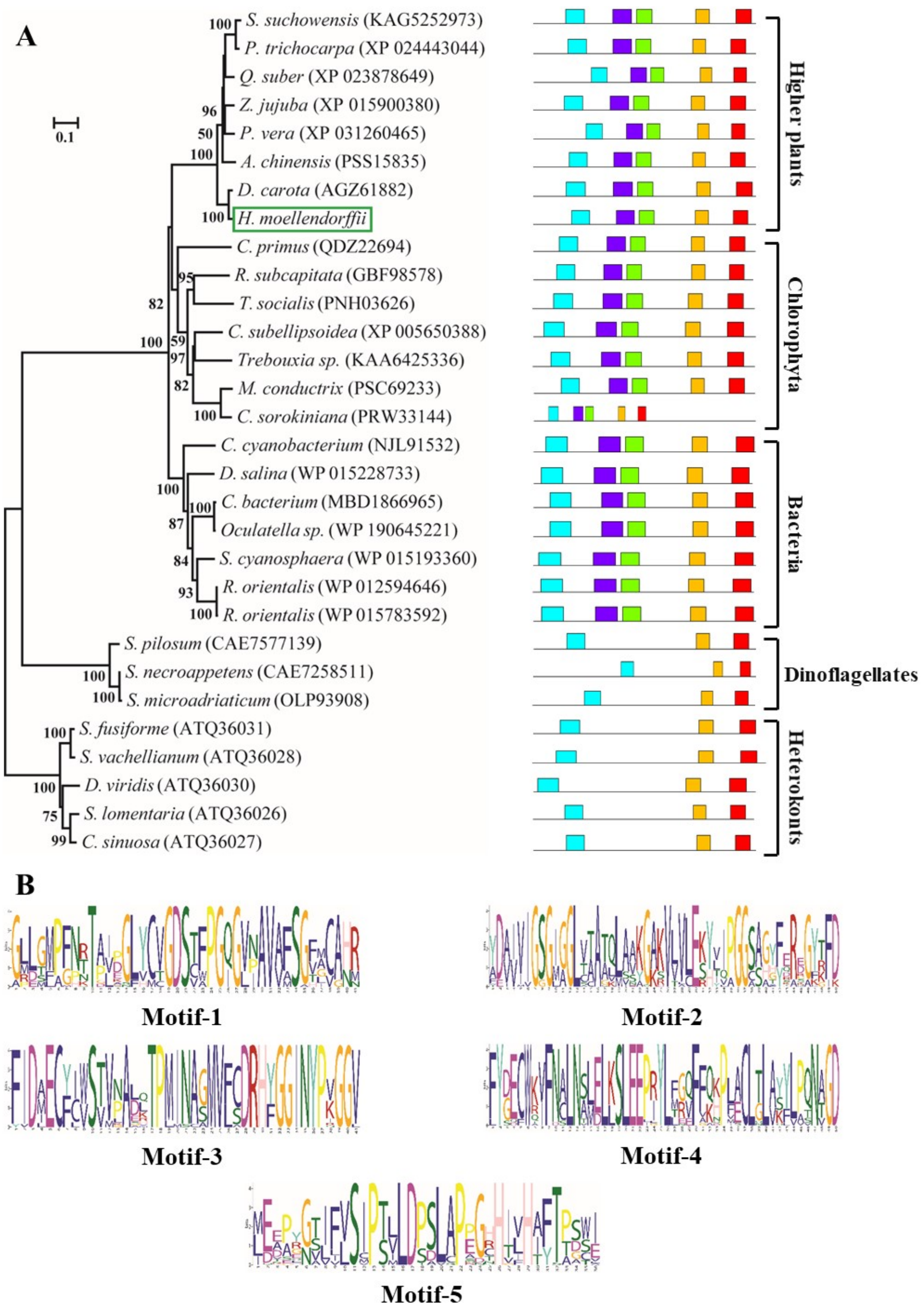

Figure S2F: Phylogeny of deduced HmCrtISO amino acid sequences along with other CrtISO sequences The tree was constructed using MEGA 7 Kumar et al. [100] on the basis of the Neighbor–Joining method Saitou and Nei [101]. Bootstrap values on the nodes indicate the number of times each group occurred within 1000 replicates Felsenstein [102]. On the right side, the analysis of conserved protein motifs using MEME software is presented, showing motifs that are ordered in colored boxes corresponding to CrtISO sequence in the phylogenetic tree. Other protein sequences obtained from NCBI database as well as their accession numbers are provided in the bracket. (B) Conserved protein motif 1-5 present in the variable region of HmCrtISO gene. The height of the letters denotes the degree of conservation.

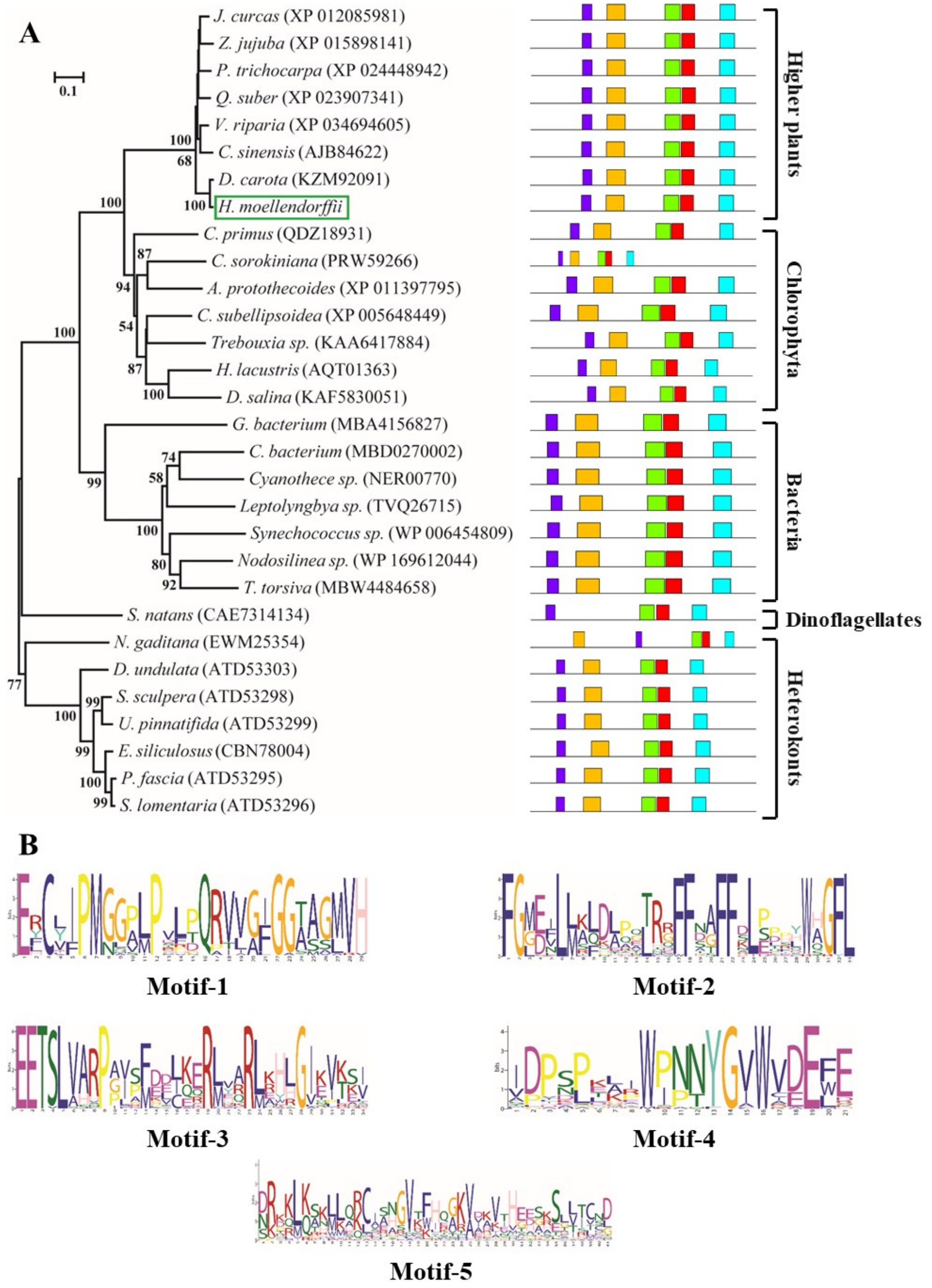

Figure S2G: Phylogeny of deduced HmLCYB amino acid sequences along with other LCYB sequences. The tree was constructed using MEGA 7 Kumar et al. [100] on the basis of the Neighbor–Joining method Saitou and Nei [101]. Bootstrap values on the nodes indicate the number of times each group occurred within 1000 replicates Felsenstein [102]. On the right side, the analysis of conserved protein motifs using MEME software is presented, showing motifs that are ordered in colored boxes corresponding to LCYB sequence in the phylogenetic tree. Other protein sequences obtained from NCBI database as well as their accession numbers are provided in the bracket. (B) Conserved protein motif 1-5 present in the variable region of HmLCYB gene. The height of the letters denotes the degree of conservation.

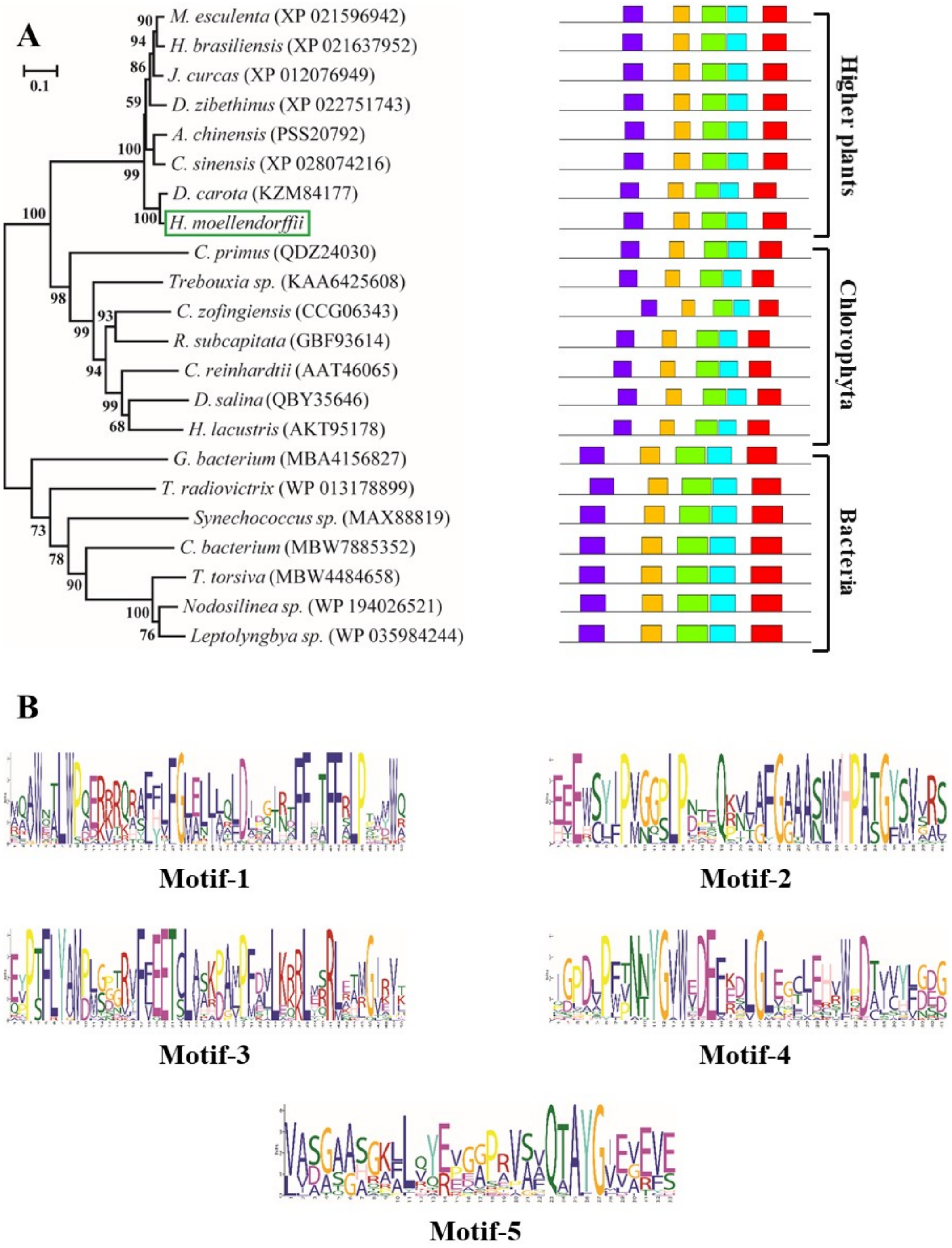

Figure S2H: Phylogeny of deduced HmLCYE amino acid sequences along with other LCYE sequences. The tree was constructed using MEGA 7 Kumar et al. [100] on the basis of the Neighbor–Joining method Saitou and Nei [101]. Bootstrap values on the nodes indicate the number of times each group occurred within 1000 replicates Felsenstein [102]. On the right side, the analysis of conserved protein motifs using MEME software is presented, showing motifs that are ordered in colored boxes corresponding to LCYE sequence in the phylogenetic tree. Other protein sequences obtained from NCBI database as well as their accession numbers are provided in the bracket. (B) Conserved protein motif 1-5 present in the variable region of HmLCYE gene. The height of the letters denotes the degree of conservation.

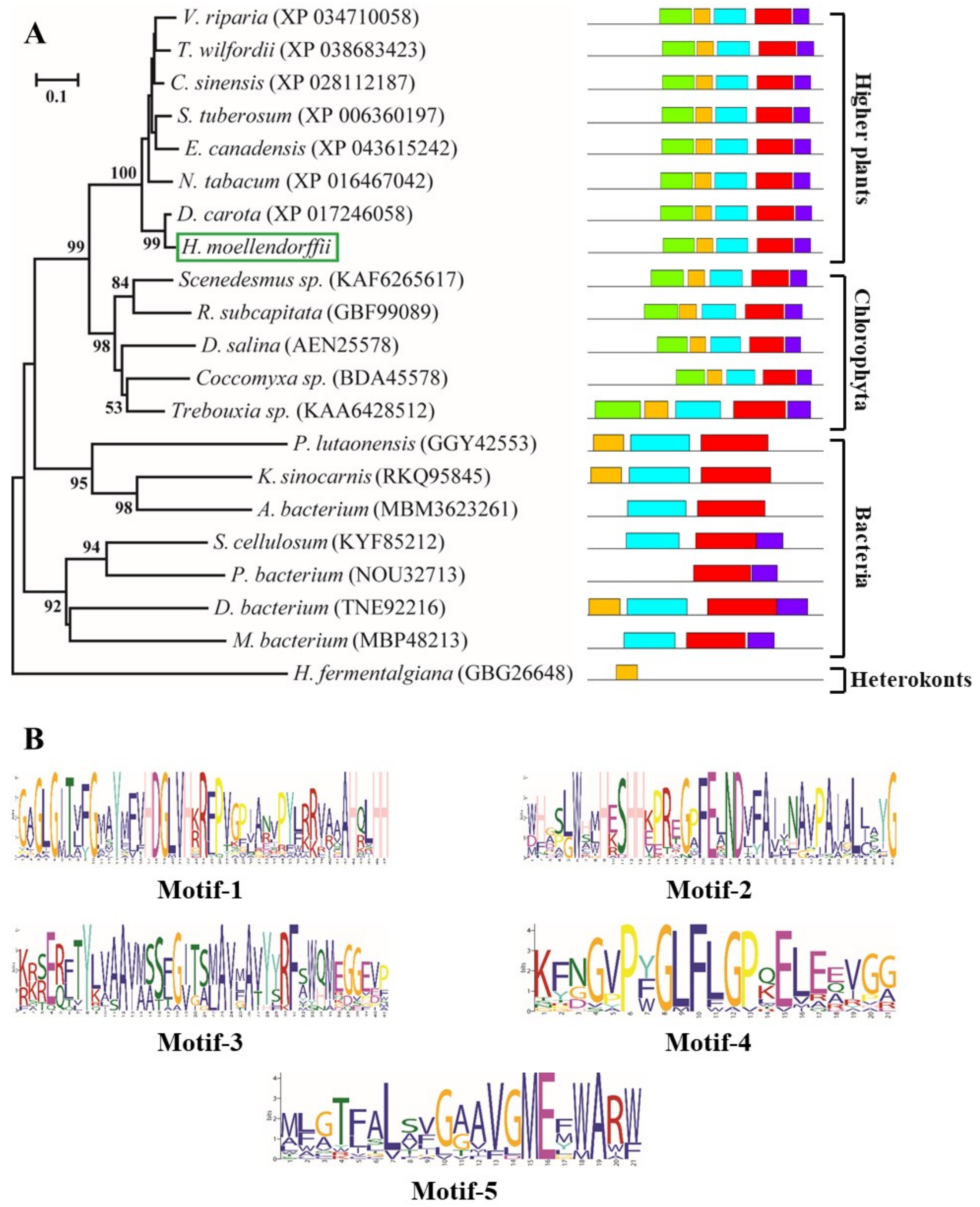

Figure S2I: Phylogeny of deduced HmCHXB amino acid sequences along with other CHXB sequences. The tree was constructed using MEGA 7 Kumar et al. [100] on the basis of the Neighbor–Joining method Saitou and Nei [101]. Bootstrap values on the nodes indicate the number of times each group occurred within 1000 replicates Felsenstein [102]. On the right side, the analysis of conserved protein motifs using MEME software is presented, showing motifs that are ordered in colored boxes corresponding to CHXB sequence in the phylogenetic tree. Other protein sequences obtained from NCBI database as well as their accession numbers are provided in the bracket. (B) Conserved protein motif 1-5 present in the variable region of HmCHXB gene. The height of the letters denotes the degree of conservation.

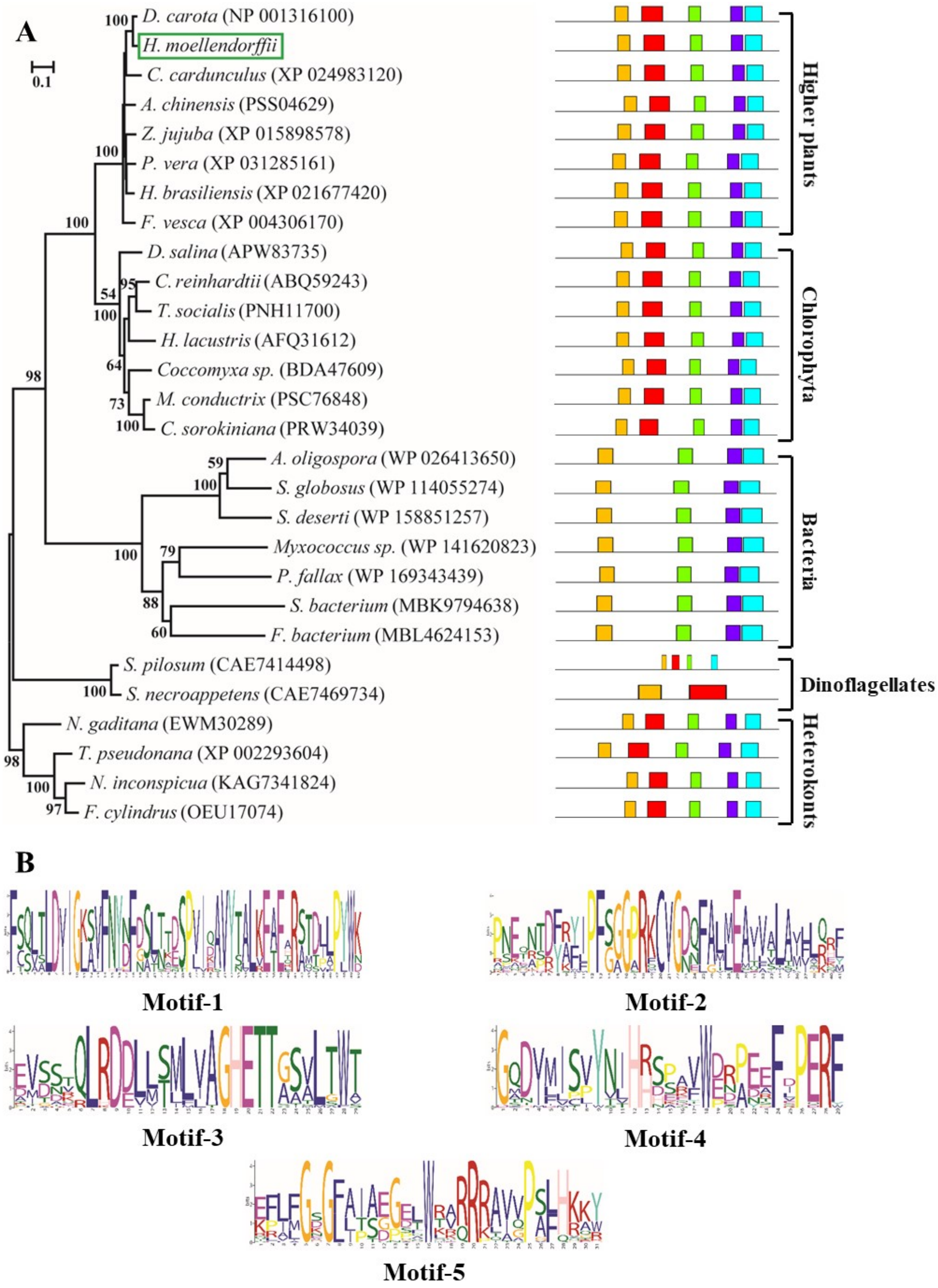

Figure S2J: Phylogeny of deduced HmCHXE amino acid sequences along with other CHXE sequences. The tree was constructed using MEGA 7 Kumar et al. [100] on the basis of the Neighbor–Joining method Saitou and Nei [101]. Bootstrap values on the nodes indicate the number of times each group occurred within 1000 replicates Felsenstein [102]. On the right side, the analysis of conserved protein motifs using MEME software is presented, showing motifs that are ordered in colored boxes corresponding to CHXE sequence in the phylogenetic tree. Other protein sequences obtained from NCBI database as well as their accession numbers are provided in the bracket. (B) Conserved protein motif 1-5 present in the variable region of HmCHXE gene. The height of the letters denotes the degree of conservation.

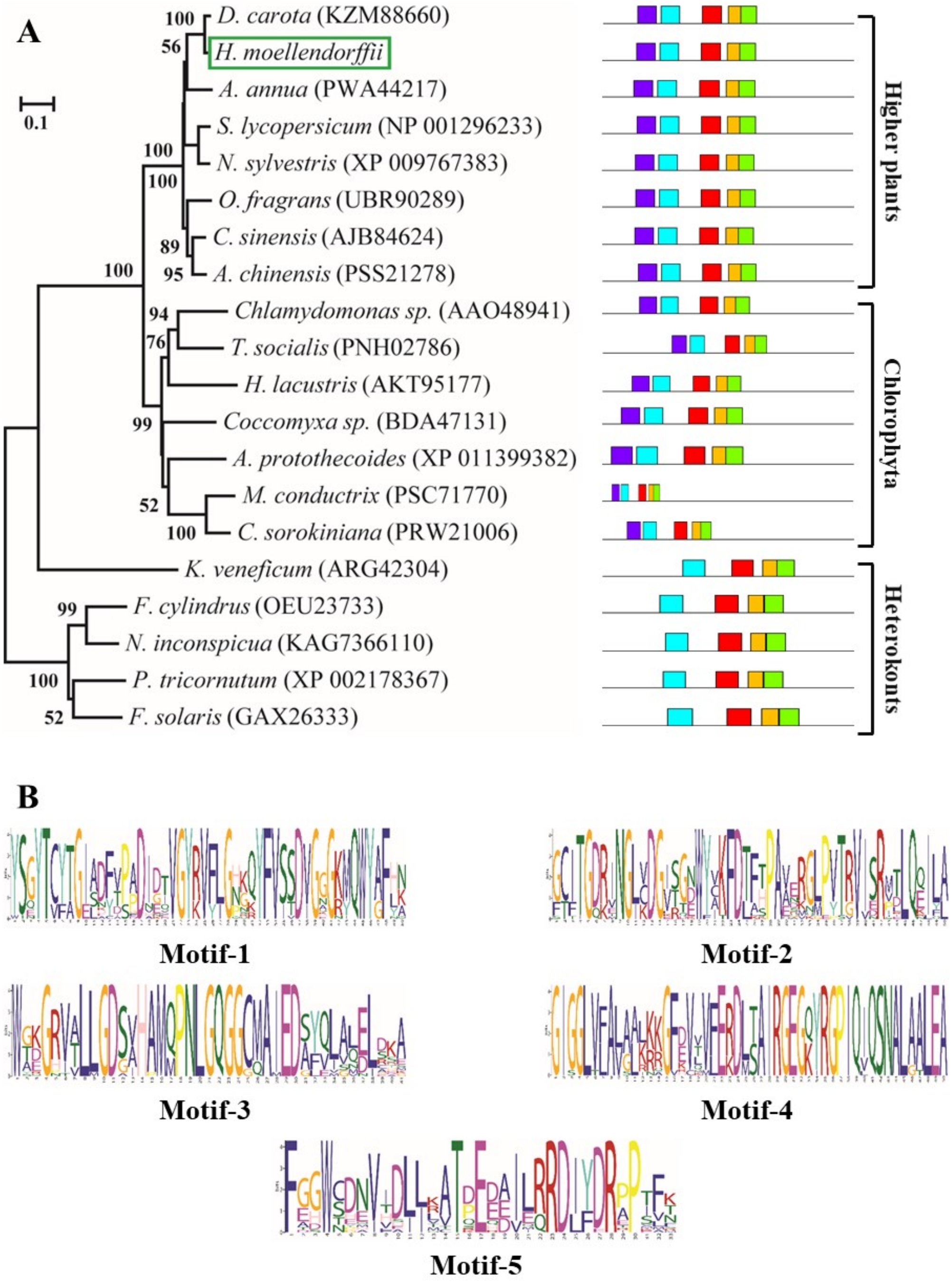

Figure S2K: Phylogeny of deduced HmZEP amino acid sequences along with other ZEP sequences. The tree was constructed using MEGA 7 Kumar et al. [100] on the basis of the Neighbor–Joining method Saitou and Nei [101]. Bootstrap values on the nodes indicate the number of times each group occurred within 1000 replicates Felsenstein [102]. On the right side, the analysis of conserved protein motifs using MEME software is presented, showing motifs that are ordered in colored boxes corresponding to ZEP sequence in the phylogenetic tree. Other protein sequences obtained from NCBI database as well as their accession numbers are provided in the bracket. (B) Conserved protein motif 1-5 present in the variable region of HmZEP gene. The height of the letters denotes the degree of conservation.

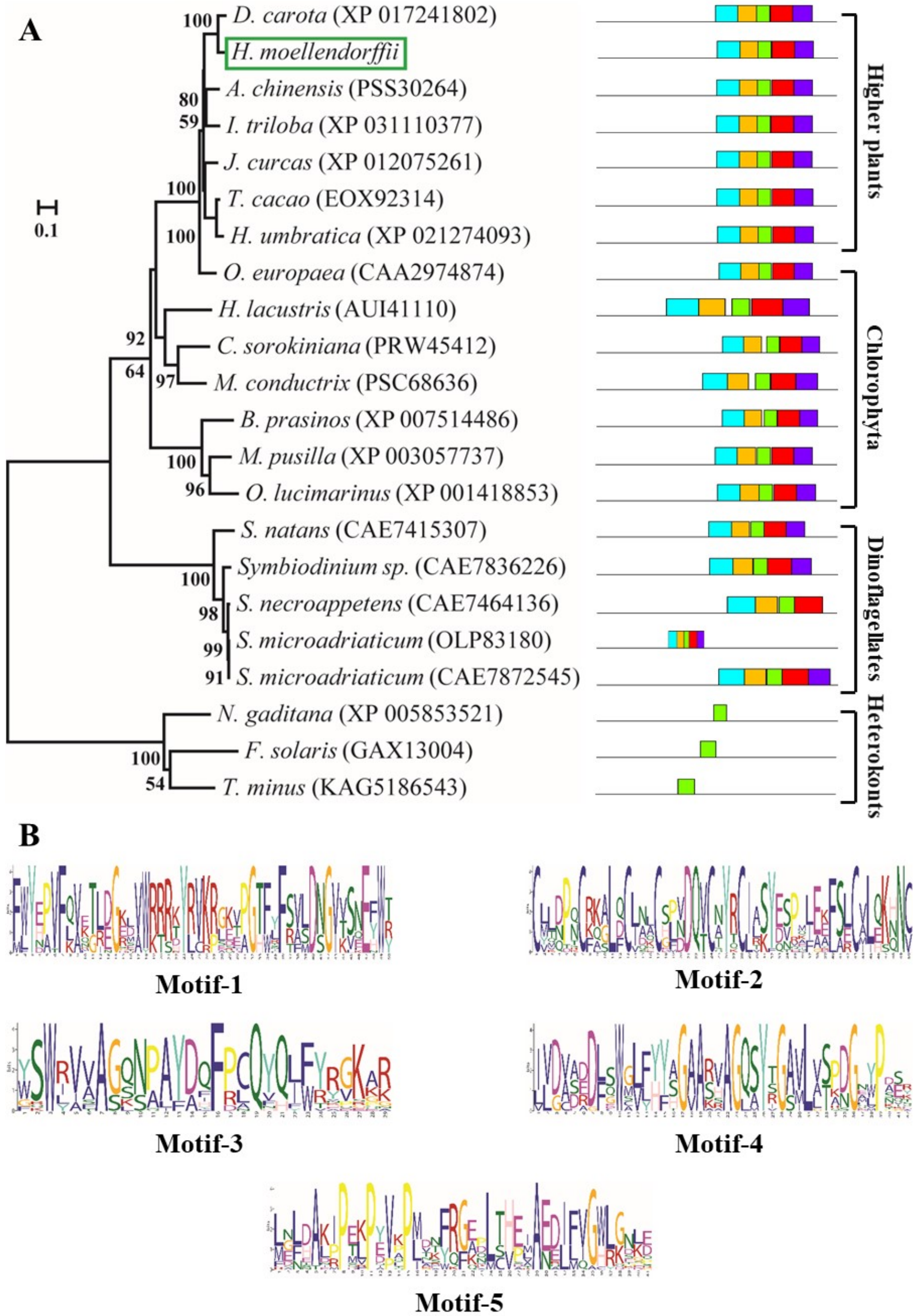

Figure S2L: Phylogeny of deduced HmVDE amino acid sequences along with other VDE sequences. The tree was constructed using MEGA 7 Kumar et al. [100] on the basis of the Neighbor–Joining method Saitou and Nei [101]. Bootstrap values on the nodes indicate the number of times each group occurred within 1000 replicates Felsenstein [102]. On the right side, the analysis of conserved protein motifs using MEME software is presented, showing motifs that are ordered in colored boxes corresponding to VDE sequence in the phylogenetic tree. Other protein sequences obtained from NCBI database as well as their accession numbers are provided in the bracket. (B) Conserved protein motif 1-5 present in the variable region of HmVDE gene. The height of the letters denotes the degree of conservation.

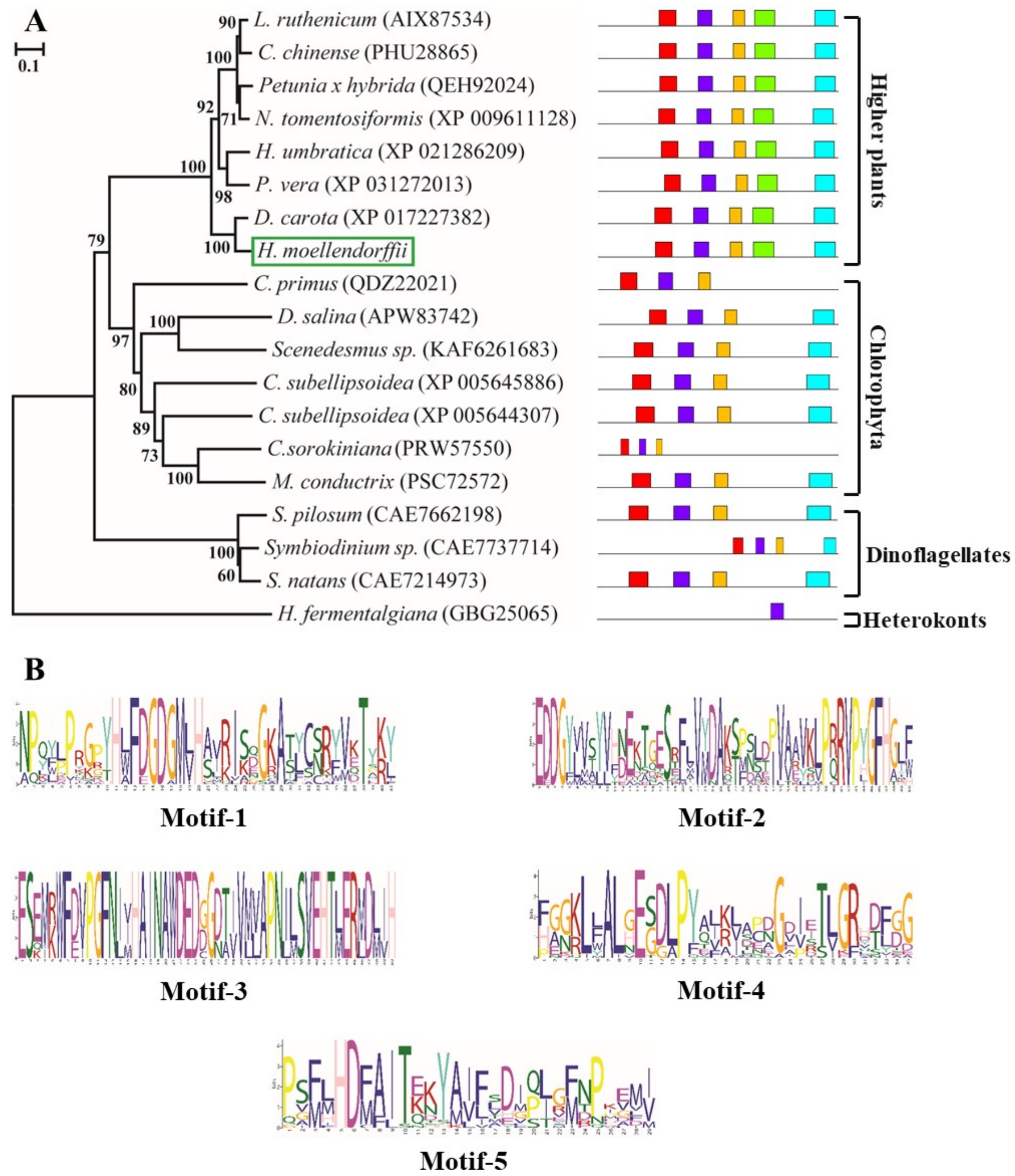

Figure S2M: Phylogeny of deduced HmCCD amino acid sequences along with other CCD sequences. The tree was constructed using MEGA 7 Kumar et al. [100] on the basis of the Neighbor–Joining method Saitou and Nei [101]. Bootstrap values on the nodes indicate the number of times each group occurred within 1000 replicates Felsenstein [102]. On the right side, the analysis of conserved protein motifs using MEME software is presented, showing motifs that are ordered in colored boxes corresponding to CCD sequence in the phylogenetic tree. Other protein sequences obtained from NCBI database as well as their accession numbers are provided in the bracket. (B) Conserved protein motif 1-5 present in the variable region of HmCCD gene. The height of the letters denotes the degree of conservation.

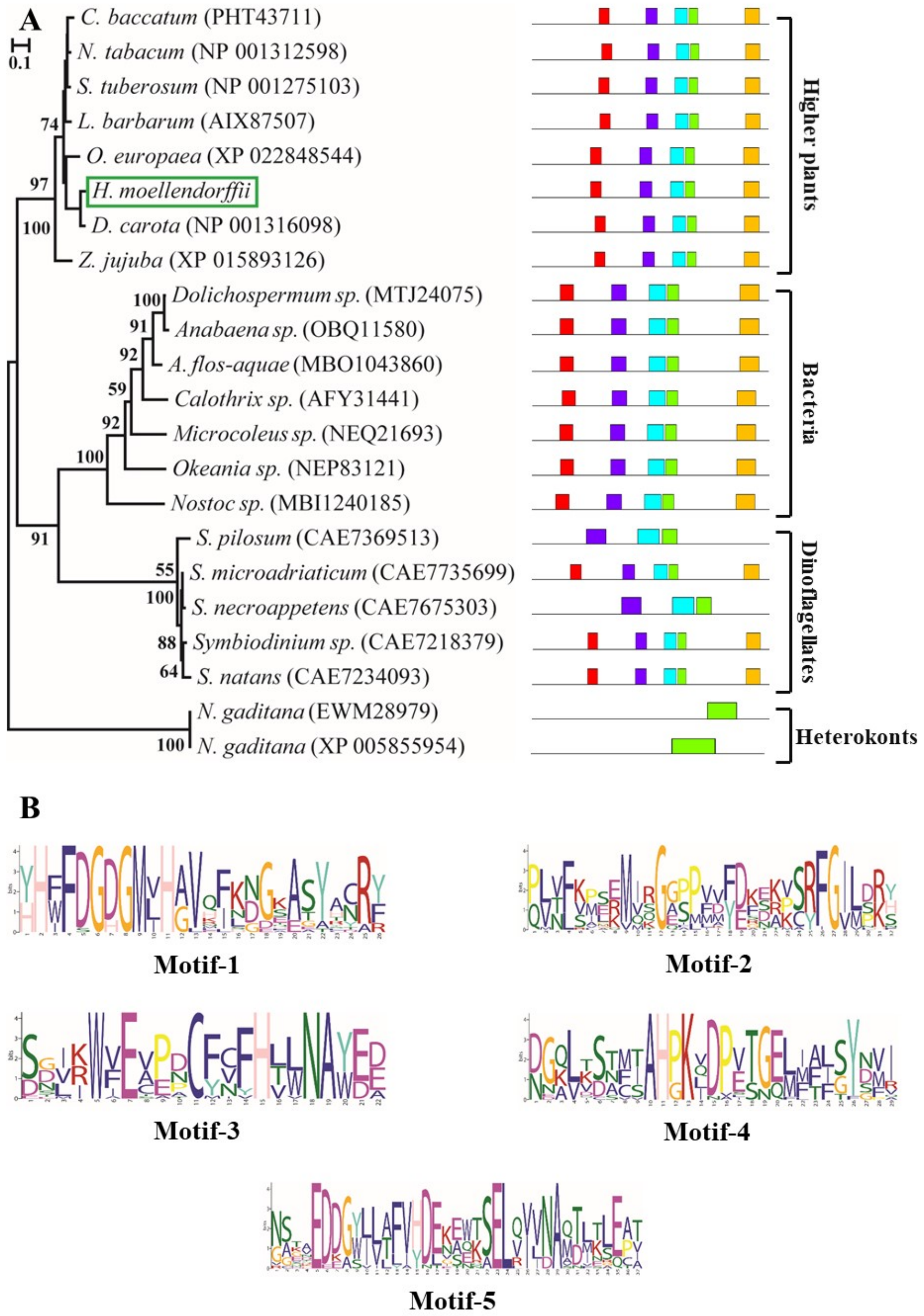

Figure S2N: Phylogeny of deduced HmNCED amino acid sequences along with other NCED sequences. The tree was constructed using MEGA 7 Kumar et al. [100] on the basis of the Neighbor–Joining method Saitou and Nei [101]. Bootstrap values on the nodes indicate the number of times each group occurred within 1000 replicates Felsenstein [102]. On the right side, the analysis of conserved protein motifs using MEME software is presented, showing motifs that are ordered in colored boxes corresponding to NCED sequence in the phylogenetic tree. Other protein sequences obtained from NCBI database as well as their accession numbers are provided in the bracket. (B) Conserved protein motif 1-5 present in the variable region of HmNCED gene. The height of the letters denotes the degree of conservation.

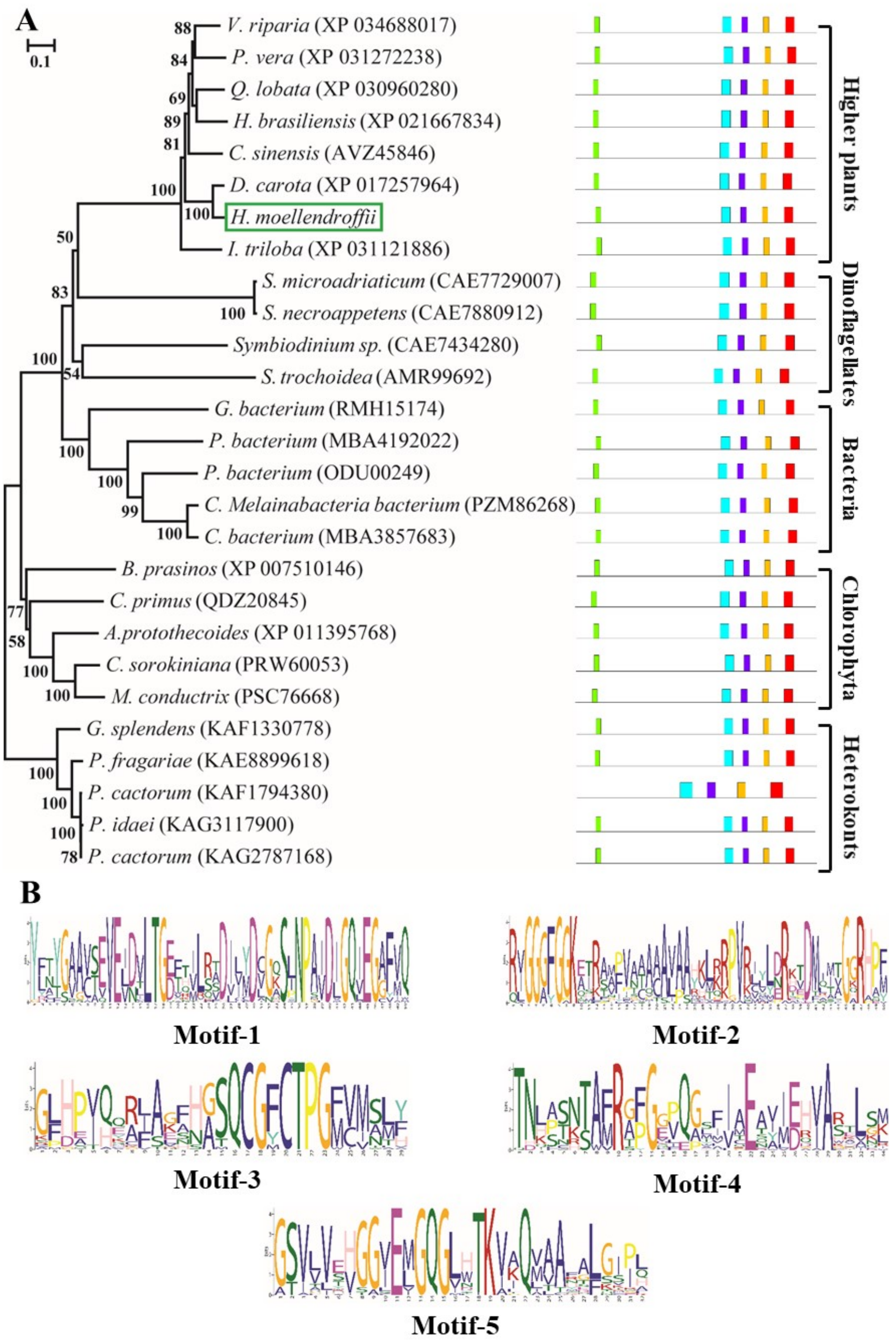

Figure S2O: Phylogeny of deduced HmAO amino acid sequences along with other AO sequences. The tree was constructed using MEGA 7 Kumar et al. [100] on the basis of the Neighbor–Joining method Saitou and Nei [101]. Bootstrap values on the nodes indicate the number of times each group occurred within 1000 replicates Felsenstein [102]. On the right side, the analysis of conserved protein motifs using MEME software is presented, showing motifs that are ordered in colored boxes corresponding to AO sequence in the phylogenetic tree. Other protein sequences obtained from NCBI database as well as their accession numbers are provided in the bracket. (B) Conserved protein motif 1-5 present in the variable region of HmAO gene. The height of the letters denotes the degree of conservation.

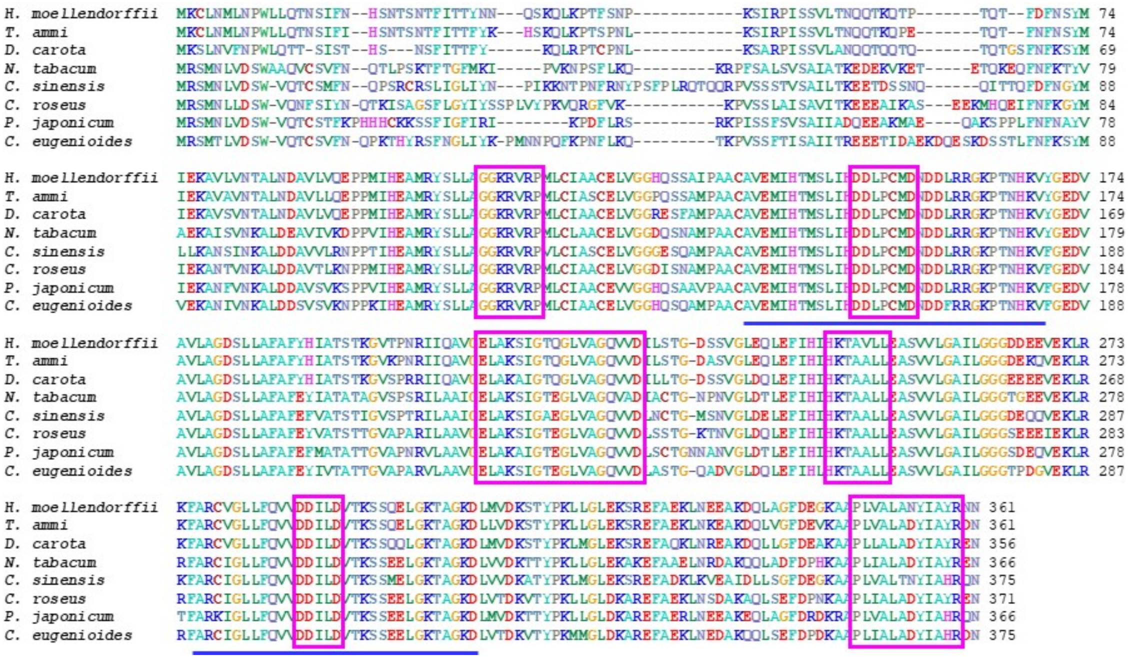

Figure S3A. Amino acid alignment of HmGGPS with selected corresponding genes was performed with the BioEdit program of ClustalW. The pink box represents the five conserved domains. Blue underline represents the aspartate-rich motifs. *T. ammi* (*Trachyspermum ammi*, AUZ98417), *D. carota* (*Daucus carota,* XP_017246904), *N. tabacum* (*Nicotiana tabacum*, XP_016435651), *C. sinensis* (*Camellia sinensis*, XP_028108146), *C. roseus* (*Catharanthus roseus*, AGL91648), *P. japonicum* (*Phtheirospermum japonicum*, GFP99862), and *C. eugenioides* (*Coffea eugenioides*, XP_027148302), were involved in amino acid alignment of HmGGPS. A dash (–) represents an aligned gap.

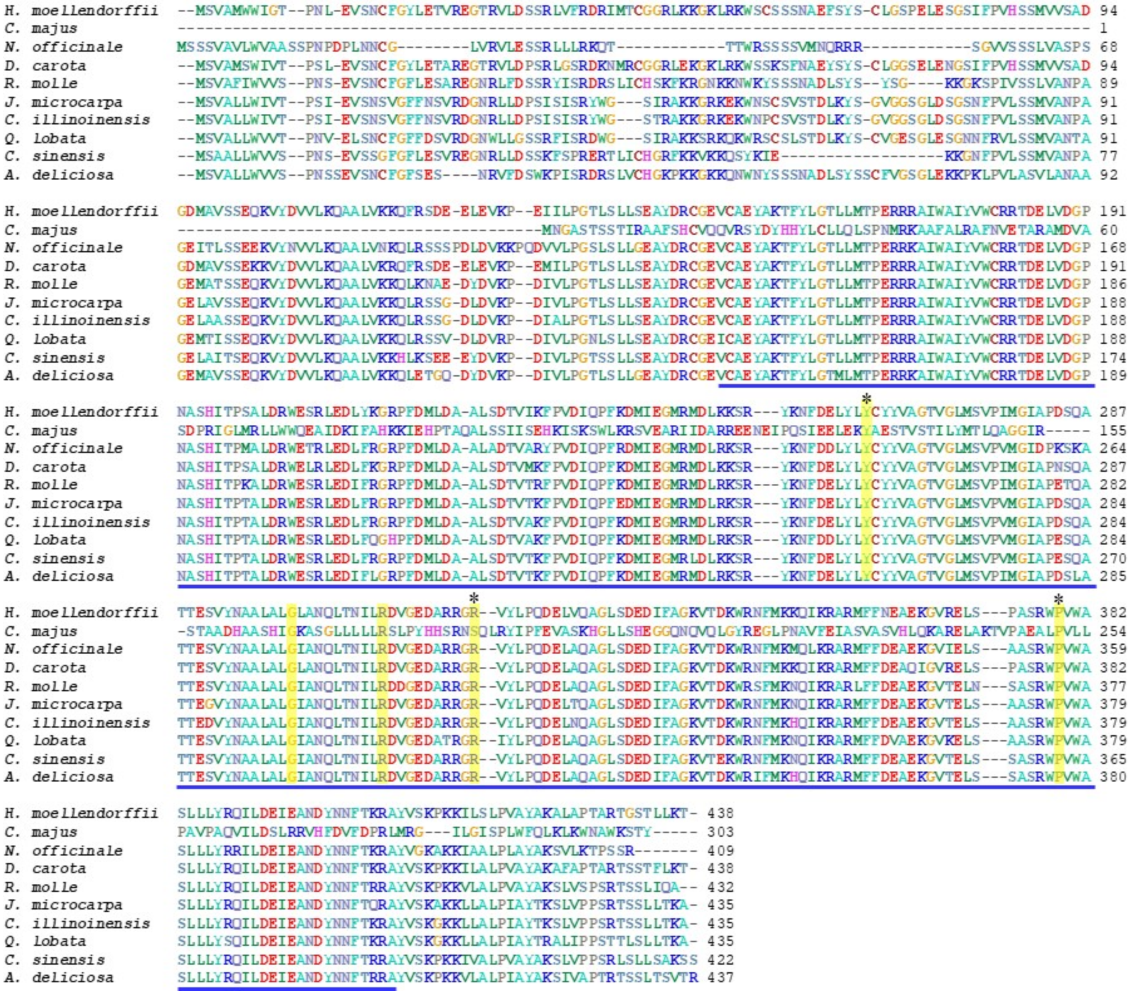

Figure S3B. Amino acid alignment of HmPSY with selected corresponding genes was performed with the BioEdit program of ClustalW. The yellow highlighted box represents the substrate-binding pocket. Asterisks indicate catalytic residues, and those with a blue underline represent the conserved trans-isoprenyl diphosphate synthase domain. *C. majus* (*Chelidonium majus*, MW307341), *N. officinale* (*Nasturtium officinale*, MT547989), *D. carota* (*Daucus carota*, NP_001316096), *R. molle* (*Rhododendron molle*, APB08593), *J. microcarpa* (*Juglans microcarpa*, XP_041002463), *C. illinoinensis* (*Carya illinoinensis*, XP_042970360), *Q. lobata* (*Quercus lobata*, XP_030953206), *C. sinensis* (*Camellia sinensis*, XP_028094815), and *A. deliciosa* (*Actinidia deliciosa*, XP_028094815), were involved in amino acid alignment of HmPSY. A dash (–) represents an aligned gap.

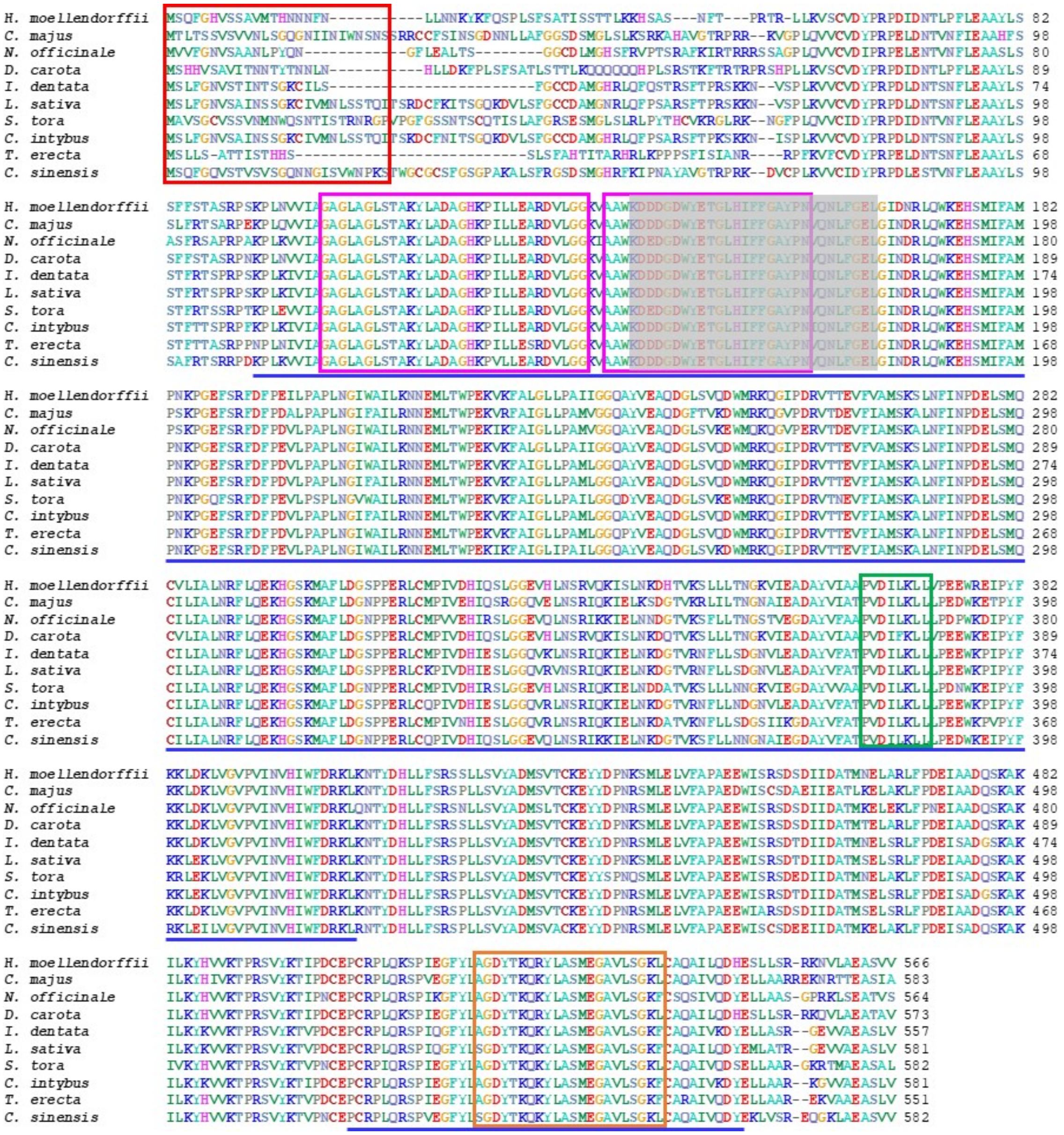

Figure S3C. Amino acid alignment of HmPDS with selected corresponding genes was performed with the BioEdit program of ClustalW. The red, pink, green, and orange boxes represent the signal peptide, dinucleotide-binding motif, putative substrate carrier motif, and carotenoid binding motif, respectively. A highly conserved region of the PDS protein was highlighted in an ash color. The blue underline represents the FAD/NAD(P)-binding domain. *C. majus* (*Chelidonium majus,* MW307330), *N. officinale* (*Nasturtium officinale*, MT547988), *D. carota* (*Daucus carota*, NP_001316104), *Ixeris dentata* (*I. dentata*, AWX24266), *L. sativa* (*Lactuca sativa*, XP_023766748), *S. tora* (*Senna tora*, KAF7837067), *C. intybus* (*Cichorium intybus*, QCX35400), *T. erecta* (*Tagetes erecta*, AAG10426), and *C. sinensis* (*Camellia sinensis*, AHB32104) were involved in amino acid alignment of HmPDS. A dash (–) represents an aligned gap.

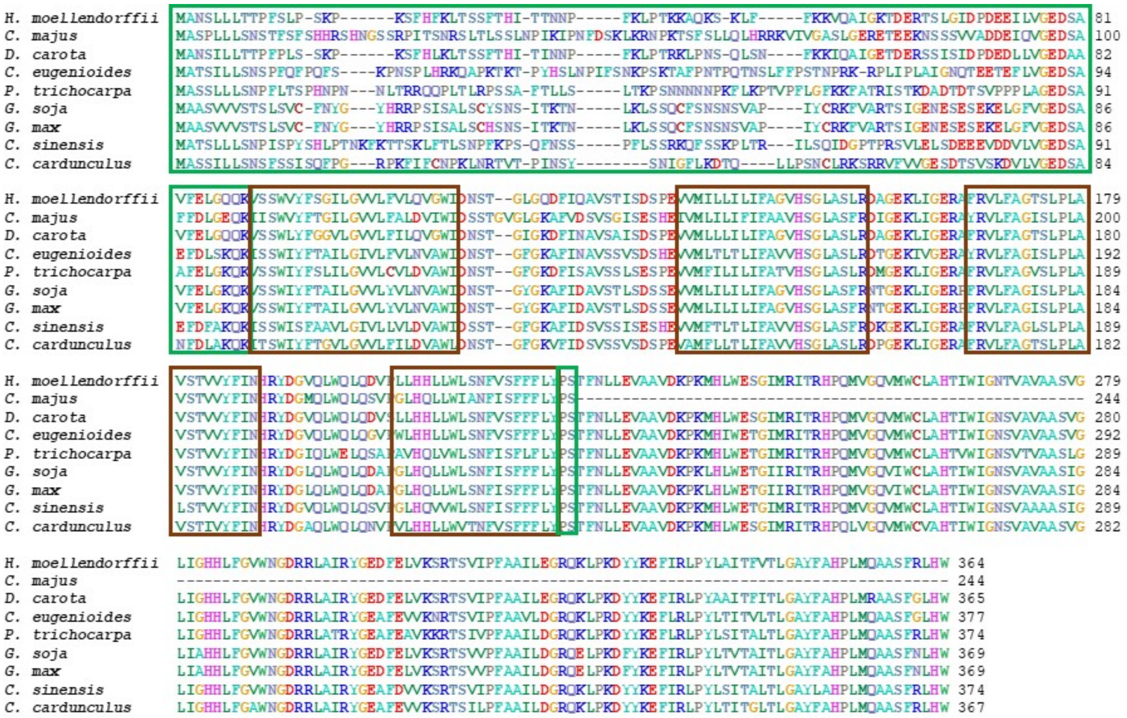

Figure S3D. Amino acid alignment of HmZ-ISO with selected corresponding genes was performed with the BioEdit program of ClustalW. Green and brown boxes represent the cytoplasmic domain and predicted trans membrane (TM) helix, respectively. *C. majus* (*Chelidonium majus,* MW307331), *D. carota* (*Daucus carota*, XP_017218756), *C. eugenioides* (*Coffea eugenioides*, XP_027165061), *P. trichocarpa* (*Populus trichocarpa*, XP_024440156), *G. soja* (*Glycine soja*, XP_028197430), *G. max* (*Glycine max*, NP_001242088), *C. sinensis* (*Camellia sinensis*, XP_028081203), and *C. cardunculus* (*Cynara cardunculus*, XP_024975037) were involved in amino acid alignment of HmZ-ISO. A dash (–) represents an aligned gap.

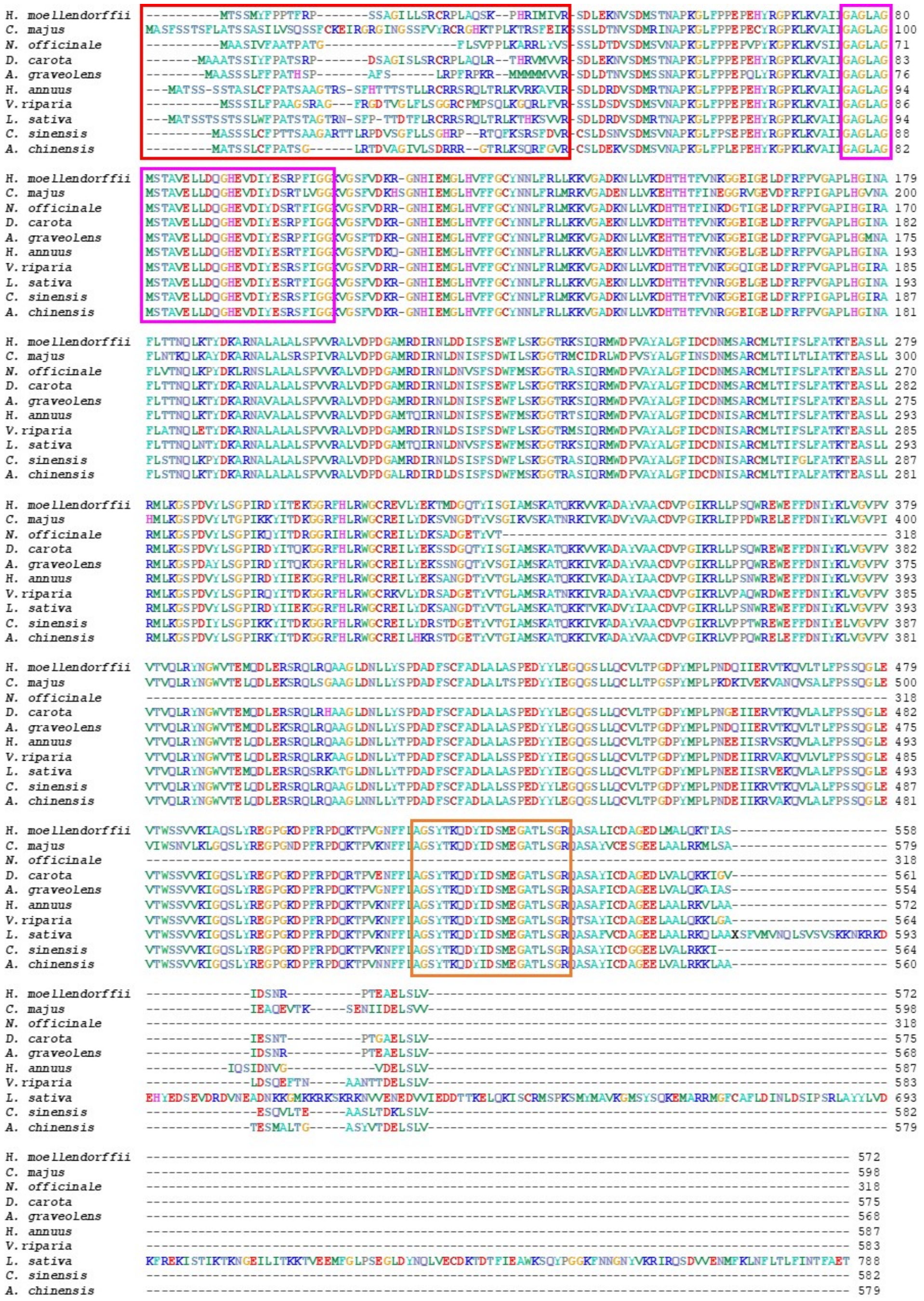

Figure S3E. Amino acid alignment of HmZDS with selected corresponding genes was performed with the BioEdit program of ClustalW. The red, pink, and orange boxes represent the signal peptide, dinucleotide-binding motif, and carotenoid binding motif, respectively. *C. majus* (*Chelidonium majus*, MW307344), *N. officinale* (*Nasturtium officinale*, MT547991), *D. carota* (*Daucus carota*, NP_001316091), *A. graveolens* (*Apium graveolens*, QYZ87530), *H. annuus* (*Helianthus annuus*, XP_022010060), *V. riparia* (*Vitis riparia*, XP_034706888), *L. sativa* (*Lactuca sativa*, XP_023735446), *C. sinensis* (*Camellia sinensis*, AJB84621), and *A. chinensis* (*Actinidia chinensis*, PSS24873) were involved in amino acid alignment of HmZDS. A dash (–) represents an aligned gap.

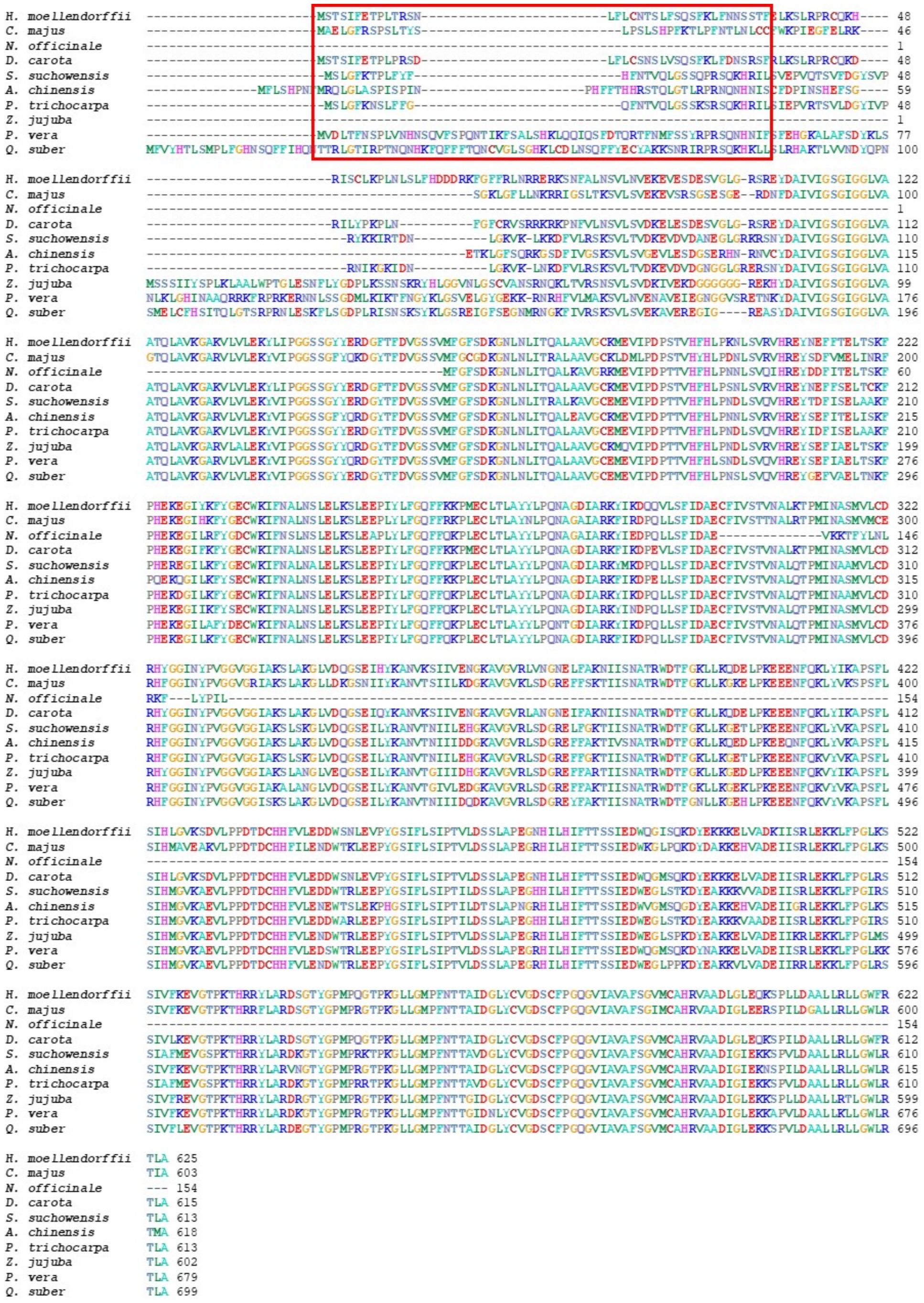

Figure S3F. Amino acid alignment of HmCrtISO with selected corresponding genes was performed with the BioEdit program of ClustalW. The red box represents the signal peptide. *C. majus* (*Chelidonium majus*, MW307336), *N. officinale* (*Nasturtium officinale*, MT547983), *D. carota* (*Daucus carota*, AGZ61882), *S. suchowensis* (*Salix suchowensis*, KAG5252973), *A. chinensis* (*Actinidia chinensis*, PSS15835), *P. trichocarpa* (*Populus trichocarpa*, XP_024443044), *Z. jujuba* (*Ziziphus jujuba*, XP_015900380), *P. vera* (*Pistacia vera*, XP_031260465), and *Q. suber* (*Quercus suber*, XP_023878649) were involved in amino acid alignment of HmCrtISO. A dash (–) represents an aligned gap.

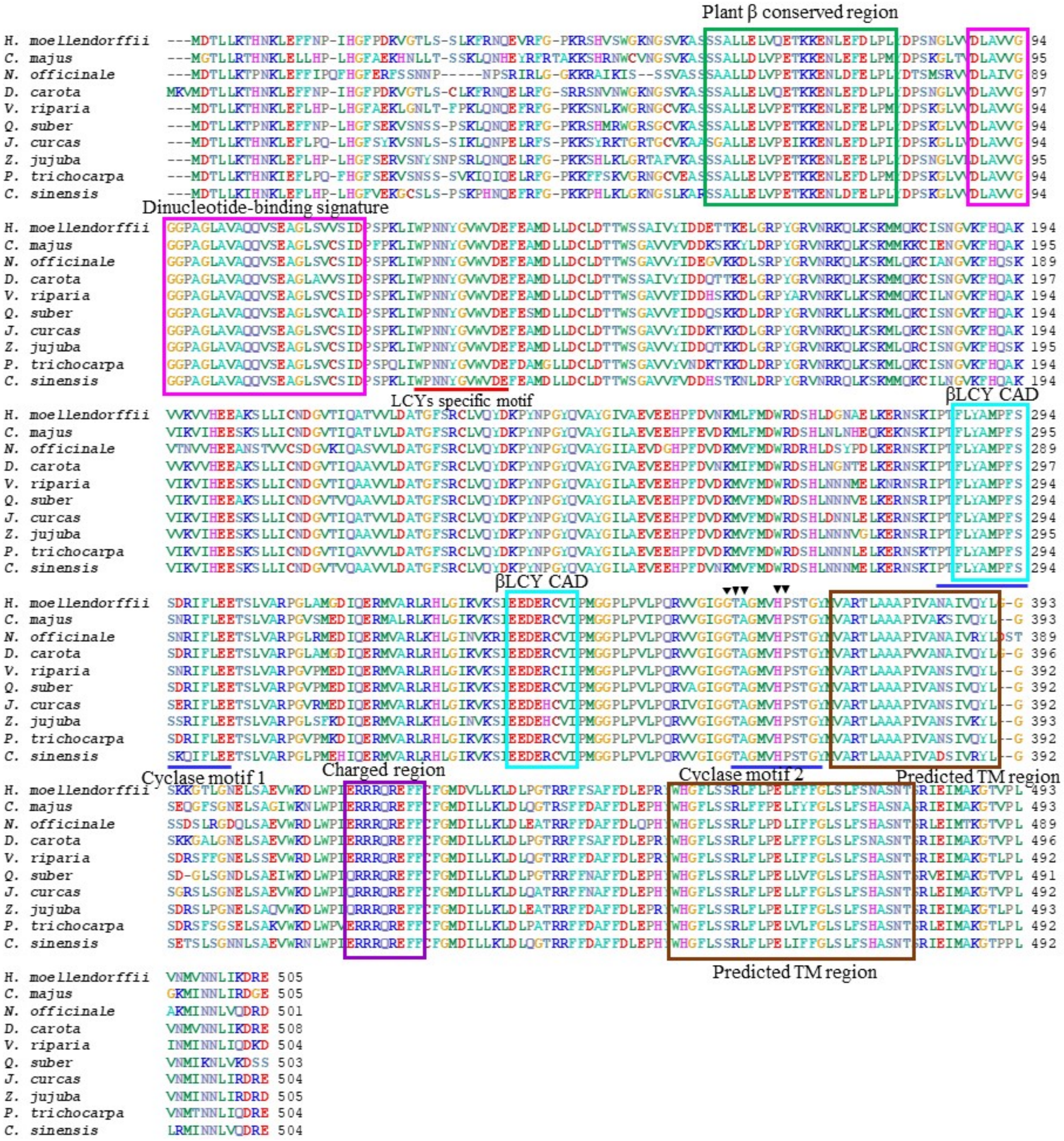

Figure S3G. Amino acid alignment of HmLCYB with selected corresponding genes was performed with the BioEdit program of ClustalW. The green, pink, light blue, brown, and purple boxes represent plant β conserved region, dinucleotide-binding motif, βLCY catalytic domain (CAD), predicted trans membrane (TM) helix, and charged region, respectively. The red underlined indicates LCY specific motif, whereas the blue underlined indicates cyclase motif 1 and 2. The highlighted arrowheads indicate the regulatory motif conserved in all β-bicyclase. *C. majus* (*Chelidonium majus*, MW307338), *N. officinale* (*Nasturtium officinale*, MT547985), *D. carota* (*Daucus carota*, KZM92091), *V. riparia* (*Vitis riparia*, XP_034694605), *Q. suber* (*Quercus suber*, XP_023907341), *J. curcas* (*Jatropha curcas*, XP_012085981), *Z. jujuba* (*Ziziphus jujuba*, XP_015898141), *P. trichocarpa* (*Populus trichocarpa*, XP_024448942), and *C. sinensis* (*Camellia sinensis*, AJB84622) were involved in amino acid alignment of HmLCYB. A dash (–) represents an aligned gap.

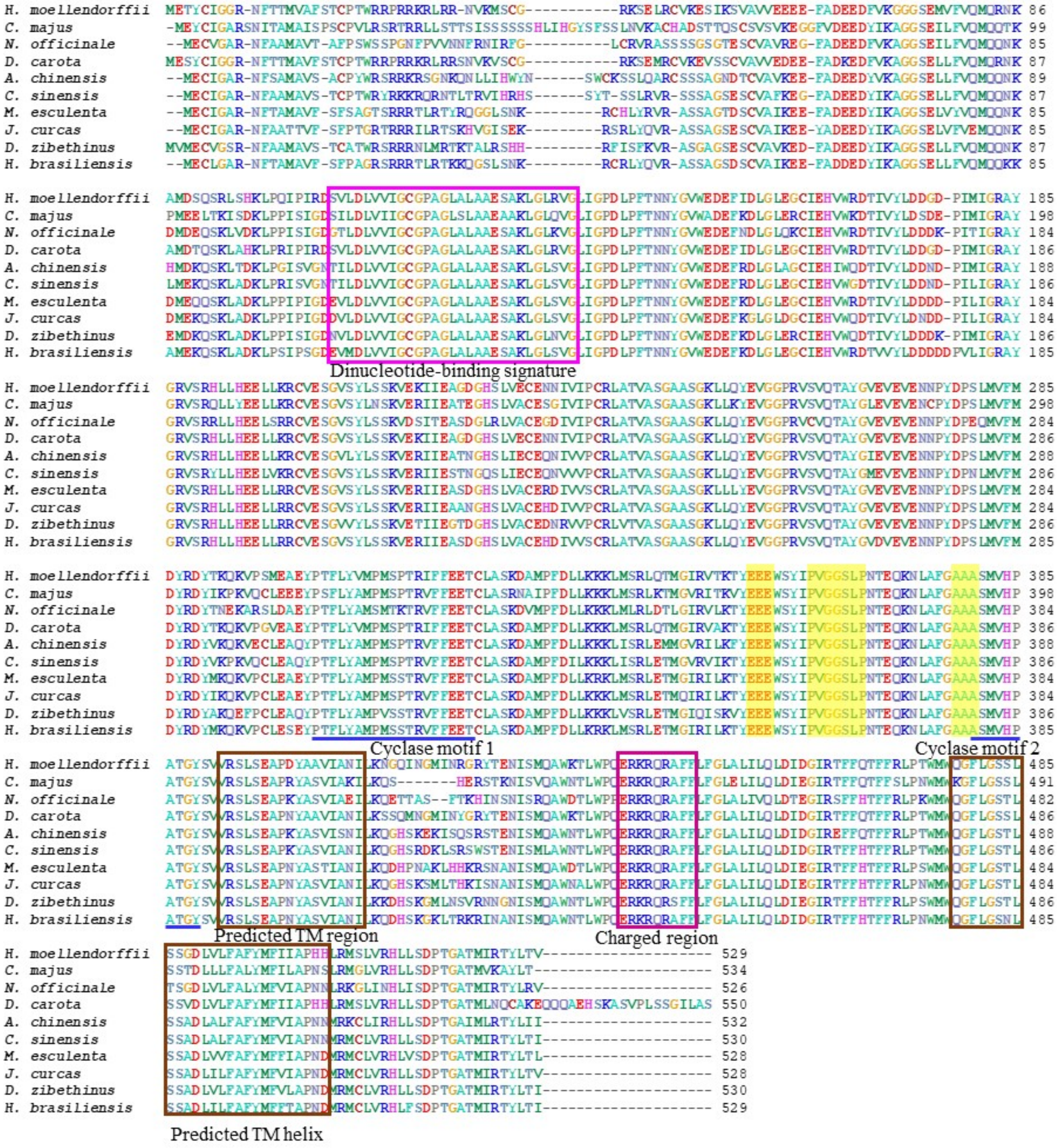

Figure S3H. Amino acid alignment of HmLCYE with selected corresponding genes was performed with the BioEdit program of ClustalW. Pink, brown, and purple boxes represent the dinucleotide-binding motif, predicted trans membrane (TM) helix, and charged region, respectively, and those with a blue underline represent cyclase motif 1 and 2. Yellow highlighted box indicate the conserved motif. *C. majus* (*Chelidonium majus*, MW307339), *N. officinale* (*Nasturtium officinale*, MT547986), *D. carota* (*Daucus carota*, KZM84177), *A. chinensis* (*Actinidia chinensis*, PSS20792), *C. sinensis* (*Camellia sinensis*, XP_028074216), *M. esculenta* (*Manihot esculenta*, XP_021596942), *J. curcas* (*Jatropha curcas*, XP_012076949), *D. zibethinus* (*Durio zibethinus*, XP_022751743), and *H. brasiliensis* (*Hevea brasiliensis*, XP_021637952) were involved in amino acid alignment of HmLCYE. A dash (–) represents an aligned gap.

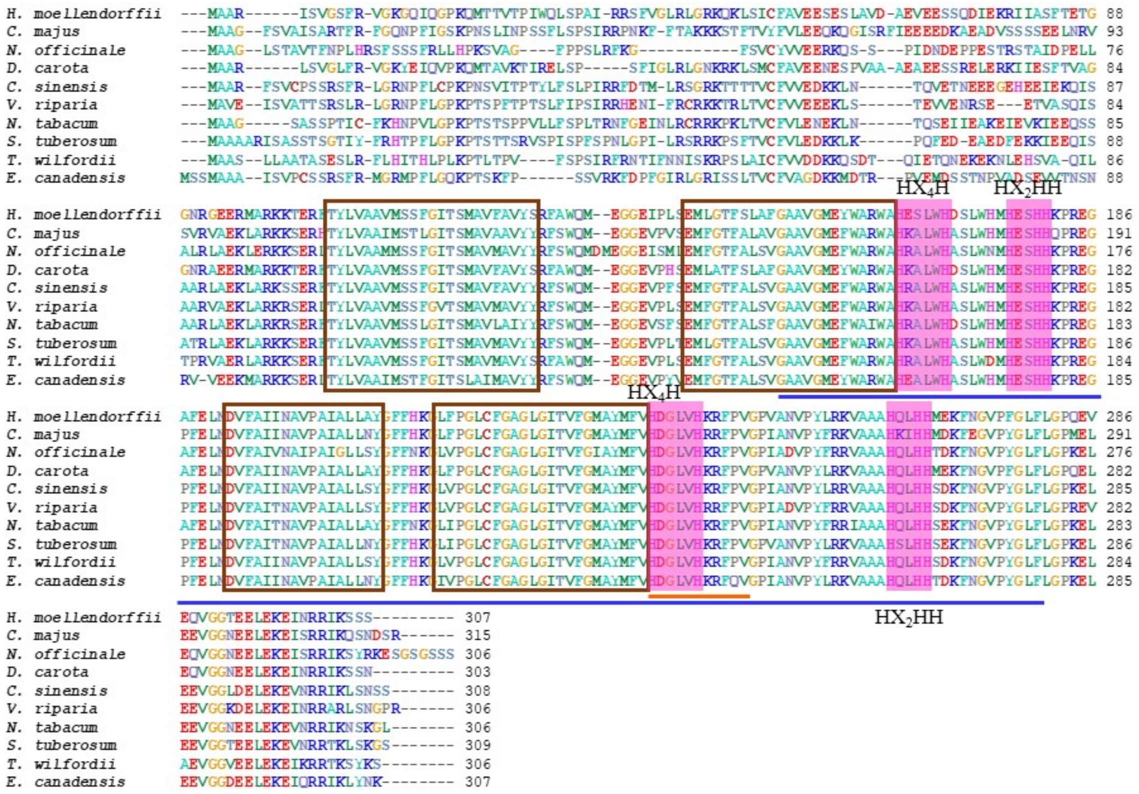

Figure S3I. Amino acid alignment of HmCHXB with selected corresponding genes was performed with the BioEdit program of ClustalW. The brown boxes indicate the transmembrane region. The histidine domain of the CHXB protein was highlighted in pink color. Blue and red underlined indicate the fatty acid hydroxylase domain and highly conserved region (motif 1). *C. majus* (*Chelidonium majus*, MW307333), *N. officinale* (*Nasturtium officinale*, MT547981), *D. carota* (*Daucus carota*, XP_017246058), *C. sinensis* (*Camellia sinensis*, XP_028112187), *V. riparia* (*Vitis riparia*, XP_034710058), *N. tabacum* (*Nicotiana tabacum*, XP_016467042), *S. tuberosum* (*Solanum tuberosum*, XP_006360197), *T. wilfordii* (*Tripterygium wilfordii*, XP_016467042), and *E. canadensis* (*Erigeron canadensis*, XP_043615242) were involved in amino acid alignment of HmCHXB. A dash (–) represents an aligned gap.

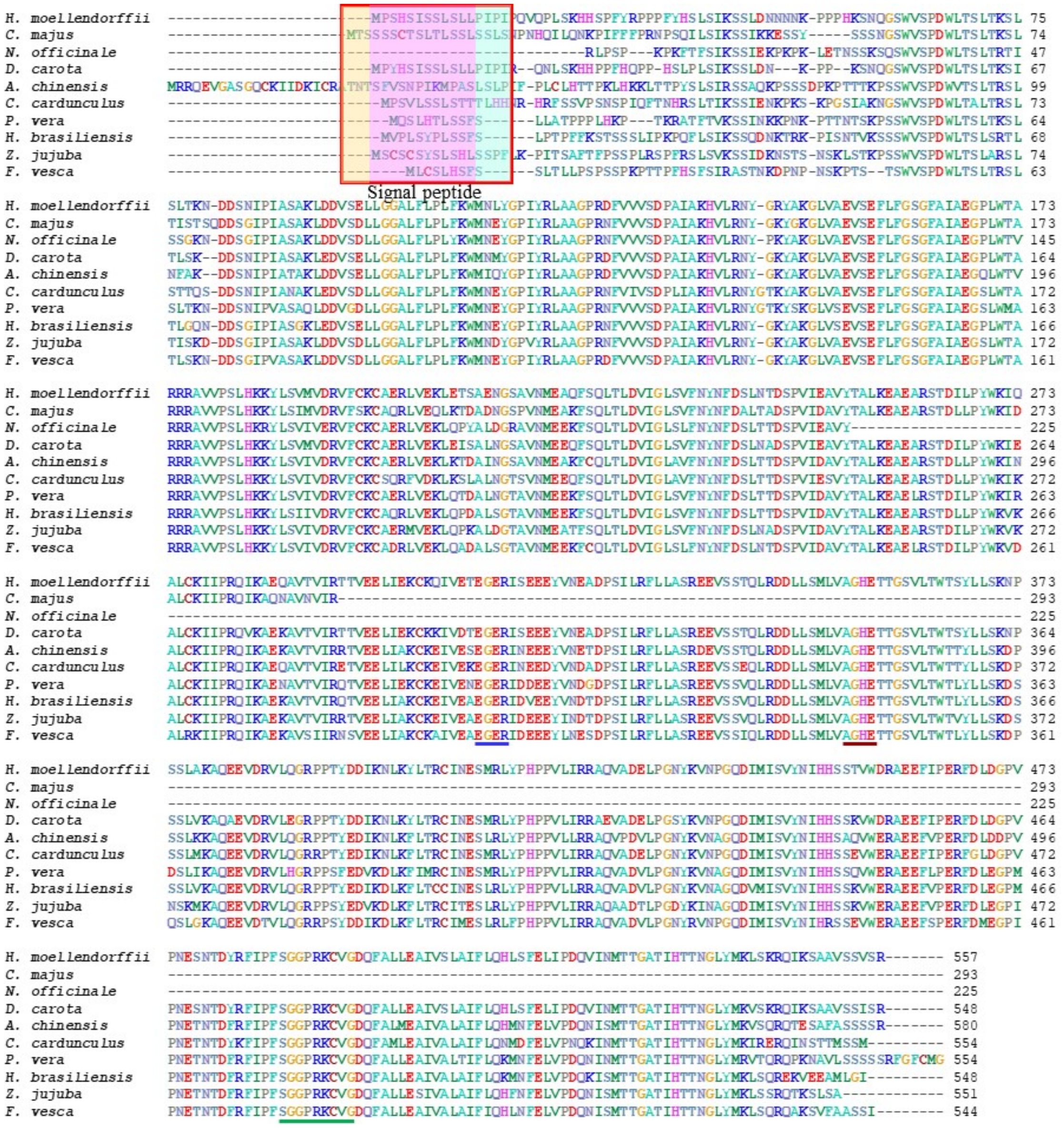

Figure S3J. Amino acid alignment of HmCHXE with selected corresponding genes was performed with the BioEdit program of ClustalW. The red boxes represent the signal peptide. The blue, yellow, and pink color highlighted denotes signal peptide C, N, and H regions of the CHXE protein, respectively. The sequence (AGHE) indicates center of α-helix which can bind to heme and the sequence (EGER) indicates salt bridges were shown in blue and red color, respectively. The green underline indicates the heme binding region. *C. majus* (*Chelidonium majus,* MW307335), *N. officinale* (*Nasturtium officinale*, MT547982), *D. carota* (*Daucus carota*, NP_001316100), *A. chinensis* (*Actinidia chinensis*, PSS04629), *C. cardunculus* (*Cynara cardunculus*, XP_024983120), *P. vera* (*Pistacia vera*, XP_031285161), *H. brasiliensis* (*Hevea brasiliensis*, XP_021677420), *Z. jujuba* (*Ziziphus jujuba*, XP_015898578), and *F. vesca* (*Fragaria vesca*, XP_004306170) were involved in amino acid alignment of HmCHXE. A dash (–) represents an aligned gap.

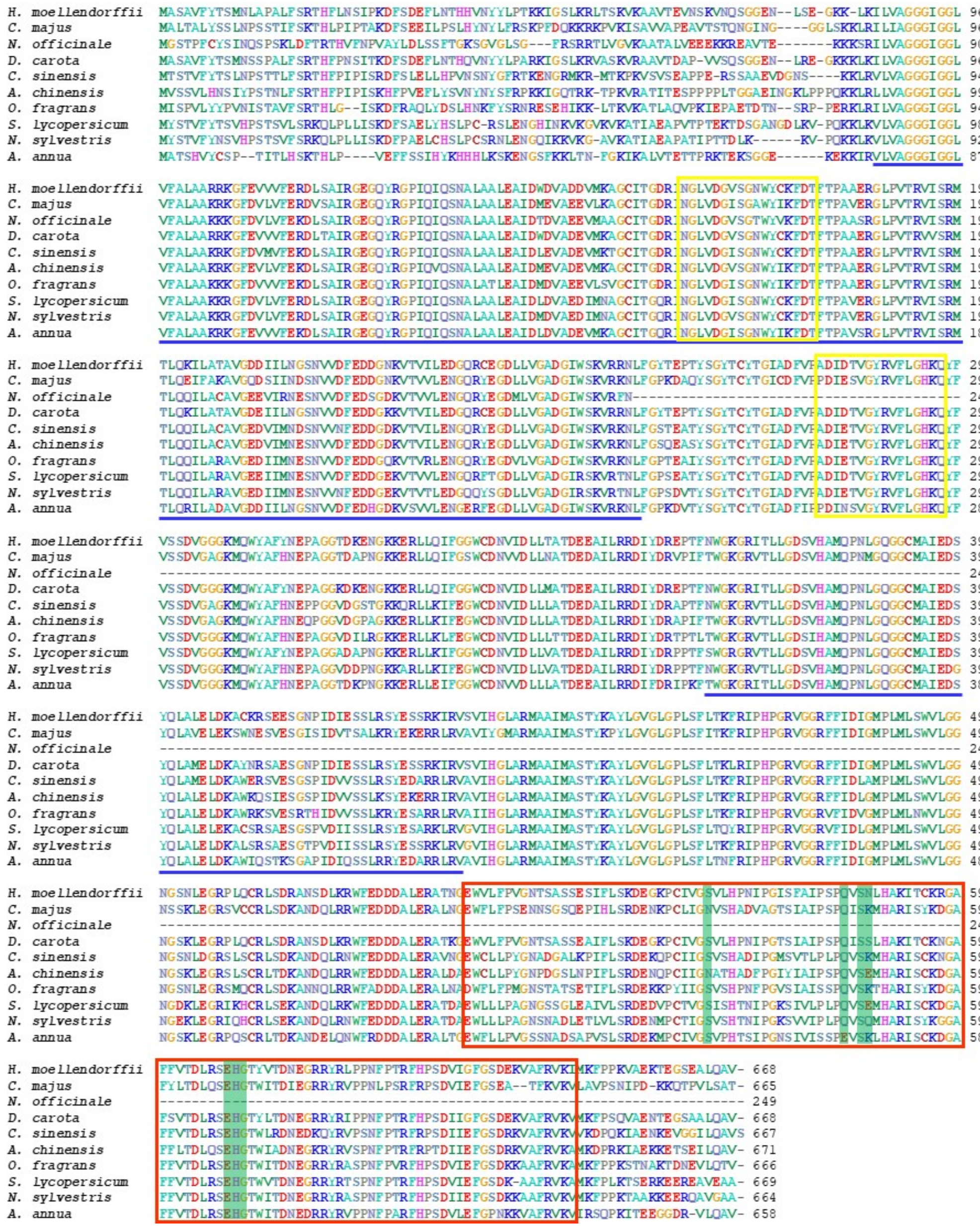

Figure S3K. Amino acid alignment of HmZEP with selected corresponding genes was performed with the BioEdit program of ClustalW. The yellow and red boxes represent the short motifs of lipocalin family proteins and forkhead-associated (FHA) domains, respectively. The phosphopeptide binding site of the ZEP protein was highlighted in green color. The blue underline represents the FAD-binding domain. *C. majus* (*Chelidonium majus,* MW307343), *N. officinale* (*Nasturtium officinale*, MT547990), *D. carota* (*Daucus carota*, KZM88660), *C. sinensis* (*Camellia sinensis*, AJB84624), *A. chinensis* (*Actinidia chinensis*, PSS21278), *O. fragrans* (*Osmanthus fragrans*, UBR90289), *S. lycopersicum* (*Solanum lycopersicum*, NP_001296233), and *N. sylvestris* (*Nicotiana sylvestris*, XP_009767383), and *A. annua* (*Artemisia annua*, PWA44217) were involved in amino acid alignment of HmZEP. A dash (–) represents an aligned gap.

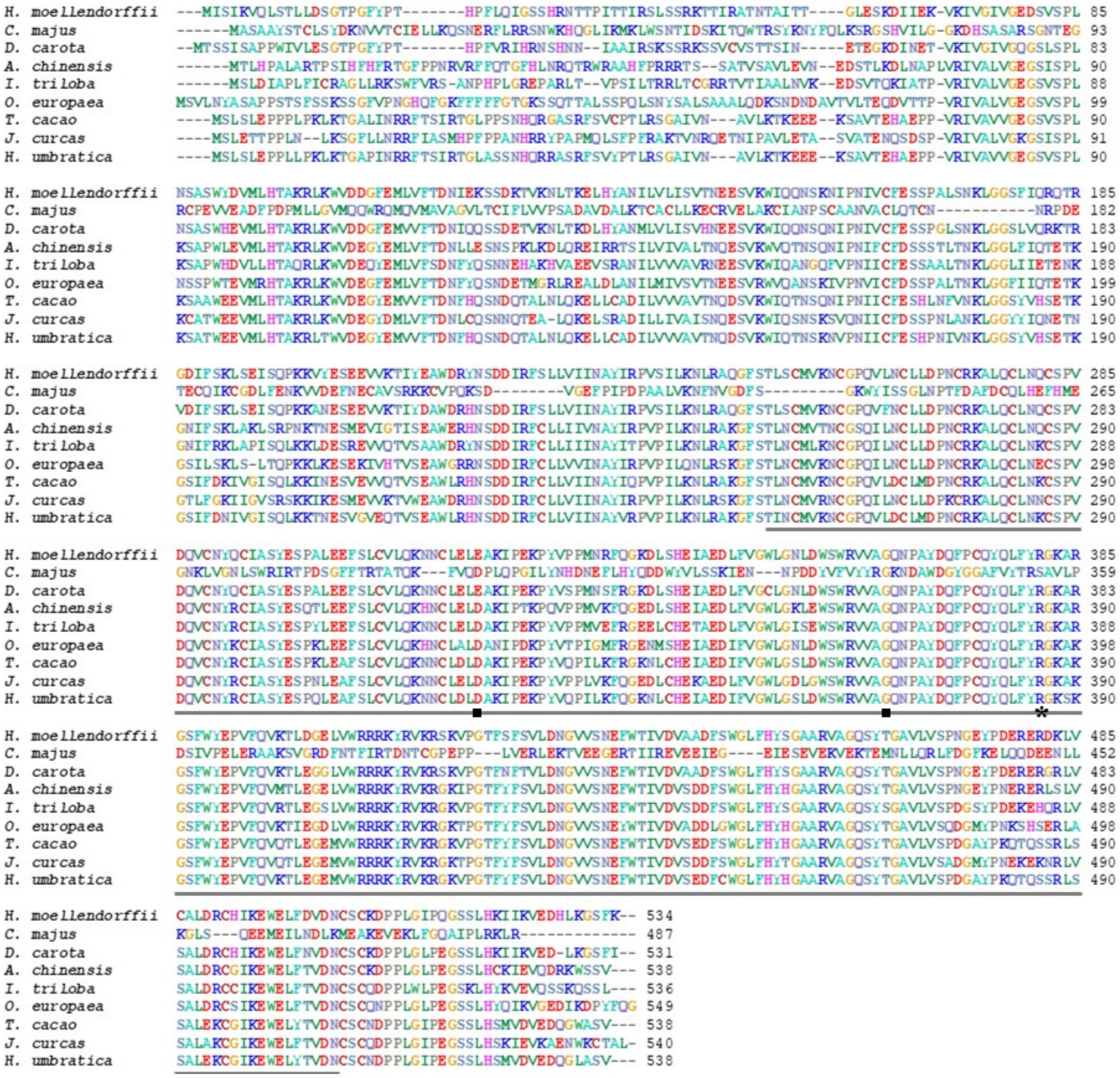

Figure S3L. Amino acid alignment of HmVDE with selected corresponding genes was performed with the BioEdit program of ClustalW. Ash underlined represent the lipocalins domains. Asterisks indicate the important residues for pH switch, and those with black boxes represent the putative active site. *C. majus* (*Chelidonium majus,* MW307342), *D. carota* (*Daucus carota*, XP_017241802), *A. chinensis* (*Actinidia chinensis*, PSS30264), *I. triloba* (*Ipomoea triloba*, XP_031110377), *O. europaea* (*Olea europaea*, CAA2974874), *T. cacao* (*Theobroma cacao*, EOX92314), *J. curcas* (*Jatropha curcas*, XP_012075261), *H. umbratica* (*Herrania umbratica*, XP_021274093), were involved in amino acid alignment of HmVDE. A dash (–) represents an aligned gap.

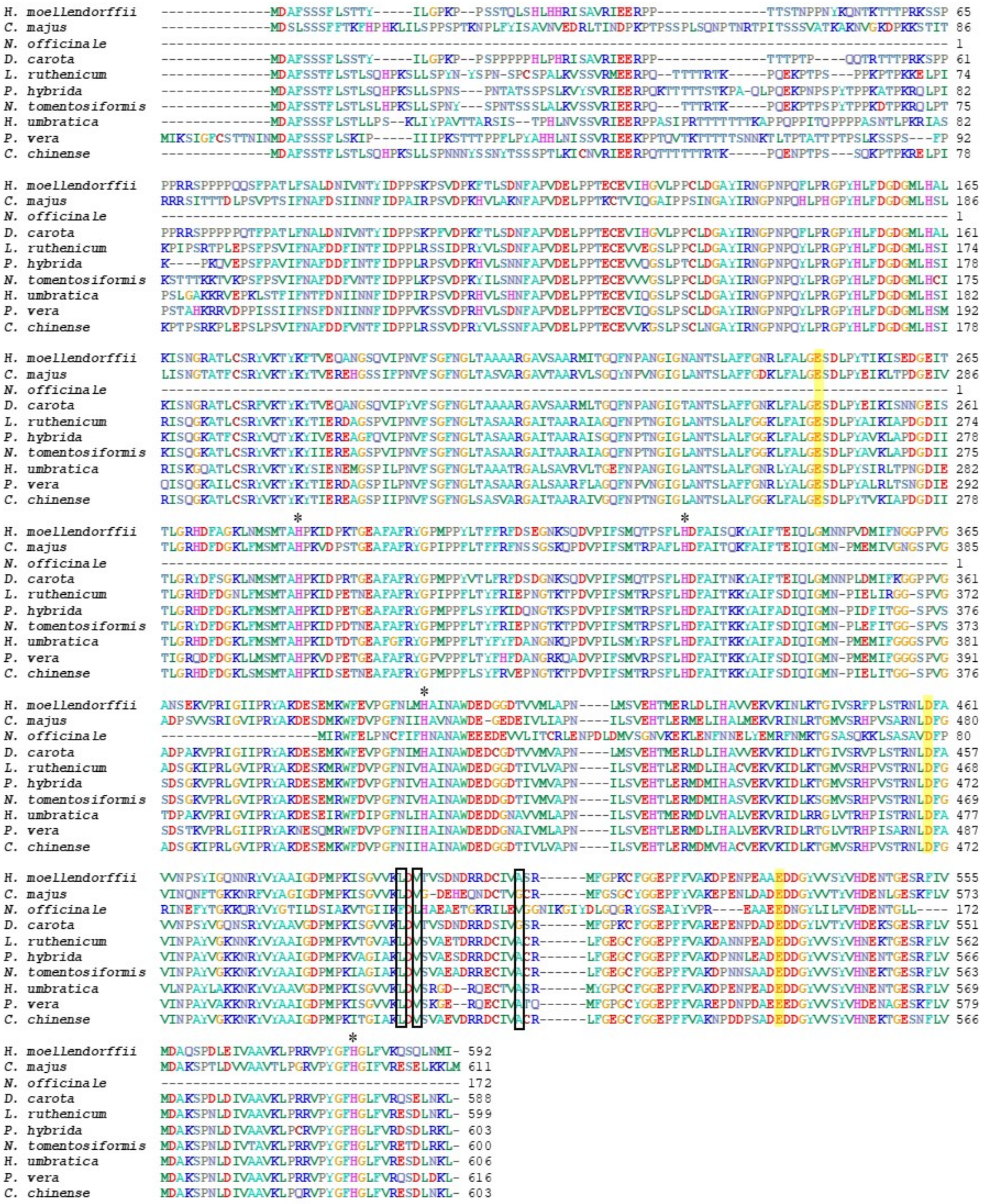

Figure S3M. Amino acid alignment of HmCCD with selected corresponding genes was performed with the BioEdit program of ClustalW. Asterisks indicate histidine residues involved in the coordination of the catalytic Fe^{2+}. The yellow highlighted boxes represent aspartate or glutamate residues which are predicted to be fixing the iron atom. The conserved amino acids present in the hydrophobic patches are shown in black boxes. *C. majus* (*Chelidonium majus,* MW307337), *N. officinale* (*Nasturtium officinale*, MT547984), *D. carota* (*Daucus carota*, XP_017227382), *L. ruthenicum* (*Lycium ruthenicum*, AIX87534), *P. hybrida* (*Petunia x hybrida*, QEH92024), *N. tomentosiformis* (*Nicotiana tomentosiformis*, XP_009611128), *H. umbratica* (*Herrania umbratica*, XP_021286209), *P. vera* (*Pistacia vera*, XP_031272013), and *C. chinense* (*Capsicum chinense*, PHU28865) were involved in amino acid alignment of HmCCD. A dash (–) represents an aligned gap.

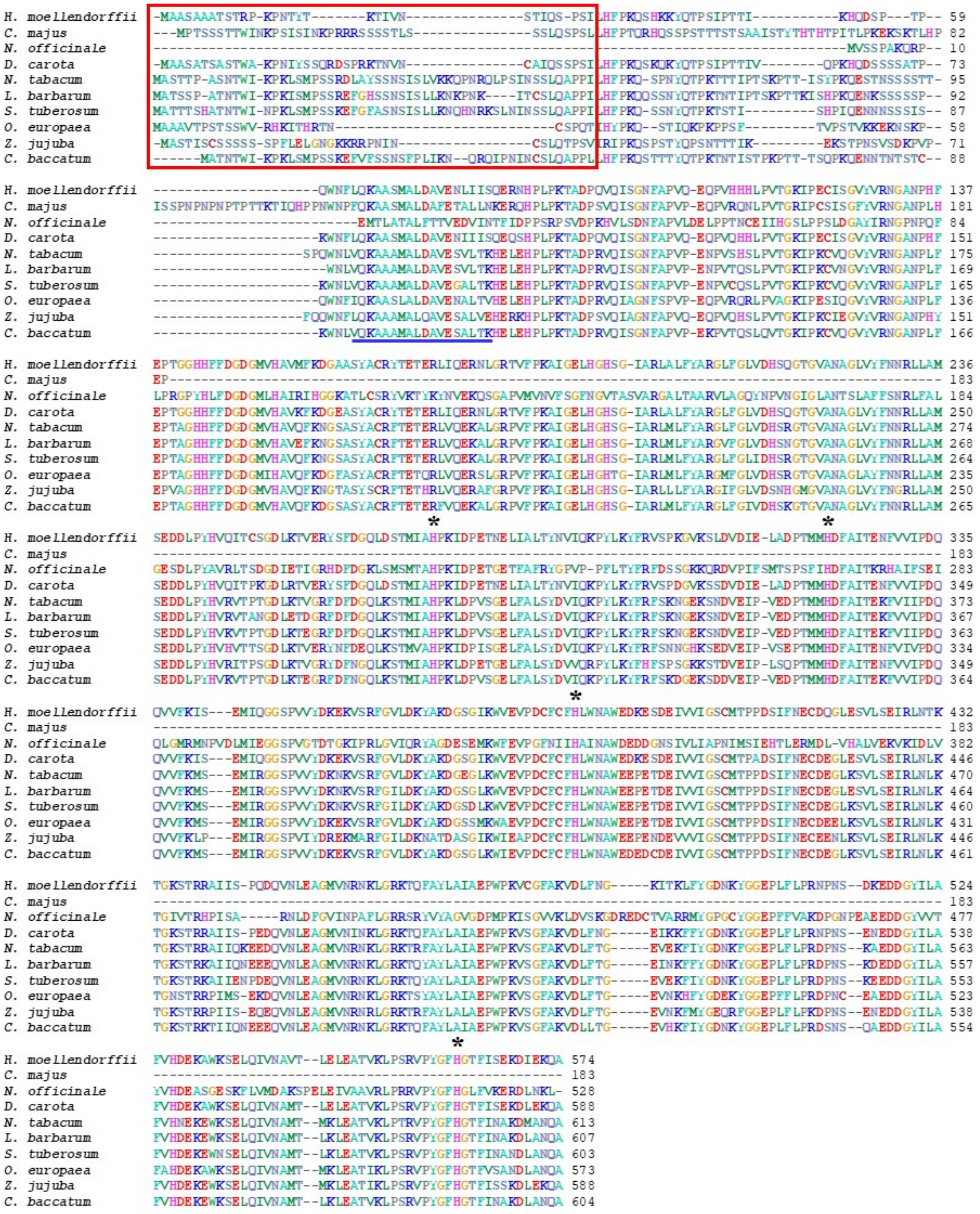

Figure S3N. Amino acid alignment of HmNCED with selected corresponding genes was performed with the BioEdit program of ClustalW. The red boxes represent putative chloroplast-targeting peptide. The α-amphipathic helix is shown in blue underlined. Asterisks indicate four conserved histidine required for activity. *C. majus* (*Chelidonium majus*, MW307340), *N. officinale* (*Nasturtium officinale*, MT547987), *D. carota* (*Daucus carota*, NP_001316098), *N. tabacum* (*Nicotiana tabacum*, NP_001312598), *L. barbarum* (*Lycium barbarum*, AIX87507), *S. tuberosum* (*Solanum tuberosum*, NP_001275103), *Olea europaea* (*Olea europaea*, XP_022848544), *Z. jujuba* (*Ziziphus jujuba*, XP_015893126) and *C. baccatum* (*Capsicum baccatum*, PHT43711) were involved in amino acid alignment of HmNCED. A dash (–) represents an aligned gap.

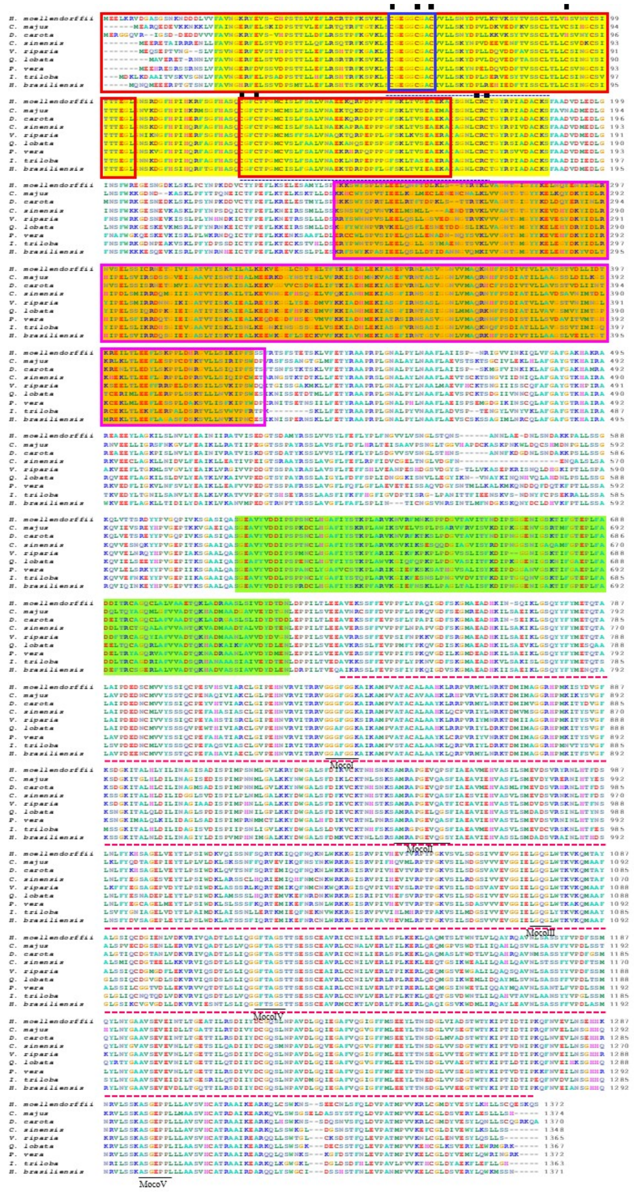

Figure S3O. Amino acid alignment of HmAO with selected corresponding genes was performed with the BioEdit program of ClustalW. The yellow, ash, orange, and green highlighted box represents the 2Fe-2S redox center-containing domain, 2Fe-2S ferredoxin-type iron-sulfur binding region, FAD-binding domain, and hammer-head domain, respectively. Asterisks indicate the eight conserved cysteine residues of [2Fe-2S] centers, and those with a red and pink rectangle box represent 2Fe-2S ferredoxin-type iron-sulfur binding domain and FAD-binding domain. The dotted blue and red underlined indicates that motif corresponding two non-identical [2Fe-2S] redox centers and molybdenum cofactor and substrate-binding domain. The putative motifs involved in Moco and substrate binding (MocoI-MocoV) are underlined in black continuous line. *C. majus* (*Chelidonium majus*, MW307332), *D. carota* (*Daucus carota*, XP_017257964), *C. sinensis* (*Camellia sinensis*, AVZ45846), *V. riparia* (*Vitis riparia*, XP_034688017), *Q. lobata* (*Quercus lobata*, XP_030960280), *P. vera* (*Pistacia vera*, XP_031272238), *I. triloba* (*Ipomoea triloba*, XP_031121886), and *H. brasiliensis* (*Hevea brasiliensis*, XP_021667834) were involved in amino acid alignment of HmAO. A dash (-) represents an aligned gap.

Table S1. Analysis of CP, XP, and AP Gene Sequences Using the SignalP and TMHMM Program

Gene names	Original shearing site (C score)	Synthesized shearing site (Y score)	Signal peptide (S score)	SignalP transmembrane peptide	TMHMM
HmPSY	0.160	0.175	0.305	No	1
HmPDS	0.113	0.125	0.151	No	0
HmZ-ISO	0.126	0.126	0.164	No	6
HmZDS	0.229	0.162	0.216	No	0
HmCrtISO	0.129	0.139	0.233	No	0
HmLCYB	0.212	0.140	0.106	No	0
HmLCYE	0.111	0.129	0.200	No	1
HmCHXB	0.115	0.108	0.123	No	3
HmCHXE	0.204	0.212	0.323	No	0
HmZEP	0.138	0.154	0.234	No	0
HmVDE	0.113	0.134	0.223	No	0
HmCCD	0.136	0.124	0.142	No	0
HmNCED	0.115	0.108	0.110	No	0
HmAO	0.123	0.107	0.108	No	0

Table S2. Analysis of CP, XP, and AP Gene Sequences Using the RADAR, NetNG-lyc 1.0, SOPMA, and PSIPRED Tools

Gene names	Internal repeats (RADAR)			N-glycosylation (NGLYC)		Secondary structure (SOPMA)			Disordered region (PSIPRED)	
	No. of repeats	Total score	Positions	No. of sites	Positions	Alpha helix	Extended strand	Random coil	No. of disordered region	Position
HmGGPS	2	77.48	158-181. 208-232	3	19 NHSN, NTSN, 33 NQSK	56.79%	6.37%	32.13%	2	5-64, 294-309
HmPSY	3	323.55	155-215, 216-320, 324-406	2	192 NASH, 401 NFTK	52.20%	10.50%	31.51%	3	1-91, 94-95, 101
HmPDS	2	217.23	53-125, 480-563	None	None	40.81%	15.19%	40.11%	2	7, 90-92
HmZ-ISO	2	77.16	85-107, 118-140	1	114 NSTG	46.15%	18.96%	31.59%	1	3-72
HmZDS	2	34.62	448-459, 497-506	2	44 NVSD, 257 NMSA	42.83%	14.51%	37.59%	3	57-59, 558, 565-567
HmCrtISO	2	278.04	318-411, 442-538	8	19 NTSL, 31 NNSS, 32 NSST, 57 NLSL, 202 NLSV, 315 NASM, 388 NATR, 563 NTTA	37.44%	18.08%	38.72%	2	1-102, 625
HmLCYB	2	275.46	9-100, 259-364	None	None	38.42%	17.62%	38.42%	4	5-61, 64-65, 69-71, 505
HmLCYE	2	271.27	326-409, 441-527	2	10 NFTT, 426 NISM	38.94%	19.28%	38.56%	3	5-57, 100, 529
HmCHXB	2	161.34	26-83, 248-306	None	None	48.21%	16.94%	29.32%	7	4-30, 32, 35-37, 65, 82-93, 95, 303
HmCHXE	3	111.57	4-27, 33-59, 65-89	5	212 NGSA, 372 NPSS, 408 NESM, 475 NESN, 525 NMTT	45.06%	10.05%	40.75%	5	43, 48-49, 52-61, 308-332, 334-336
HmZEP	2	254.17	320-409, 512-594	5	71 NQSG, 77 NLSE, 213 NGSN, 497 NGSN, 543 NTSA	30.99%	19.16%	44.46%	1	36-81
HmVDE	2	92.40	334-360, 379-408	3	34 NTTP, 127 NLTK, 503 NCSC	30.52%	21.72%	40.82%	6	4-45, 48, 50-59, 61-

									62, 177, 514-515
HmCCD	5	389.00	17-70, 71-142, 143-231, 232-299, 300-375	5	190 NGSQ, 234 NTSL, 277 NMSM, 315 NKSQ, 464 NPSY	16.22%	22.13%	56.08%	2	5-75, 592
HmNCED	2	222.11	203-276, 494-571	1	23 NTSI	14.98%	23.17%	56.97%	2	3-60, 574
HmAO	2	225.58	303-380, 801-881	11	261 NFTD, 298 NVSE, 308 NETG, 574 NLSN, 652 NKSP, 940 NHSN, 943 NKSA, 1016 NFSQ, 1173 NLSA, 1327 NSSE, 1333 NLSF	33.67%	18.00%	41.76%	8	16-17, 63, 150-151, 153-159, 179-230, 557-587, 591-593, 1237

Table S3. Percentage Identity (%) Analysis of Amino Acid Sequences, Comparing Between *H. moellendorffii* CP, XP, and AP Genes and Other CP, XP, and AP Amino Acid Sequences. Accession Numbers of the Sequences are Provided in Figure S2 and Table S4

Species name	*GGPS*	*PSY*	*PDS*	*Z-ISO*	*ZDS*	*CrtISO*	*LCYB*	*LCYE*	*CHXB*	*CHXE*	*ZEP*	*VDE*	*CCD*	*NCED*	*AO*
Higher plants															
H. moellendorffii	100	100	100	100	100	100	100	100	100	100	100	100	100	100	100
A. thaliana	69.39	74.51	79.35	70.64	81.05	73.90	78.76	74.80	64.36	79.35	71.77	65.22	38.99	37.50	60.84
B. napus	70.45	72.95	78.52	71.47	80.79	79.48	80.69	74.23	65.97	80.23	69.39	19.03	38.51	72.16	61.06
B. oleracea	69.71	72.95	78.87	70.00	81.33	79.51	80.48	74.57	65.97	80.65	69.77	19.03	67.88	71.10	61.18
B. rapa	70.15	73.01	78.52	70.87	81.15	79.65	80.49	73.75	65.28	80.85	69.15	18.47	38.90	72.16	59.05
C. rubella	65.77	73.85	78.95	69.68	80.07	78.93	80.16	75.24	66.20	79.89	69.09	17.98	39.57	71.40	60.80
C. sativa	63.77	73.37	78.95	69.94	79.75	76.84	78.40	74.28	65.17	79.70	67.83	72.37	38.57	71.45	59.36
C. sinensis	71.59	75.38	80.93	71.82	85.09	79.55	85.12	78.24	71.33	82.66	76.70	18.87	71.38	74.56	71.73
Citrus sinensis	67.25	73.27	81.04	68.93	84.67	77.61	83.90	74.57	65.41	80.30	70.76	18.93	67.64	73.35	66.79
D. carota	87.01	92.47	92.35	90.66	94.41	93.98	97.03	93.57	87.79	93.61	93.25	86.04	88.95	91.99	89.19
E. salsugineum	68.66	73.19	78.62	70.09	80.97	80.07	80.68	76.16	64.83	79.77	70.17	18.36	38.33	71.38	60.41
J. curcas	68.81	78.09	79.14	72.73	81.82	78.17	86.31	79.12	66.78	80.30	71.79	66.79	38.15	73.52	65.84
N. tabacum	71.71	77.65	78.39	71.71	83.54	79.15	83.80	74.81	68.38	79.66	74.92	67.88	70.96	78.18	65.43
P. vera	68.48	76.89	74.50	72.80	86.20	78.81	82.74	77.39	67.47	82.24	74.58	16.71	69.52	71.15	68.38
R. sativus	70.21	74.03	78.73	69.71	81.44	78.72	79.51	74.42	64.60	80.04	69.07	21.55	39.29	70.27	60.41
S. lycopersicum	68.90	76.96	77.72	73.85	83.19	79.05	83.00	72.36	67.81	79.93	75.26	68.02	68.87	77.56	65.58
S. oleracea	66.85	73.49	80.07	69.23	80.96	77.63	81.09	72.11	65.77	79.85	67.67	18.33	65.08	68.14	64.80
Bacteria															
C. bacterium					64.93	59.20	35.22	35.73							31.78
F. bacterium										31.15					
G. bacterium							35.99	36.41							
Limnothrix sp.	57.72														
Microcoleus sp.		58.47												39.17	
P. bacterium									41.07						32.30
P.marinus			66.95												
Chlorophyta															
C. sorokiniana			69.17	50.55	61.14	52.82	50.10			56.78	50.23	39.49	33.52		33.04
D. salina	58.15	55.13					46.39	41.67	48.04						33.07

H. lacustris	58.10	56.49	67.05				50.00	41.05		57.46	49.08	44.54			
M. conductrix	63.29	54.72	66.60	53.44	61.90	53.57				58.78	48.97	41.72	32.80		33.07
Dinoflagellates															
K. veneficum											34.06				
S. microadriaticum		35.99	46.87			26.46						23.53		26.07	31.07
S. natans		35.52					34.69					22.96			
S. necroappetens		36.36	49.37			26.83				30.18		24.88		25.55	30.91
Symbiodinium sp.			50.65									24.25	27.21		31.67
Heterokonts															
D. viridis	52.55	42.82	58.53			31.12									
E. siliculosus			58.32		52.17		36.45								
F. solaris				48.30	50.00						36.17	20.00			
H. fermentalgiana									33.16				21.78		
N. gaditana	51.59				53.54		34.31			39.60		17.72		33.12	33.50
U. pinnatifida			58.97		52.42										

Table S4. GenBank Accession Number of CP, XP, and AP Amino Acid Sequences of Other Higher Plants Used for Concatenated Phylogenetic Tree Construction

Species name	*GGPS*	*PSY*	*PDS*	*Z-ISO*	*ZDS*	*CrtISO*	*LCYB*	*LCYE*	*CHXB*	*CHXE*	*ZEP*	*VDE*	*CCD*	*NCED*	*AO*
A. thaliana	NP_195399	AAM62787	NP_001319934	NP_563879	NP_001319473	NP_001322208	AAA81880	AAB53336	NP_194300	AAM13903	AAL91193	NP_565520	NP_191911	NP_193652	NP_189946
B. napus	XP_013722211	AEN94302	NP_001302666	XP_013645558	XP_022559356	XP_013716452	NP_001303063	NP_001302606	AGL08131	XP_022553285	NP_001302817	NP_001302836	NP_001302789	XP_013736250	XP_013744009
B. oleracea	XP_013596174	XP_013613175	XP_013636271	XP_013638489	XP_013586764	XP_013599462	XP_013586400	XP_013622022	XP_013617332	XP_013591860	XP_013597987	XP_013586635	XP_013600512	XP_013623026	XP_013636205
B. rapa	XP_009141710	XP_009121405	XP_009109564	XP_009148420	XP_009124205	XP_009118478	XP_009146860	XP_009120221	NP_001288890	XP_009125249	XP_009103460	XP_009148110	XP_009138676	XP_033142300	XP_009119278
C. rubella	XP_006298002	XP_006287812	XP_006283428	XP_006305142	XP_006297343	XP_006307051	XP_023642504	XP_006280298	XP_006284181	XP_023638222	XP_006280131	XP_006307456	XP_023638364	XP_006297265	XP_006293569
C. sativa	XP_010510773	XP_010453906	XP_010435176	XP_010475961	XP_019096950	XP_010480371	XP_010486621	XP_010451740	XP_010438839	XP_010426945	XP_010484561	XP_010416942	XP_010413132	XP_010487250	XP_010510805
C. sinensis	XP_028108146	ABQ23179	AHB32104	XP_028081203	AJB84621	XP_028055858	AJB84622	XP_028074216	XP_028112187	AJB84623	AJB84624	XP_028102444	XP_028127311	AUD40389	AVZ45846
Citrus sinensis	XP_006466719	NP_001275815	AEQ29524	XP_006478893	NP_001275793	XP_006481644	AAU05146	XP_006475492	NP_001275830	XP_006491795	XP_006466600	BAO18772	XP_006484631	NP_001275864	XP_006487801
D. carota	XP_017246904	NP_001316096	NP_001316104	XP_017218756	NP_001316091	AGZ61882	KZM92091	KZM84177	XP_017246058	NP_001316100	KZM88660	XP_017241802	XP_017227382	NP_001316098	XP_017257964
E. salsugineum	XP_006409129	XP_006400253	XP_006414767	XP_006417412	XP_006408139	XP_006417877	XP_006407592	XP_006401252	XP_006413280	XP_006403722	XP_006393901	XP_006417674	XP_006402261	XP_006407071	XP_024015827
J. curcas	XP_012079090	XP_012074703	XP_012078425	XP_012074764	NP_001295634	XP_012074260	XP_012085981	XP_012076949	XP_012090186	XP_020538220	XP_012079233	XP_012075261	XP_012065572	XP_012084339	XP_037491510
N. tabacum	XP_016435651	AHA58684	XP_016498101	XP_016476688	NP_001312062	NP_001313210	XP_016489181	XP_016467034	XP_016467042	XP_016475396	XP_016476042	XP_016466133	NP_001312446	NP_001312598	XP_016514146
P. vera	XP_031253139	XP_031262932	XP_031249016	XP_031254779	XP_031275399	XP_031260465	XP_031268877	XP_031255713	XP_031249306	XP_031285161	XP_031254414	XP_031262539	XP_031272013	XP_031247481	XP_031272238
R. sativus	XP_018467471	XP_018442662	XP_018477228	XP_018490049	XP_018493360	XP_018432638	XP_018492024	XP_018472983	XP_018478035	XP_018442532	XP_018441511	XP_018466588	XP_018459655	XP_018492901	XP_018483492
S. lycopersicum	NP_001234268	NP_001234671	NP_001234095	NP_001334551	NP_001234383	NP_001296159	NP_001234226	NP_001234337	NP_001234348	ACJ25967	NP_001296233	XP_004237473	XP_004246004	NP_001234455	XP_004228468
S. oleracea	XP_021847844	XP_021839188	XP_021847448	XP_021861125	XP_021858704	XP_021858982	XP_021858726	XP_021851843	XP_021844219	XP_021848892	XP_021837806	XP_021853497	XP_021851772	XP_021867001	XP_021864564

Table S5. List of Primers Used in the qRT-PCR Analysis to Determine mRNA Expression Levels of *H. moellendorffii* CP, XP, and AP genes

Primer names	Primer sequence (5′→3′)	PCR product length (bp)
HmGGPP-F	TGTCCGTCCAATGCTTTGTATC	143
HmGGPP-F	AGATCATCGTTATCCATACAAGGC	
HmPSY-F	ACGAGGAATTAGAGGTTAAGCC	134
HmPSY-R	GGTCATCAGTAGTGTTCCCAAG	
HmPDS-F	TGATATTCGCTATGCCTAACAAGC	127
HmPDS-R	TTCTCAGGCCATGTTAGCATTTC	
Hm-Z-ISO-F	TGATAATTCTACTGGGCTTGGTC	118
Hm-Z-ISO-R	TAAGCCACTATGGACACCAGC	
HmZDS-F	ACTTGTTGACCCAGATGGAGC	104
HmZDS-R	TTTGGATACTCTTGCGTGTGC	
HmCrtISO-F	CACCGAACTCACAAGCAAATTTC	112
HmCrtISO-R	CTCGAGTGACTTCAGTTCCAATG	
HmLCYB-F	TGGGTGGATGAGTTTGAGGC	100
HmLCYB-R	CAAGCTCCTTGGTTGTCTCG	
HmLCYE-F	TAATTATGGCGTATGGGAGGATG	138
HmLCYE-R	CGACTAACTCTTCCATAAGCACG	
HmCHXB-F	TGCAGAAGTAGAAGAGAGTTCGC	126
HmCHXB-R	ACCAAATAAGTGAACCGCTCTG	
HmCHXE-F	TCGACGTTATTGGTCTATCAGTG	122
HmCHXE-R	TAATATGTCAGTTGATCGAGCCTC	
HmZEP-F	AGAAGCTATTGATTGGGATGTTGC	102
HmZEP-R	TACCAATTGCCAGAAACACCATC	
HmVDE-F	ACCAAGGAGCTACATTATGCAAAC	134
HmVDE-R	TTACTTAGTGCTGGAGATGACTCG	
HmCCD-F	TGCAAACACTAGCTTGGCATTC	148
HmCCD-R	TGTCATGCTCATGTTAAGCTTTCC	
HmNCED-F	AACTGAAAGACTAATCCAGGAGC	146
HmNCED-R	TACCCTGAGAATGATCAACAAGTC	
HmAO-F	TACTCGGACTAAGTTAGTTGCTGG	143
HmAO-R	TTGCTCCAATTACTATCCCTGTC	
Hmα-tubulin-F	AACCTGATGGACAAATGCCTG	101
Hmα-tubulin-R	CGAGGTACATGCTTTCCAGC	
